# Atomic Layer Electrodeposition of a Pt Monolayer on Au via Surface-Specific PtCl_4_^2**–**^ Adsorption

**DOI:** 10.1021/acs.chemmater.6c01182

**Published:** 2026-07-16

**Authors:** Hyunki Kim, Thomas P. Moffat

**Affiliations:** Materials Science and Engineering Division, 10833 National Institute of Standards and Technology, Gaithersburg, Maryland 20899, United States

## Abstract

Atomic layer deposition of Pt monolayer films on Au is demonstrated based on the irreversible adsorption of square planar PtCl_4_^2–^_aq_ in the flat configuration, followed by reduction at low overpotentials in NaCl-based electrolytes. The strong affinity of PtCl_4_^2–^_aq_ for Au at positive potentials, +0.40 V_SSCE_, contrasts with its negligible interaction with Cl^–^ covered Pt. The saturated fractional surface coverage of PtCl_4_^2–^_ads_ on (111)-textured Au thin films is ≈0.13 in freshly prepared 0.1 mol/L HClO_4_, 0.12 in 0.5 mol/L NaCl, and 0.104 in 4 mol/L NaCl, as determined by X-ray photoelectron spectroscopy. When the reduction of PtCl_4_^2–^_ads_ is restricted to potentials where adsorption is still substrate-selective, Pt nucleation and growth are limited to monolayer dimensions. Three different deposition modalities are examined: potentiostatic, voltammetric, and pulsed potential, and the specific parameters to deposit monolayer films are identified. Pulsed potential deposition enables the adsorption and reduction steps to be temporally separated and quantitatively evaluated.

## Graphical abstract

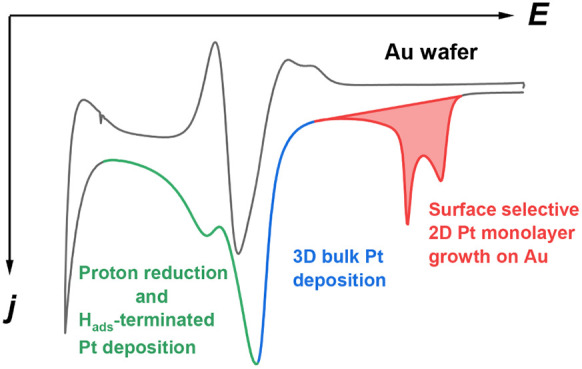

## Introduction

Over the last few decades, significant effort has been given to exploring the thrifty use of Pt group metals as electrocatalysts. Of particular interest are ultrathin films and analogous core–shell nanoparticles, where the deposition of a monolayer of Pt group metals enables both minimization of precious metal loading and exploration of enhanced catalysis via substrate-induced structural and electronic perturbation of the precious metal overlayer.^1–3^ Wet processes ranging from electrode derivatization with Pt complexes to galvanic exchange reactions, to H_ads_, CO_ads_, or I_ads_-terminated electrodeposition, along with dry processes such as sputtering and atomic layer deposition (ALD), have been used to synthesize ultrathin Pt films and explore their resulting catalytic activity.^1–39^

The structure of the overlayers is sensitive to both intrinsic and extrinsic processing conditions, with the deposition of smooth, fully coalesced Pt films difficult to realize. These limitations motivate the need for a more detailed examination of the impact of the overpotential and precursor concentration on the relevant nucleation and growth processes.

The electrochemistry of Pt halide complexes and its variations with oxidation state and different ligands has received significant study.^37–43^ The most detailed knowledge of the earliest stages of Pt nucleation and growth has emerged from electrochemical scanning tunneling microscopy (ECSTM) experiments.^4,6–9,19,21,23–25^ These studies provide important structural insights into the adsorption of PtCl_4_^2–^_ads_, PtCl_3_(H_2_O)^−^_ads_, PtCl_6_^2–^_ads_, and Pt_*x*_CO_*y*_^n–^_ads_ on different Au surfaces and their subsequent reduction to form submonolayer quantities of Pt on Au.^1–30^ These studies reveal that a saturation coverage of the Pt complexes occurs below 0.15 coverage of the Au(111) surface. However, it is still unclearhow these adsorbates are converted into two-dimensional Pt clusters and what determines and even restricts further Pt deposition from occurring remains to be explained.

Extended X-ray fine structure (EXAFS) studies indicate that the adsorption of PtCl_4_^2–^ occurs in a flat square planar configuration.^29–31^ Application of resonance surface X-ray scattering (RSXRS)^11,15^ reveals that Pt deposition from H_2_PtCl_6_ in HClO_4_ is highly potential-dependent. At 0.90 V_RHE_, the Pt overlayer saturates at ∼1.4 monolayers, while lower overpotentials lead to saturation at submonolayer coverage while higher overpotentials trigger continuous three-dimensional growth with increasing roughness. The above processes were rationalized as proceeding through the adsorption of PtCl_6_^2–^ on both Au and Pt surface segments. Despite these advances, the underlying basis for the observed behavior remains to be elucidated.

Herein, the mechanistic basis of Pt deposition on Au in halide media is explored using a combination of electroanalytical methods, X-ray photoelectron spectroscopy (XPS), and scanning tunneling microscopy (STM), with particular focus on how adsorption and reduction processes govern Pt nucleation and growth. Adsorption and metal deposition from PtCl_4_^2–^_aq_ precursor is examined with the square planar divalent complex identified by XPS as the adsorbed intermediate at small overpotentials, regardless of the oxidation state of different chloro-complex precursors. PtCl_4_^2–^_aq_ adsorption in halide supporting electrolyte competes with Cl^–^, with the respective coverages being concentration, potential-, and substrate-dependent. XPS confirms irreversible PtCl_4_^2–^_ads_ adsorption on (111)-textured Au thin film, while strongly adsorbed Cl^–^ on Pt at the same potentials apparently blocks PtCl_4_^2–^_ads_ adsorption. Significantly, the PtCl_4_^2–^_ads_ surface complex on Au can be reduced at potentials that precede significant Cl^–^ desorption from Pt surfaces. Accordingly, it is the surface-selective nature of PtCl_4_^2–^_ads_ adsorption that enables Pt deposition on Au to be restricted to monolayer dimensions. Building upon these findings, the impact of the adsorption and reduction pathways on metal deposition is examined under different potentiodynamic and potentiostatic conditions.

## Results and Discussion

### Surface-Selective Electrodeposition

Pt electrodeposition on Au in 0.5 mol/L NaCl or 4.0 mol/L NaCl containing (0.01 to 10) mmol/L K_2_PtCl_4_ (pH 3.5) involves several distinct processes, as indicated in Figure 1. Equilibrium speciation of the (PtCl_*x*_(H_2_O)_4‑x_)^(x‑2)–^_aq_ complexes is dependent on the halide concentration. For 0.5 mol/L NaCl, PtCl_4_^2–^_aq_ is the dominant species, accounting for 94%, with PtCl_3_(H_2_O)^−^_aq_ largely comprising the remainder, as summarized in Figure S1. Increasing the halide concentration to 4.0 mol/L NaCl further stabilizes PtCl_4_^2–^_aq_ to account for 99% of the population. The reported standard reversible potential for the PtCl_4_^2–^ + 2e^–^ → Pt + 4Cl^–^ reaction ranges from 0.522 V_SSCE_ to 0.493 V_SSCE_.^40–44^ Using the latter value yields +0.469 V_SSCE_ for the reversible potential of Pt in 1 mmol/L PtCl_4_^2–^_aq_ in 0.5 mol/L NaCl and +0.362 V_SSCE_ for 4.0 mol/L NaCl.

**Figure 1:**
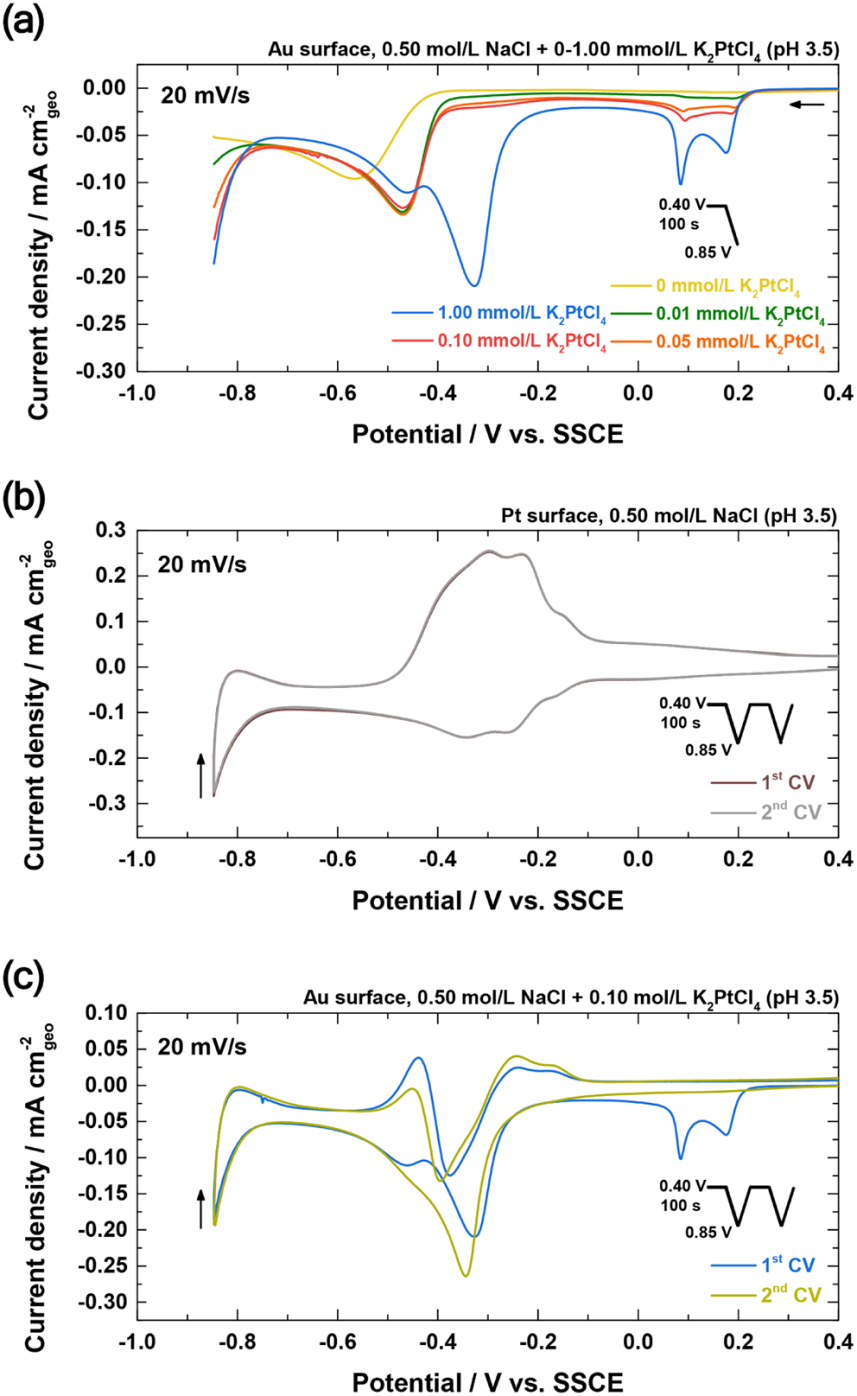
Cyclic voltammetry reveals surface-selective Pt deposition on Au below 0.3 V_SSCE_ followed by the onset of three-dimensional (3D) Pt growth on Pt below −0.2 V_SSCE_ before its termination by H_ads_ below −0.7 V_SSCE_. (a) First voltammetric cycle of Pt deposition on Au in 0.5 mol/L NaCl containing (0.01 to 10.0) mmol/L K_2_PtCl_4_ after a 100 s dwell at 0.40 V_SSCE_. (b) Voltammetry of H_upd_ on a Pt electrode overlaps the potential region where 3D Pt deposition occurs in 0.5 mol/L NaCl. (c) Two consecutive voltammograms in 0.5 mol/L NaCl with 1.00 mmol/L K_2_PtCl_4_ highlight the two distinctive surface-selective waves for Pt deposition on Au.

For voltammetric experiments, piranha-cleaned (Caro’s acid)^45^ Au thin-film electrodes were immersed at 0.40 V_SSCE_ where adsorption of PtCl_4_^2–^ is anticipated based on prior ECSTM studies (albeit performed in perchloric and sulfuric acid).^7,8,28^ After idling for 100 s, voltammetry was initiated, and two overlapping reduction waves were evident near +0.175 V_SSCE_ and +0.08 V_SSCE_ in Figure 1a. The two peaks increased monotonically with K_2_PtCl_4_ concentration, which is attributed to reduction of the initially adsorbed PtCl_4_^2–^_ads_ at 0.40 V_SSCE_ and subsequently from solvated PtCl_4_^2–^_aq_ species that adsorb on the freshly available Au sites.

Near −0.20 V_SSCE_, sustained Pt-on-Pt deposition initiates and increases rapidly, subject to the available PtCl_4_^2–^_aq_ flux.^46^ This transition from surface-selective two-dimensional growth to three-dimensional Pt deposition coincides with disruption of the halide adlayer whence the deposition rate increases rapidly with potential. Displacment of the adsorbed halideis also congruent with development of a hydrogen-covered Pt surface.^47^ This is evident from the overlap between the Pt deposition peak near −0.32 V_SSCE_ in Figure 1a and the H_upd_ waves on polycrystalline Pt seen in the neat 0.5 mol/L NaCl supporting electrolyte in Figure 1b. Below −0.40 V_SSCE_, Pt deposition is quenched as the surface becomes saturated with H_ads_.^16,37,46^ On the reverse sweep, metal deposition reactivates with the oxidative removal of H_ads_ as revealed in Figure 1c.

A second voltammetric scan in Figure 1c and Figure S2 was initiated following a 100 s pause at +0.40 V_SSCE_. Importantly, for Pt-covered Au electrodes, the first two reduction waves near +0.175 V_SSCE_ and +0.08 V_SSCE_ are no longer observed, demonstrating that adsorption and reduction of PtCl_4_^2–^_aq_ in this potential regime is a surface-selective process that proceeds on Au but not on Pt surfaces. The integrated charge for the first cycle is summarized in Table S1. For 1 mmol/L K_2_PtCl_4_ in 0.5 mol/L NaCl, the charge ranged between 0.411 mC/cm^2^ and 0.615 mC/cm^2^ depending on the background correction as indicated in Figure S3. These values bracket the nominal charge of 0.480 mC/cm^2^ expected for a monolayer of Pt on Au(111).

Voltammetry for a wider range of K_2_PtCl_4_ and NaCl concentrations is available in Figure S4, where the same transitions in behavior are observed.

### Adsorption of PtCl_4_^2**–**^ on Au

XPS was used to probe the PtCl_4_^2–^_aq_ adsorption process on Au at +0.40 V_SSCE_ enabling the coverage, chloride coordination number, and oxidation state on the derivatized electrode to be established. Exposure of piranha-cleaned^45^ Au surfaces to the K_2_PtCl_4_ containing electrolyte for 100 s results in the development of well-defined Pt 4f peaks superimposed upon the Au 5p and adjacent Au 4f features, as shown in Figure 2a. The Pt peaks persist even after the electrode is rinsed with 30 mL of deionized water (or dilute HCl with pH 3.5), indicating that the adsorbed complex is retained on the Au surface (Figure S5). By comparison, emersion and rinsing of Au at the same potential from the 0.5 mol/L NaCl supporting electrolyte alone results in the substantial loss of adsorbed halide, in contrast to the much stronger irreversible interaction between the Pt complex and Au.

**Figure 2:**
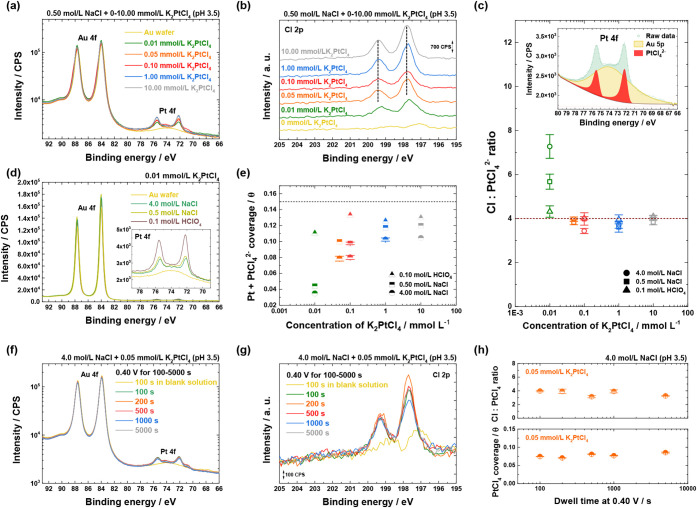
XPS characterization of PtCl_4_^2–^_ads_ adsorbed on Au at 0.40 V_SSCE_ and its dependence on Cl^–^ and K_2_PtCl_4_ concentrations. (a) Pt 4f and (b) Cl 2p spectra before and after derivatization in 0.5 mol/L NaCl at +0.40 V_SSCE_ for 100 s. (c) Cl/PtCl_4_^2–^ ratio as a function of K_2_PtCl_4_ and Cl^–^ concentration with the inset showing the peak deconvolution procedure. (d) Pt 4f spectra for PtCl_4_^2–^_ads_ adsorbed at different NaCl concentrations. (e) Fractional PtCl_4_^2–^_ads_ coverage on Au(111) as a function of K_2_PtCl_4_ and Cl^–^ concentration. (f) Pt 4f and (g) Cl 2p spectra as a function of the dwell time at 0.40 V_SSCE_. (h) Cl/PtCl_4_^2–^ ratio and coverage of PtCl_4_^2–^_ads_ for different dwell times at +0.40 V_SSCE_. Error bars in panels c and h indicate the standard deviation from three positions on a single specimen (uniformity), while in panel e from repeated experiments (reproducibility).

The Pt 4f_7/2_ spectra for the complex (72.2 eV) are distinct from elemental Pt (70.9 eV) but downshifted ≈−1.0 eV from the native K_2_PtCl_4_ salt. Likewise, the Cl 2p peak for the complex was downshifted by more than −1.5 eV compared to the K_2_PtCl_4_ salt precursor, highlighting the significant interactions between PtCl_4_^2–^ and Au. A more detailed comparison of the binding energy and associated standards is summarized in Figure S6, Figure S7 and Table S2.^48^

For PtCl_4_^2–^_ads_ derivatization experiments using K_2_PtCl_4_ concentrations greater than 0.05 mmol/L, the Pt 4f spectrum and corresponding Cl 2p spectrum (Figure 2b) reveal a sensitivity-normalized Cl/Pt intensity ratio of 4, congruent with the stoichiometry of the anionic precursor complex (Figure 2c). For more dilute 0.01 mmol/L K_2_PtCl_4_ solutions, the Cl 2p/Pt 4f ratio increases from 4.3 in *Cl* ^–^ *-free* 0.1 mol/L HClO_4_ to 7.3 in 4.0 mol/L NaCl, indicative of a mixed Cl^–^ + PtCl_4_^2–^_ads_ adlayer like that reported in two ECSTM studies.^8,28^ A downshift in the Cl 2p_3/2_ spectra from 197.8 to 197.7 eV is observed for specimens prepared in 0.01 mmol/L K_2_PtCl_4_ (Figure S7 and Figure 2b). In line with this trend, the residual Cl 2p_3/2_ peak (197.1 eV) seen in the Pt-complex-free electrolyte is clearly distinct from that of the PtCl_4_^2–^_ads_ species.

The fractional surface coverage of PtCl_4_^2–^_ads_ was derived from a simple overlayer model based on tabulated photoelectron attenuation lengths, as detailed further in the supplement (Tables S3–S5 and Figures S8 and S9).^48^ Minor elemental Pt peaks, evident for a subset of specimens, are attributed to photoreduction during the XPS measurement, as detailed in the supplement (Figure S10).^49–52^ This contribution was included in the evaluation of the PtCl_4_^2–^ coverage.

Following 100 s immersion at +0.40 V_SSCE_, the fractional PtCl_4_^2–^_ads_ coverage increases monotonically with precursor concentration to saturate at 0.12 in 0.5 mol/L NaCl or at a slightly lower value of 0.104 in 4 mol/L NaCl. Conversely, in “Cl^–^-free” 0.1 mol/L HClO_4_ (at +0.15 V_SSE_), a slightly higher saturation coverage of 0.13 is reached (Figure 2d, 2e). The dependence on free-halide concentration reflects the thermodynamics and kinetics of competitive adsorption, wherein, for Cl^–^-rich media, PtCl_4_^2–^ adsorption must await the opening of new sites via the equilibrium adsorption–desorption and surface diffusion dynamics of halide on Au. In the present work, the PtCl_4_^2–^_ads_ coverages are lower than those reported for ECSTM studies of Au(111) (≈0.15). This difference is at least partly attributed to defect-laden (111)-textured Au films with small terraces, random step orientations, and non-(111) textured areas that were used for most of our experiments.^7,8,28^ For the more dilute K_2_PtCl_4_ halide-based electrolytes, the PtCl_4_^2–^_ads_ coverage tends to fall off, reaching only 0.075 in 0.05 mmol/L K_2_PtCl_4_. Nevertheless, the independence of coverage from immersion time indicates that an equilibrium coverage has been approached by 100 s (Figure 2f). Likewise, the time stability of the Cl/Pt stoichiometry of the adsorbed complex is confirmed (Figure 2g). Accordingly, the time-independent PtCl_4_^2–^_ads_ coverage with K_2_PtCl_4_ concentration allows the trend in Figure 2e to be viewed as an adsorption isotherm for PtCl_4_^2–^_ads_ on Au during exposure at 0.40 V_SSCE_.

To further examine the derivatization process, the adsorption of PtCl_4_^2–^_aq_ on flame-annealed (111)-textured 100 nm thick Au film on mica was briefly examined by STM. The different substrate and preparation conditions result in much larger (111) terraces that ease imaging and evaluation of the adlayer. The specimen was also subjected to subsequent XPS analysis and evaluation. Following 100 s immersion at −0.4 V_SSCE_ in 0.1 mmol/L K_2_PtCl_4_ containing 0.5 mol/L NaCl, the specimen was rinsed with 18 MΩ·cm water, dried, and imaged in an Ar-flooded cell. Within a few minutes of preparation, the adsorbed PtCl_4_^2–^_ads_ complex was imaged in the form of a weakly ordered adlayer shown in Figure 3a. The most ordered regions are congruent with the $\left(\sqrt{7}x\sqrt{7}\right)R19.1^{{}^{\circ}}$ structure previously reported for PtCl_4_^2–^_ads_ adsorption on large Au(111) single crystal terraces.^7,8,28^ However, the adlayer is highly defective, which is partly due to the limited terrace size and the steric impact of the meandering steps. Comparison of sequential images reveals significant structural fluctuations associated with the high defect density that may also be subject to perturbation by the STM probe tip. Nevertheless, the extent of adlayer ordering across the specimen may reflect the larger terraces on the flame-annealed Au film on mica as compared to the PVD Au films used in the more extensive XPS experiments, e.g., Figure 2e.

**Figure 3:**
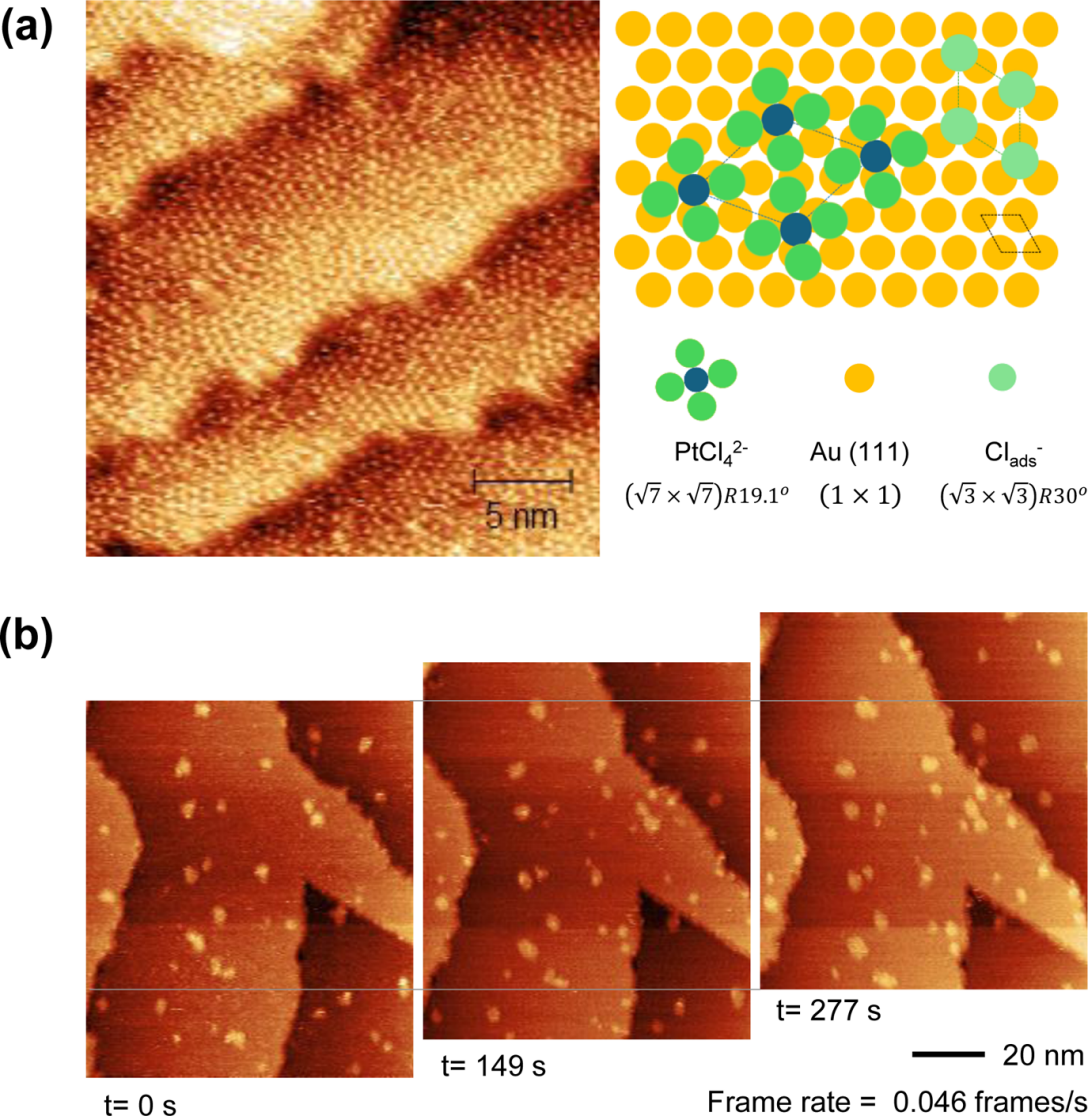
(a) STM image of PtCl_4_^2–^_ads_ on a flame-annealed stepped Au(111) film on mica, along with a schematic image of the $\left(\sqrt{7}\times \sqrt{7}\right)R19.1^{{}^{\circ}}$ PtCl_4_^2–^_ads_ adlayer. Image was collected at 0.45 nA with a +0.1 V sample bias in an Ar environment. (b) A selection of three images showing the evolution of Pt islands and step-edge decoration following the reduction of PtCl_4_^2–^_ads_ adlayer. Imaging was performed at 0.45 nA with a +0.1 V sample bias.

Interestingly, following a tip crash and exchange, continued imaging over a wider area revealed conversion of the adlayer to the metallic phase. The Pt atoms from the reduced precursor quickly coalesced to form two-dimensional Pt islands or were captured at the lower step edges, as shown in Figure 3b. The respective regions continue to grow slightly over the next few minutes, while additional islands form near the upper side of the steps. Fluctuations along the perimeter, and occasionally on top of the islands, are likely associated with mobile Au adatoms that segregate to lower the surface energy. For a surface covered with a compact $\left(\sqrt{7}\times \sqrt{7}\right)R19.1^{{}^{\circ}}$ adlayer structure, the fractional Pt surface coverage is 0.14. An estimate of the Pt coverage in the final Figure 3b image, including both the islands and covered step edges, yields an upper bound coverage of 12%. Following STM examination, the specimen was analyzed by XPS at six different locations, and all the Pt was in the metallic form, with surface coverage determined to be 0.10 ± 0.002, with a negligible Cl 2p signal (Figure S11). In this experiment, it is not entirely clear how Cl^–^ associated with the PtCl_4_^2–^_ads_ was desorbed from the specimen, but radiolytic interactions between the remaining Cl^–^ and adventitous C and O may have contributed to its removal. With extended imaging, interesting “finger-like” structures develop at the step edges, as shown in Figure S12.

Comparative experiments were conducted using a Pt­(IV) precursor. As detailed in the Supporting Information (Figures S13 and S14), PtCl_6_^2–^ undergoes an initial 2e^–^ reduction to form the same PtCl_4_^2–^_ads_ intermediate at +0.40 V_SSCE_, yielding nearly identical XPS spectra and adsorption isotherms. This aligns well with previous thin-layer electrochemical cell measurements in Pt­(IV)/Pt­(II) halide electrolytes.^43,44^ Next, different strategies to reduce the surface-selective adsorbed PtCl_4_^2–^_ads_ intermediate to Pt are explored; to simplify matters, a Pt­(II) precursor is used in all subsequent experiments.

### Two-Step Process: PtCl_4_^2**–**^ Adsorption and Reduction in Different Electrolytes

With adsorption of PtCl_4_^2–^ on freshly prepared Au electrodes emersed at 0.4 V_SSCE_ confirmed by XPS, the reduction waves observed in Figure 1 mark the reduction of the adsorbate to the metallic form. The absence of the first two waves on a Pt-covered surface reflects the surface-selective nature of the process, distinguishing the initial two-dimensional growth from subsequent bulk deposition. To further isolate PtCl_4_^2–^ adsorption from its subsequent reduction to Pt, a two-step process was employed, similar to that previously used in ECSTM studies.^7,8,28^ Derivatization was carried out in 0.01 to 10.00 mmol/L K_2_PtCl_4_ dissolved in 0.1 mol/L HClO_4_, followed by transfer to 4 mol/L NaCl for the reduction step. To minimize spontaneous ligand exchange between Cl^–^ and H_2_O derivatization was performed shortly after dissolving the Pt salt (typically within 16 min). Details regarding the time-dependent stability and results for the various electrolytes are provided in the Supporting Information (Figures S15–S17).

Voltammetric reduction of the saturated coverage PtCl_4_^2–^ adlayer in 4 mol/L NaCl proceeds via two waves, as shown in Figure 4. These two reduction peaks (0.166 V_SSCE_ and 0.07 V_SSCE_) are observed at more positive potentials than those seen in the fully comprised 4 mol/L NaCl electrolyte containing 0.10 to 10 mmol/L K_2_PtCl_4_ (0.146 V_SSCE_ and 0.04 V_SSCE_, Figure S4), which reflects the large additional contribution from the reduction of PtCl_4_^2–^_aq_.

**Figure 4:**
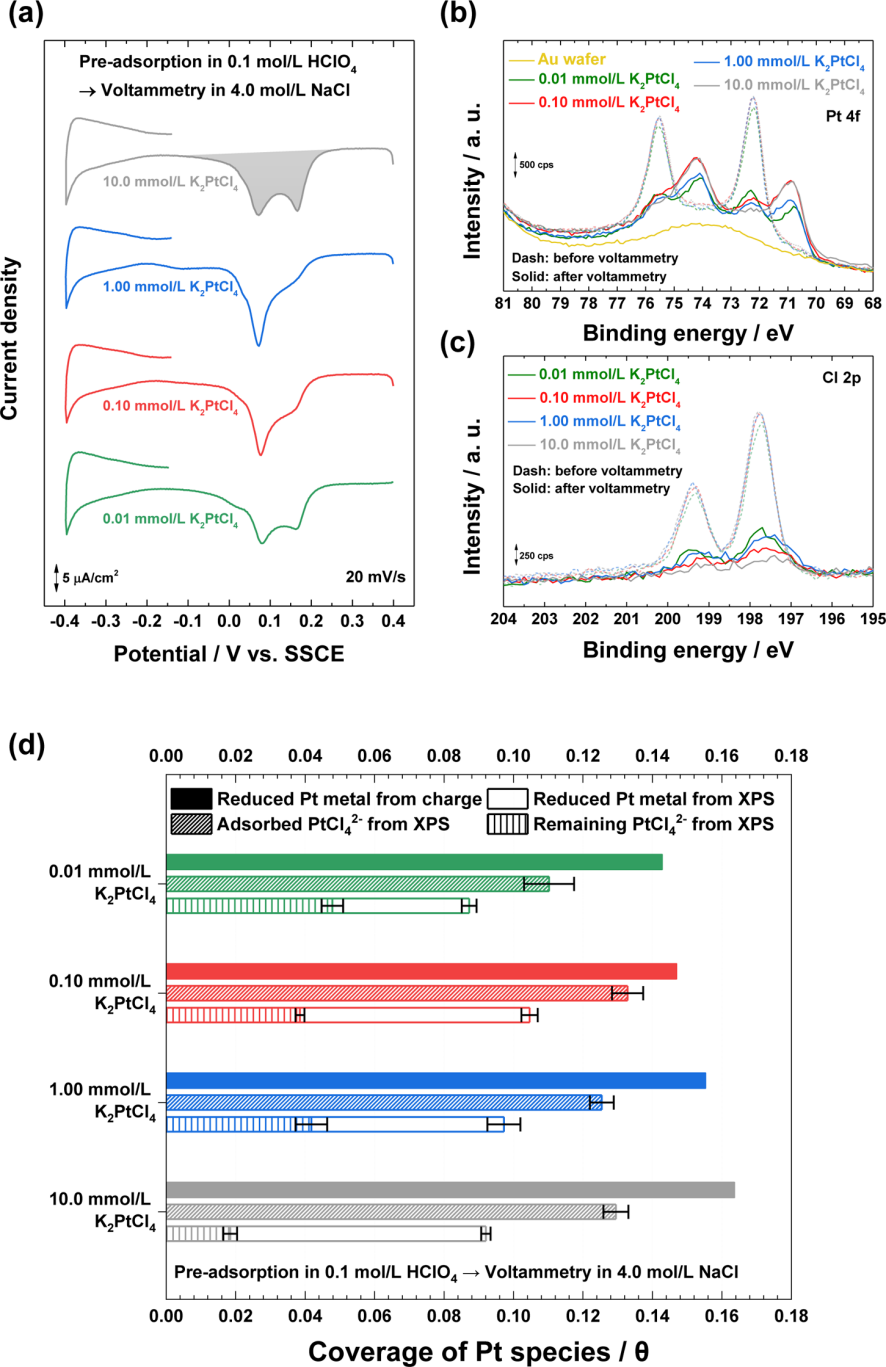
Two-step Pt deposition process where derivatization of PtCl_4_^2–^_ads_ on Au is followed by electrode transfer and reduction in a Pt-free supporting electrolyte. (a) Voltammetric reduction of PtCl_4_^2–^_ads_ on Au in 4.0 mol/L NaCl after derivatization at +0.15 V_SSE_ for 100 s in freshly prepared (0.01 to 10.00) mmol/L K_2_PtCl_4_ in 0.1 mol/L HClO_4_. (b, c) Pt 4f and Cl 2p spectra after PtCl_4_^2–^_ads_ derivatization on Au in 0.1 mol/L HClO_4_ (dash) and following its reduction to Pt (solid) in Pt-free 4.0 mol/L NaCl. (d) Mass balance reveals the efficiency of PtCl_4_^2–^_ads_ reduction to Pt in Pt-free 4.0 mol/L NaCl. Error bars indicate the standard deviation for measurements taken from three positions on a single specimen (uniformity).

XPS analysis following the potentiodynamic reduction scan in 4 mol/L NaCl confirms the transition to metallic Pt, as indicated by the Pt 4f peak shift and decreased Cl 2p intensity (Figure 4b and 4c). The presence of residual oxidized Pt­(II) features (Figure S17) is attributed to the oxidation of highly reactive Pt islands/atoms during emersion. The reduced Pt fraction determined by XPS is slightly lower than the initial coverage (Figure 4d), indicating up to 20% loss, or variation, in the adsorbed complexes; nevertheless, the results are congruent with reduction of the majority of the adsorbed PtCl_4_^2–^_ads_ precursor to metallic Pt. At the same time, the persistent excess reduction charge is attributed to either unaccounted capacitance or parasitic O_2_ reduction.

### Self-Terminating Potentiodynamic Deposition of a Pt Monolayer

The surface-selective nature of the two-dimensional Pt growth observed in Figure 1c originates from the fundamental difference in the potential-dependent halide adsorption dynamics on Au and Pt whereby for a meaningful potential range, PtCl_4_^2–^_ads_ adsorption is inhibited on Pt-covered surfaces while remaining active on Au. For Pt(111), a higher coverage (0.44) (3 × 3) Cl^–^ adlayer forms with a strong binding energy of −79 meV/Å^2^.^47^ In contrast, *in situ* surface X-ray scattering indicates that ordered incommensurate Cl^–^ adlayers on Au only form at coverages in excess of 0.5, which are reached at more positive potentials, above +0.675 V_Ag/AgCl/3MKCl_ in 0.1 mol/L NaCl + 0.1 mol/L HClO_4_.^53^ On the other hand, an ultrahigh-vacuum scanning tunneling microscope study revealed the formation of an ordered Cl^–^ adlayer with a √3 × √3 R30° structure at 0.33 ML coverage, with an adsorption energy of −42 meV/Å^2^ that is about half that of Cl^–^ saturated Pt.^54^ ECSTM measurements of Au(111) in 10 mmol/L HCl in 0.1 mol/L HClO_4_ reveal at least two higher-order phases, a √19 × √13 phase at 0.38 V_RHE_ and a 5√3 × 3√7 phase at 0.77 V_RHE_, where the coverage increases with potential. The imaged phases presumably correspond to the compressible halide adlayer previously identified by SXRD.^53,55^ Accordingly, at +0.40 V_SSCE_, the Au surface is covered by a subsaturation Cl^–^ adlayer.^53,54^ Unlike the dense Cl^–^ adlayer on Pt, the halide layer on Au remains dynamic enough for PtCl_4_^2–^_aq_ to compete for surface sites. It is this difference in adsorption behavior that underlies the self-limiting deposition of Pt monolayers on Au.

Based on competitive adsorption between PtCl_4_^2–^ and Cl^–^, a protocol was developed to achieve surface-selective Pt monolayer deposition within a single electrochemical cell. This was accomplished by targeting the potential regime where PtCl_4_^2–^ and Cl^–^ compete for Au or Pt surface sites, combined with potential modulation to control Pt nucleation and growth within the same electrolyte (Figure 5). For example, surface-selective potentiodynamic Pt deposition is demonstrated by adsorbing PtCl_4_^2–^_aq_, at 0.40 V_SSCE_ for 100 s and then sweeping the potential to 0 V_SSCE_ in (0.01 to 10) mmol/L K_2_PtCl_4_ in 4.0 mol/L NaCl solutions (Figure 5a).

**Figure 5:**
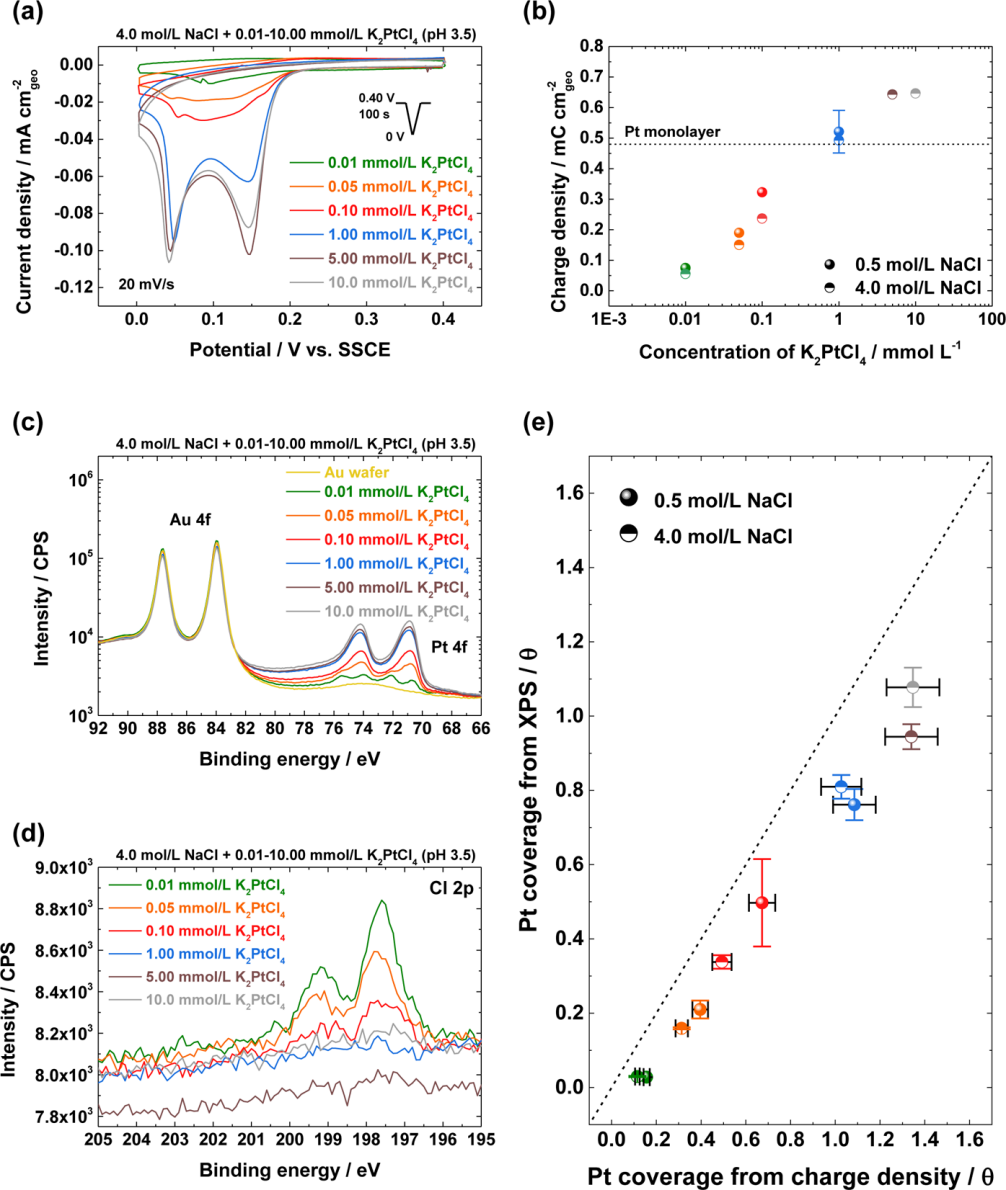
Potentiodynamic Pt deposition in different K_2_PtCl_4_–NaCl electrolytes. (a) Voltammetry initiated after 100 s hold at +0.40 V_SSCE_ in (0.01–10.0) mmol/L K_2_PtCl_4_ in 4.0 mol/L NaCl. (b) Net voltammetric reduction charge in (a). (c, d) Pt 4f and Cl 2p spectra following one voltammetric deposition cycle. (e) Correlation between Pt coverage derived from XPS and the voltammetric reduction charge. Error bars: (b) from repeated experiments (reproducibility), (e, *y*-axis) from three positions (specimen uniformity), and (e, *x*-axis) from area measurement uncertainty (≈8.75%).

Two waves for site-selective deposition are readily evident in Figure 5a particularly for higher K_2_PtCl_4_ concentrations. The initial portion of the first wave is associated with the reduction of PtCl_4_^2–^_ads_ that was adsorbed at 0.40 V_SSCE_. This is followed by the second wave that reflects kinetic hindrance from the competitive exchange dynamics between PtCl_4_^2–^_aq_ and halide-covered Au surface segments, along with the first-order reaction kinetics associated with the reduction of PtCl_4_^2–^_aq_ species.

For concentrations below 1.0 mmol/L, K_2_PtCl_4_ pretreatment at +0.40 V_SSCE_ does not saturate the surface with PtCl_4_^2–^_ads_. Rather, the initial contribution reflects the isotherm coverage established in Figure 2e. For lower bulk K_2_PtCl_4_ concentrations, the initial contributions from of PtCl_4_^2–^_ads_ are diminished further, and the rate of direct reduction of solvated PtCl_4_^2–^_aq_ is also limited. For the most dilute 0.01 mmol/L K_2_PtCl_4_ electrolyte examined, the modest PtCl_4_^2–^_ads_ coverage and limited contributions from PtCl_4_^2–^_aq_ reduction enable the Cl^–^ adsorption wave associated with the available Au sites to be seen at +0.158 V_SSCE_ (Figure S18a and S18b). For higher K_2_PtCl_4_ concentrations, an increase in net reductive currents emerges during the return sweep, and the Cl^–^ adsorption peak shifts to more positive values (+0.225 V_SSCE_ at 0.10 mmol/L), reflecting the increased competition between PtCl_4_^2–^_aq_ and Cl^–^_aq_ for the diminished number of Au surface sites (Figure S18b).

For K_2_PtCl_4_ ≥ 1 mmol/L, the voltammetric reduction waves in Figure 5a saturate reflecting the loss of available Au surface sites for further adsorption of PtCl_4_^2–^_aq_ and eventually termination of further Pt deposition. For the reduction of PtCl_4_^2–^ to Pt^0^ (Figure 5b), a monolayer equivalent charge of 0.480 mC/cm^2^ is anticipated. For higher K_2_PtCl_4_ concentrations, the net charge for one cycle saturates near 0.65 mC/cm^2^ for K_2_PtCl_4_ ≥ 5 mmol/L. Recall that in addition to Pt deposition, the charge also includes capacitive contributions for the evolving surface.

XPS analysis (Figure 5c) confirms the above trends. As the K_2_PtCl_4_ concentration increases, the Pt 4f spectra show an increase in reduced Pt metal alongside a decrease in PtCl_4_^2–^_ads_ remaining on Au sites after the return scan to +0.40 V_SSCE._ The magnitude of the corresponding Cl 2p spectra (Figure 5d) tracks with the PtCl_4_^2–^_ads_ component of the Pt 4f spectra (Figure 5c) following completion of the scan at +0.4 V_SSCE_.

For more concentrated K_2_PtCl_4_ electrolytes, the resulting Pt 4f spectra saturate as the coverage approaches monolayer dimensions while the Cl 2p decreases to background levels, consistent with the absence of PtCl_4_^2–^_ads_ from the Pt-covered surface. Comparison between the coulometric and XPS-determined Pt thickness is summarized in Figure 5e. The excess charge is likely due to contributions from capacitive and adsorbate-based pseudocapacitive charging during the 20 mV/s potentiodynamic scan. The same experimental protocol was applied using the less concentrated 0.5 mol/L NaCl supporting electrolyte, and similar results were obtained (Figure S19).

Nucleation and growth of Pt from the reduction of adsorbed PtCl_4_^2–^_ads_ are expected to be a sensitive functions of the potential program. The same applies to deposition from PtCl_4_^2–^_aq_, with potential-sensitive, site-dependent behavior expected for both reactions. Microstructural evolution associated with the reduction of adsorbed PtCl_4_^2–^_ads_ can be isolated by using fast voltammetry to minimize contributions from the reduction of fully solvated PtCl_4_^2–^_aq_ in 0.1 mmol/L K_2_PtCl_4_ and 0.5 mol/L NaCl. For example, after flame annealing a (111)-textured Au thin film on mica, it was immersed for 100 s at 0.4 V_SSCE_ followed by a 1 V/s scan to 0 V_SSCE_ and a return scan to 0.4 V_SSCE_ for a 100 s hold. This was followed by relaxation under open-circuit conditions and a brief water rinse during emersion. STM examination revealed a modest coverage of Pt monolayer islands with an average diameter ∼2.2 nm across the central terrace region, as shown in Figure 6. The Au steps are decorated with Pt islands that are two monolayers thick, while the adjacent Au steps are clearly eroded with monolayer-deep pits. The two-layer structures are attributed to Au segregation on top of the freshly formed Pt monolayer-thick islands. An upper bound image analysis of the fractional surface coverage of Pt islands and step decoration is congruent with the reduction of the PtCl_4_^2–^_ads_ precursor, approaching 0.15, while XPS measurements yield a coverage of 0.1. The dispersion is at least partly attributed to the effect of Au segregation on top of the Pt islands, the limited area analyzed, and the algorithmic choices used to evaluate the surface coverage. The periodic modulation of electron density on the terrace might be related to PtCl_4_^2–^_ads_ and/or Cl^–^ formed during the return step to +0.4 V_SSCE_ or alternatively, may reflect some adventitious species from or following emersion. As indicated in Figure 3a, atomically resolved imaging is very sensitive to tip state. Further still, the deposition and rearrangement of Pt at the step edges is distinctly different from that seen in Figure 3b and speaks to the complexity of the deposition process and the need for further *in situ* study.

**Figure 6:**
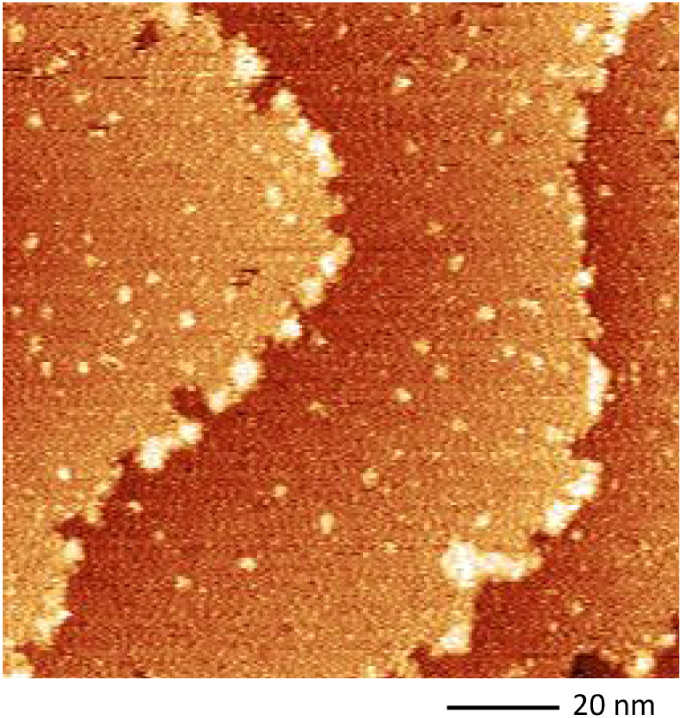
Pt deposition on a flame-annealed (111)-textured Au thin film supported on mica. After an initial 100 s hold at 0.4 V_SSCE_, the potential was scanned to 0 V_SSCE_ (1 V/s) and back to 0.4 V_SSCE_, followed by a subsequent 100 s hold. The ex situ image was collected in an Ar environment with a tunneling current of 1.36 nA and a specimen bias of −0.1 V.

Going further, the impact of multiple cycles of voltammetric deposition in 0.5 mol/L NaCl containing 0.10 mmol/L K_2_PtCl_4_ was examined by coulometry and XPS. Following the first cycle, a reduction wave is still evident, although the charge became much smaller and the peak shape substantially less distinct with cycling (Figure 7a). Initially, the net charge builds rapidly but asymptotes by the sixth cycle to a fixed incremental contribution attributed to background capacitive charging (Figure 7b). XPS analysis following one cycle yields a coverage ≈0.50 ± 0.12, followed by saturation at a monolayer after 5 cycles (Figure 7b and Figure S20). However, performing the same experiment in 1.00 mM K_2_PtCl_4_ revealed that the net charge does not approach background levels until ≥20 cycles. Congruent with this, XPS shows that the film thickness reaches a monolayer after 3 cycles while asymptotically approaching ≈1.7 monolayers by 20 cycles (Figure 7c and Figure S21).

**Figure 7:**
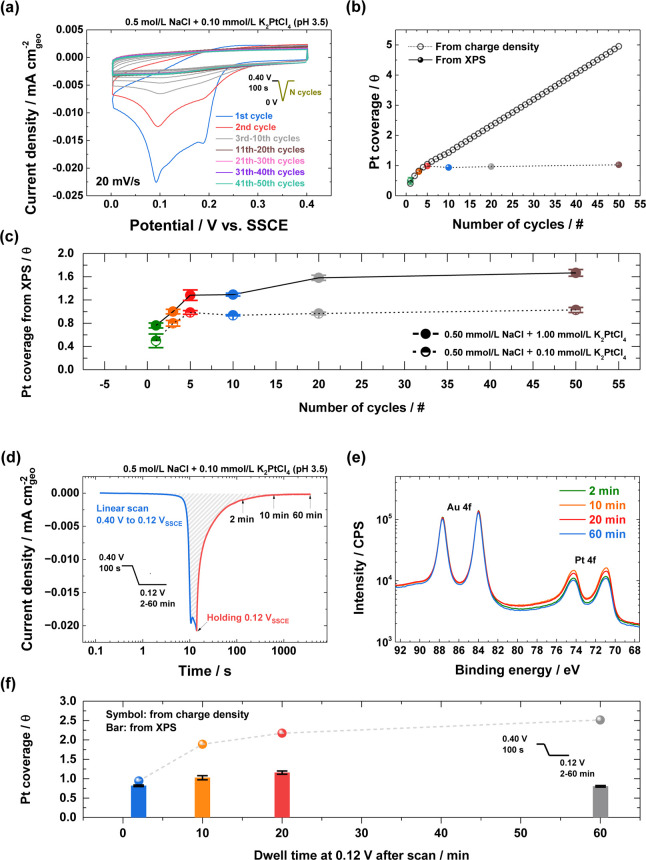
(a) Fifty consecutive voltammograms initiated after a 100 s hold at +0.40 V_SSCE_ in 0.5 mol/L NaCl containing 0.10 mol/L K_2_PtCl_4_. (b) Net accumulated fractional Pt coverage after specified number of voltammetric cycles in 0.5 mol/L NaCl containing 0.10 mol/L K_2_PtCl_4_. (c) Net accumulated fractional Pt coverage after specified number of voltammetric cycles in 0.5 mol/L NaCl containing either 0.10 or 1.00 mol/L K_2_PtCl_4_. (d) Using a different approach, voltammetry was combined with potentiostatic deposition to grow Pt films on Au in 0.5 mol/L NaCl containing 0.10 mol/L K_2_PtCl_4_. The electrode was derivatized at 0.40 V_SSCE_ for 100 s, then swept to 0.12 V_SSCE_, and held for different times. (e) Pt 4f spectra after the specified time at 0.12 V_SSCE_ (f) Pt coverage as a function of time at 0.12 V_SSCE_. Error bars in panels c and f indicate the standard deviation of results from three positions (uniformity).

As indicated by RSXRS^15^ Pt deposition is strongly potential-dependent, ranging from terminating prematurely at small overpotentials to proceeding into a multilayer regime before transitioning to three-dimensional growth at greater overpotentials. A related dependence on the K_2_PtCl_4_ concentration might be anticipated. Voltammetry in 10 mmol/L K_2_PtCl_4_ reveals a postsurface wave current minima near +0.05 V_SSCE_ (Figure 1c and Figure S4) that marks the transition to 3D growth, while for more dilute solutions the corresponding uptick is delayed until more negative potentials.

Growth beyond the monolayer limit likely arises from a combination of substrate-mediated effects, such as surface segregation and/or surface alloying with Au. The former is driven by the lower surface tension of Au, while intermixing is easily facilitated by the segregation of Au at steps of the growing Pt islands. Idling at 0.40 V_SSCE_ provides an extended period for these dynamics to be expressed.^56–58^ Furthermore, recent calculations indicate that it is energetically viable for significant Pt dewetting to occur.^59^ Taken in combination the above effects will provide additional Au sites for selective adsorption and reduction of PtCl_4_^2–^ such that deposition may proceed beyond a single monolayer. That said, one may surmise that as the surface becomes increasingly Pt-rich, pathways for Au surface segregation become narrower, and the process will slow down, congruent with the observed asymptotic behavior.

The two-dimensional growth process was further examined using potentiostatic conditions, where film growth and the cumulative effects from both preadsorbed and solvated species are followed at a fixed driving force. In this particular variation, the electrode is swept to 0.12 V_SSCE_ that overlaps with the second voltammetric surface wave in Figure 1, and held for up to 60 min, to track the time-dependent contributions from the reduction of preadsorbed PtCl_4_^2–^_ads_ and solvated PtCl_4_^2–^_aq_ (Figure 7d and Figure S22). With the reduction of the preadsorbed PtCl_4_^2–^_ads_ underway, additional Au surface sites become available for the adsorption and reduction of PtCl_4_^2–^_aq_. The combination of transport constraints and charge transfer kinetics, along with the continuous decrease in available reaction sites, causes the current density to decay sharply (Figure 7d). The total charge approaches monolayer coverage within approximately 3 min while the remaining background current is most likely associated with oxygen reduction. Congruent with this, XPS reveals Pt coverage of 0.82 monolayers at 2 min, with saturation near a monolayer evident at longer dwell times (Figure 7e and f).

### Pulse Deposition of Pt Monolayer Films Approaching the Two-Step ALD Limit

Beyond self-limiting monolayer formation, decoupling surface-selective PtCl_4_^2–^ adsorption from its reduction offers the prospect of tuning the Pt coverage with exquisite accuracy. This can be realized by a repetitive two-step transfer between electrolytes, as detailed above, or by using a single electrolytic bath combined with an asymmetric potential pulse duty cycle optimized for the given K_2_PtCl_4_ concentration.

Using the latter approach, the current transients in 0.10 mmol/L K_2_PtCl_4_ containing 0.5 mol/L NaCl electrolyte (Figure 8a) allow the growth process to be monitored closely. A robust reduction current at 0.10 V_SSCE_ marks the start of the pulsing sequence as the PtCl_4_^2–^_ads_ adlayer on the pristine Au surface is converted to Pt metal. As the cycle number increases, the peak reduction current decreases, reflecting the progressive loss of available Au sites for PtCl_4_^2–^ adsorption. The current transients eventually stabilize as the surface approaches saturation Pt coverage. Consistent with this, the Pt 4f and Au 4f spectra for the deposited films grown reveal saturation beyond 30 pulse cycles.

**Figure 8:**
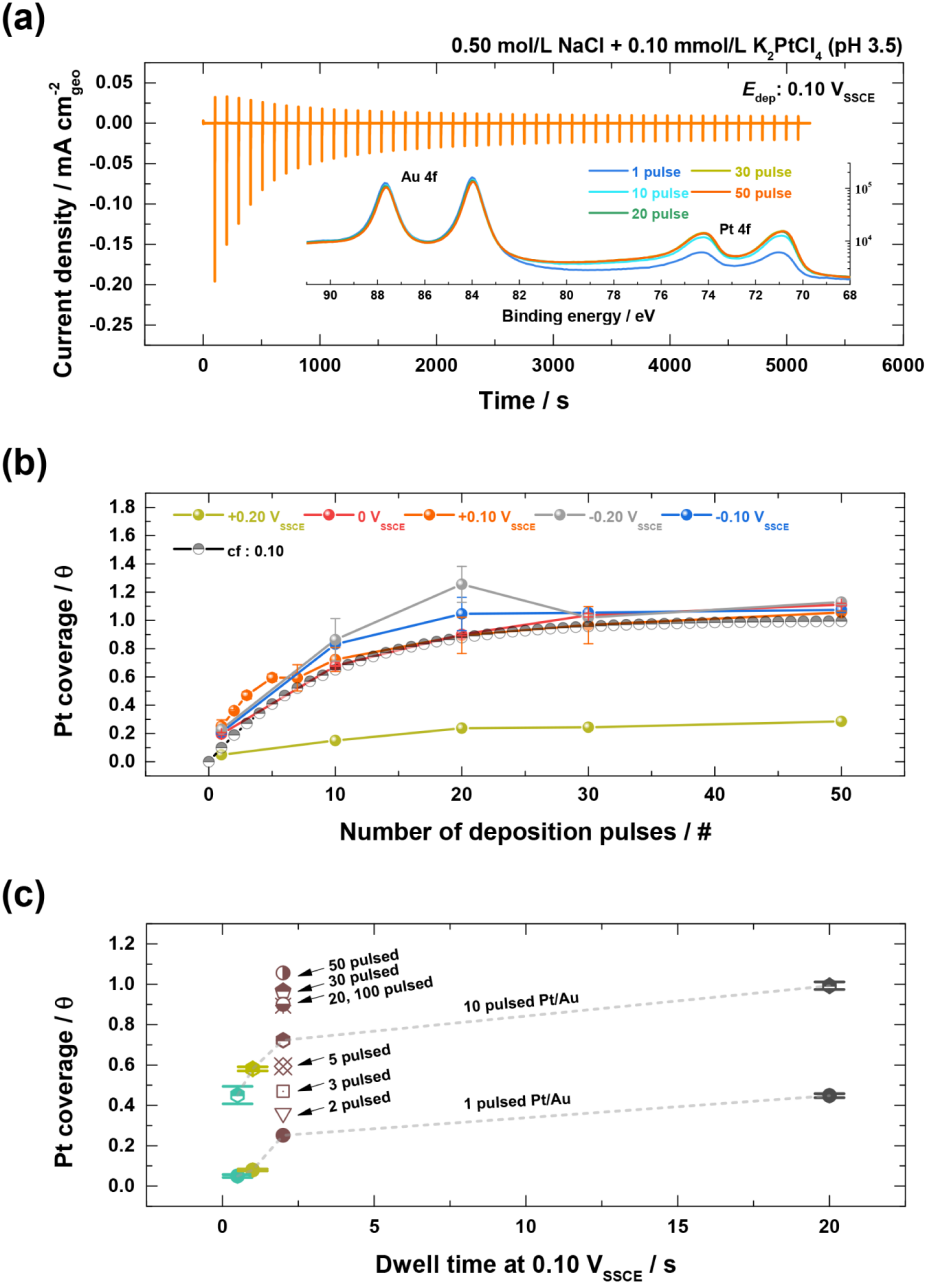
(a) Current transient for the deposition of a 50-pulse Pt film from 0.5 mol/L NaCl containing 0.10 mol/L K_2_PtCl_4_. Inset: A selection of Pt 4f-Au 4f spectra for n-pulse Pt/Au films. (b) Pt coverage after 2 s at different *E*_dep_ reduction potentials. The cf curve corresponds to eq 1 for $\theta_{PtCl4ads}^{sat}$ = 0.1. (c) Impact of dwell time at *E*_dep_ of 0.10 V_SSCE_. Error bars in panel b are from repeated experiments (reproducibility), while those in panel c indicate the standard deviation of results from three positions on a single specimen (uniformity).

The impact of deposition potential, *E*_dep_, between +0.20 and −0.20 V_SSCE_ (Figure 8b) and dwell time from 0.5 to 20 s (Figure 8c and Figure S23) on Pt deposition was examined. For *E*_dep_ of +0.20 V_SSCE_, Pt coverage remains below 0.3 monolayers even after 50 pulses due to kinetic limitations on the rate of PtCl_4_^2–^_ads_ reduction, congruent with the voltammetry shown in Figure S4 and Figure 7a. In contrast, for all other *E*_dep_ conditions, saturation near one monolayer is attained after 20 to 30 pulses, demonstrating that PtCl_4_^2–^_ads_ is effectively reduced for potentials at, or below, 0.10 V_SSCE_.

For longer dwell times (*E*_dep_ ≥ 2 s), the coverage clearly exceeds that associated with preadsorbed PtCl_4_^2–^_ads_, with the additional contributions coming from the reduction of fully solvated PtCl_4_^2–^_aq_ on freshly available Au surface regions. Extending the process to 100 cycles or expanding the time at *E*_dep_ to 20 s does not lead to growth beyond monolayer dimensions. The saturation behavior is further confirmed by H_upd_ voltammetry (Figure S24). The 30- and 50-pulsed samples exhibit nearly identical voltammetric features, with the H_upd_ desorption charge yielding a Pt coverage of approximately 0.975 ML, consistent with the formation of a nearly complete monolayer.

The asymmetric pulse potential deposition process further underscore the central role of surface-selective PtCl_4_^2–^ adsorption on Au sites in limiting Pt deposition. In another limit, holding *E*_dep_ for an extended period is tantamount to potentiostatic deposition, with growth terminating near a monolayer thickness. Conversely, for shorter durations <2 s pulses to *E*_dep_ (0.10 V_SSCE_), the Pt coverage falls off noticeably for a given number of cycles (Figure 8c, Figure S23) due to limitations related to the kinetics of PtCl_4_^2–^_aq_ reduction. While various K_2_PtCl_4_ concentrations were examined (see Figures S25), the 0.10 mmol/L condition was selected for further analysis, as self-limiting deposition is restricted to a monolayer.

### Geometric Constraint on Two-Dimensional Island Growth and Coalescence

Under optimized pulsing conditions, film growth begins with surface-selective adsorption of PtCl_4_^2–^. The specific duration of each potential pulse influences the mesoscale structural evolution of the film based on the nucleation and growth of Pt islands from the reduction of the adsorbed precursor and subsequent contributions from the reduction of the solvated precursor. Regardless of the relative contributions, the essence of self-limiting growth lies in the site selectivity of the precursor.

At +0.40 V_SSCE_, the surface-selective process is governed by the relative adsorption strength of the anions present, PtCl_4_^2–^_aq_ and Cl^–^_aq_. On Au, the adsorbed Cl^–^ layer is below a saturation coverage and weakly bound, allowing the PtCl_4_^2–^_aq_ precursor to effectively compete for and displace the halide due to its higher adsorption energy.^47,53–55^ In contrast, the Pt surface is protected by a dense, saturated Cl^–^ adlayer that acts as a physical barrier to the adsorption of PtCl_4_^2–^_aq_.^60,61^ During the charge transfer reduction step, the Cl^–^ ligands are released, and the resulting Pt adatoms agglomerate into islands that are covered by adsorbed halide. The consolidation of Pt into islands opens up fresh Au sites and relaxes the steric constraint on further PtCl_4_^2–^_aq_ adsorption during subsequent cycles. This growth sequence proceeds until the incipient coalescence of the Pt islands completely shields the Au surface, leading to the observed self-limiting behavior. Importantly, the final step of island coalescence may be hindered due to a finite size effect, with PtCl_4_^2–^_aq_ being unable to adsorb in the flat conformation within the small Au surface gap between the impinging Pt islands.^39^ This would account for the common observation of incomplete coealesence of Pt monolayer films deposited from halide complexes at room temperature.^6,7,14,16^

Area conservation during two-dimensional growth is such that the two-step process can be described by the following expression

1 $$\theta_{Pt}^{i+1}=\theta_{Pt}^{i}+\theta_{PtCl4ads}^{sat}\left(1‐\theta_{Pt}^{i}\right)$$

where $\theta_{PtCl4ads}^{sat}$ for a given electrolyte corresponds to the saturated fractional coverage on Au surface segments as established by immersion at 0.40 V_SSCE_ for 100 s detailed in Figure 2e, e.g., $\theta_{PtCl4ads}^{sat}$ = 0.1 on Au for 0.5 mol/L NaCl containing 0.10 mmol/L K_2_PtCl_4_. As evident in Figure 8b, the simple model gives favorable agreement with the experiment at and beyond 10 pulse cycles.

Inspection reveals that the coverage of 0.2 to 0.25 after the first pulse is more than double the 0.1 available from preadsorbed PtCl_4_^2–^_ads_. This is attributed to an additional increment of deposition from solvated PtCl_4_^2–^_aq_. As the available Au surface sites fill in, further growth is limited by the PtCl_4_^2–^_ads_ precursor coverage, as indicated in eq 1. Inspection of Figure 8b and Figure 8c indicate that the unwanted additional contribution from deposition via PtCl_4_^2–^_aq_ can be effectively minimized by limiting dwell times to <2 s (for *E*_dep_ between 0 V_SSCE_ and +0.10 V_SSCE_). For these more optimal conditions, the coverage after 10 pulses is 0.58 for a 1 s dwell at +0.10 V_SSCE_ in good agreement with the expected value for growth solely from preadsorbed PtCl_4_^2–^_ads_.

The mesoscale structure of Pt deposited for different times at *E*_dep_ was briefly examined. Following a 2 s pulse to 0.1 V_SSCE_, several features are readily evident in Figure 9 where the original flame-annealed Au surface is provided for reference. Pt deposition is associated with at least five distinct microstructural features: (i) a small coverage of monolayer-thick islands on the Au(111) terraces that are likely the results of the reduction of the adsorbed precursor PtCl_4_^2–^_ads_, (ii) a high density of Pt clusters at Au steps that have a significant kink density, i.e., the non-(110)-oriented steps, (iii) an absence of Pt deposition at (110)-faceted steps, (iv) adjacent to the Pt-covered step edges are monolayer-deep pits or vacancy clusters that indicate removal and either incorporation or segregation of Au within or on top of the Pt islands, and (v) a significant number of two-monolayer-thick Pt islands are present on the terraces that, in several instances, are clustered in a donut-like arrangement, the center of which, in a subset of cases, may correspond to a monolayer deep pit in the substrate.

**Figure 9:**
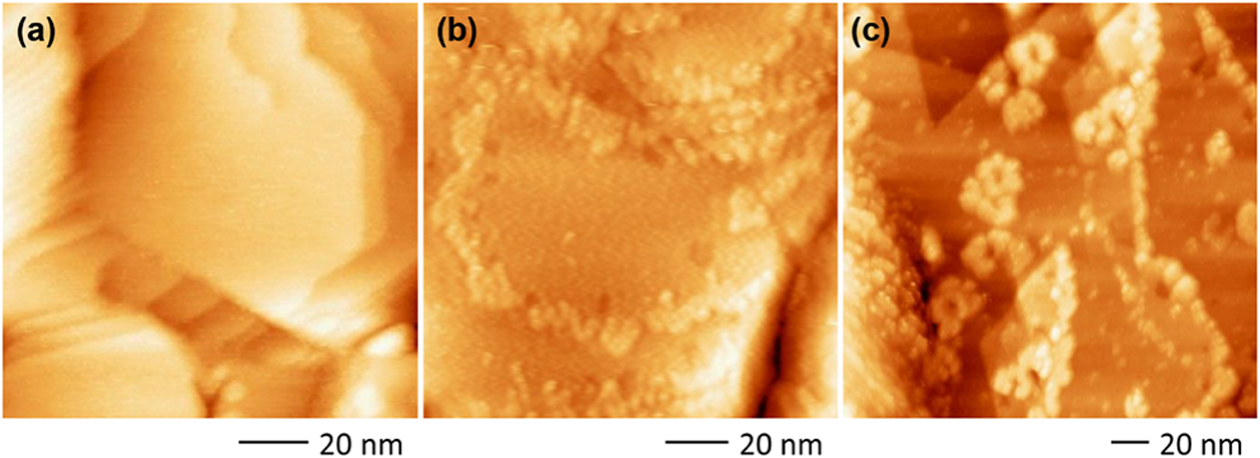
Representative STM images: (a) a flame-annealed Au film on mica. (b, c) Pt electrodeposition on flame-annealed Au films by a pulsed potential process; 0.4 V_SSCE_ for 100 s, 0.1 V_SSCE_ for 2 s, and 0.40 V_SSCE_ for 100 s in 0.1 mmol/L K_2_PtCl_4_ and 0.5 mol/L NaCl. Nucleation and growth of Pt occur preferentially at curved steps, accompanied by vacancy cluster development in the underlying substrate due to Au surface segregation on the Pt islands.

Comparison with the earlier results in Figures 3 and 6 reveals that, in addition to the limited population of Pt monolayer islands on the terraces, there exist larger structures that are associated with the reduction from PtCl_4_^2–^_aq_. Likewise, it is clear from the images shown and multiple surface sections (not shown) that deposition from the PtCl_4_^2–^_aq_ species is strongly biased toward steps. This aligns with earlier observations of overpotential Pt deposition on Au(111) from 1 mmol/L K_2_PtCl_4_ in 0.1 mol/L H_2_SO_4_ as well as a more recent study of increased uniformity of Pt monolayer shells observed on high step density, spherical Au nanoparticles.^7,62^ The Pt coverage determined by XPS is 0.15 (Figure S26) in reasonable agreement with the Pt islands and the buildup of Pt at the steps evident in the STM images, Figure 9b,c, respectively. The favorable coverage comparison supports the interpretation that the two-monolayer-thick island structures arise from Au segregation on top of Pt islands. Significantly, very similar two-layer high islands and substrate pits have been reported for physical vapor deposition of Pt and Pd on Au(111) and Ag(111) surfaces at room temperature.^63–65^ Pt island nucleation on terraces was attributed to a two-step process in which Pt atoms first exchange with Ag atoms on the (111)-terraces to form nucleation sites that are followed by the growth of 2D Pt that is subsequently capped by substrate atoms to lower the surface tension.^63^ Similar dynamics are evident in the present system, although the contribution, rate, and sequence of the different surface processes must await an *in situ* study.

Exploration of the upper coverage limit following 20 min of potentiostatic deposition at *E*_dep_ reveals a surface saturated by Pt islands. Comparison with the original flame-annealed Au surface serves as a useful reference for justifying the assignments in Figure 10. The Pt islands have a diameter similar to the more limited set of islands seen after only one cycle of 2 s depositon at *E*_dep_ (Figure 9b, c). The similarity and uniformity of the island size between Figure 9b,c and Figure 10b,c indicate that the morphology is not the result of instantaneous nucleation and a fixed growth rate, but rather some other process operates that limit the island dimensions. As noted earlier, the failure to reach complete film coalescence of the overlayer is likely related to the finite size of the PtCl_4_^2–^_aq_ complex and its inability to adsorb at the narrow junctions between the islands. XPS analysis indicates a Pt coverage of 1.9 monolayers (Figure S27). This is thicker than the monolayer coverage of Pt films grown on piranha-cleaned (111)-textured PVD Au substrates under nominally identical conditions. The origin of this discrepancy is the subject of ongoing research.

**Figure 10:**
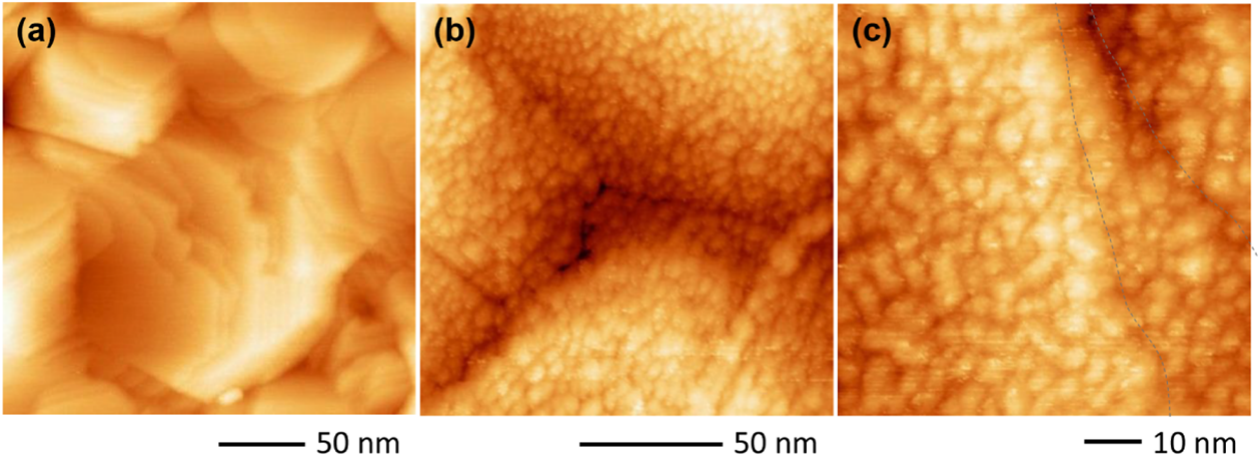
Representative STM images of (a) a flame annealed Au film on mica. (b, c) Pt electrodeposition on flame-annealed Au films by a pulsed potential process; 0.4 V_SSCE_ for 100 s, 0.1 V_SSCE_ for 1200 s, and 0.40 V_SSCE_ for 100 s from 0.1 mmol/L K_2_PtCl_4_ and 0.5 mol/L NaCl. The dotted lines demark the location of steps on the Au(111) substrate.

Some of the concepts involved in self-limited electrodeposition reactions overlap with other strategies for ultrathin film deposition. For example, a class of self-assembled monolayer films is of particular relevance, wherein metal cation precursors are tethered to the surface using molecules with anionic end-groups.^37,38^ The present effort builds upon this and related work^4,7,8,11,13,15,16,28^ by using potential control to isolate the interfacial adsorption step from the reduction step in a simple, single-precursor-containing electrolyte. This scheme represents a simplification over previous Pt electrodeposition processes that have been either obscured by overlap with proton reduction and H_ads_ or masked by multistep reductions associated with Pt­(IV) precursors and mixed complexes. In a further complication, alloying interactions can also accompany redox replacement reactions between Pt­(IV)/Pt­(II)/Pt and reducing agents such as Pb­(II)/Pb and Cu­(II)/Cu.^1,5,6,9,10,14,22,25,29,66–68^ In contrast, by working with weakly acidic, halide-rich electrolytes combined with freshly prepared pristine Au surfaces, the minimal conditions for surface-limited growth have been isolated. Optimization of the precursor concentration and potential waveform allows self-limiting monolayer deposition to be tuned for a given coverage. More generally, by coupling the reduction of PtCl_4_^2–^_aq_ with certain reducing agents and substrates, self-terminated deposition can be realized, provided the mixed potential of the fully comprised system resides within the gap between the onset of two-dimensional monolayer deposition and three-dimensional growth. Extension of this simple approach to electrochemical atomic layer deposition to other adsorbate–substrate combinations should enable more refined synthesis of metal-overlayer thin films and core–shell nanoparticles.

## Conclusion

Electrochemical formation of Pt monolayer films on Au is achieved by surface-specific, potential-controlled, irreversible adsorption of PtCl_4_^2–^_ads_ followed by its reduction over a limited range of more negative potentials. In pH 3.5 NaCl-based electrolytes, a distinct reduction wave for the reduction of PtCl_4_^2–^_ads_ layer on Au is evident that is otherwise absent for Pt-covered surfaces. At +0.40 V_SSCE_, PtCl_4_^2–^_aq_ exhibits a strong affinity for Au, while its interaction with Cl^–^ covered Pt is minimal. XPS analyses indicate that the fractional coverage of adsorbed PtCl_4_^2–^_ads_ can be tuned by adjusting the electrolyte composition, with saturated coverage for (111)-textured PVD Au films ranging between 0.13 and 0.10, depending on Cl^–^ concentration. The surface-selective nature of PtCl_4_^2–^_ads_ formation and its reduction at potentials between +0.1 V_SSCE_ and −0.2 V_SSCE_ leads to the nucleation and growth of Pt films limited to monolayer dimensions.

Electrodeposition under potentiostatic, potentiodynamic, and/or pulsed potential waveforms demonstrates that self-limiting growth of Pt monolayer films is achievable by adjusting K_2_PtCl_4_ concentration and controlling the potential to avoid three-dimensional growth. Excessive Pt precursor concentration can lead to additional growth beyond a monolayer due to competitive adsorption with Cl^–^. Pulsed potential deposition is particularly useful in separating the PtCl_4_^2–^_ads_ adsorption step from its electrochemical reduction. When implemented with specific waveform parameters and electrolyte concentrations, this enables the deposition step to be restricted to the preadsorbed PtCl_4_^2–^_ads_ layer. The same approach can be used to explore the deposition of self-limiting monolayer films based on other precursor adsorbates and overlayer-substrate systems.

## Experimental Section

### Substrate Preparation

#### Au PVD on Si

The Au-seeded wafers were prepared by electron-beam-heated physical vapor deposition of a 10 nm Ti adhesion layer, followed by a 100 nm Au overlayer on Si(100) wafers at a deposition rate of (1.0 to 1.5) Å/s. The wafers were cleaved into 0.5 cm × 1.5 cm rectangular electrodes. Immediately prior to use, the electrodes were cleaned by immersion in piranha solution^45^ for 60 min with both Au and Si sides exposed, rinsed with 18 MΩ·cm water, and dried under an Ar stream. The electrodes were then masked with Kapton tape to expose an area of ≈0.35 cm^2^ (0.5 cm × 0.7 cm) on the Au side only. To account for uncertainty in electrode area determination, the error in charge density was estimated based on ruler resolution (0.5 mm divisions). Assuming a reading error of ±0.25 mm in both width and length, the propagated uncertainty in exposed area was approximately 8.8%, which was applied to current density and charge-derived values.

#### Au PVD on Mica

Au thin films were deposited onto mica substrates by DC magnetron sputtering. The mica substrates were freshly cleaved using adhesive tape immediately before loading in the vacuum chamber. The substrate stage was nominally set to maintain at 600 °C (corresponding to an estimated substrate temperature of ∼250 °C), while the chamber was evacuated to a base pressure of 1.6 × 10^–6^ Torr. Argon was introduced at a flow rate of 10 sccm, resulting in a working pressure of ∼1.5 × 10^–3^ Torr. A 3.0 in. Au target was used. After a presputtering step (50 s), sputtering was carried out at a constant current of 0.2 A (∼72 W) with the substrate rotated at 50 rpm for 500 s. The deposition rate was approximately 0.04 nm/s. After sputter deposition, the sample was cooled to below 50 °C prior to removal. Prior to Pt electrodeposition, the (111)-textured Au mica films were flame annealed in a H_2_ flame for 5 to 10 min.

### Pt Electrodeposition Procedure

Platinum deposition was conducted at 23 °C in a water-jacketed three-electrode glass cell. The cell featured an Au working electrode, with an Ir wire counter electrode. Both the counter and reference electrodes were held in separate compartments connected to the main chamber via fritted junctions filled with the same supporting electrolyte as the electrodeposition bath, i.e., excluding the Pt salt. The reference electrodes were chosen to match the electrolyte system: a saturated sodium chloride calomel electrode (SSCE) for NaCl-based electrolytes and K_2_SO_4_ saturated sulfate electrode (SSE) for experiments in HClO_4_ and H_2_SO_4_ electrolyte. The Pt electrodeposition bath consisted of 0.5 mol/L NaCl or 4.0 mol/L NaCl containing K_2_PtCl_4_ or K_2_PtCl_6_, with the pH adjusted to 3.5 using diluted HCl. For Cl^–^-free control experiments, 0.1 mol/L HClO_4_ was used without further pH adjustment. All electrolytes were deaerated by Ar purging for 30 min prior to use. During electrodeposition, Ar was not bubbled directly into the electrolyte but instead was redirected to blanket the solution within the cell.

In the two-step electrolyte transfer Pt deposition experiments, derivatization was performed in K_2_PtCl_4_-containing electrolytes composed of either 4.0 mmol/L NaCl or 0.1 mol/L HClO_4_, followed by rinsing with dilute HCl (pH 3.5), instead of deionized water, prior to shuttling into a Pt-free electrolyte. Without drying, the rinsed specimens were immediately transferred into the Pt-free electrolyte for voltammetric reduction. For XPS analysis, the electrodes were emersed after the electrochemical procedure, rinsed with deionized water, gently dried, and shielded with Ar gas during transfer to the spectrometer.

### XPS Analysis Procedure

The Au thin film specimens were immersed into the electrolyte with the applied potential already engaged. Likewise, during emersion, the specimens were immediately rinsed with 18 MΩ·cm water or pH 3.5 HCl before disconnecting from the potentiostat. The Pt-Au films were characterized by XPS using a Kratos AXIS Ultra DLD instrument with a monochromatic Al K_α_ X-ray source.[Fn fn1] Survey spectra were collected using a pass energy of 80 eV while 20 eV was used for high-resolution scans (Pt 4f, Cl 2p, O 1s). Further information on the XPS measurements and data analysis is provided in the Supporting Information.

### Scanning Tunneling Microscopy Measurements

All measurements were performed with a Molecular Imaging 4500 microscope. Once the substrate and tip were installed, the microscope base was sealed and flooded with Ar. The PtIr tips were prepared using a two-step AC etching process with a saturated CaCl_2_ aqueous electrolyte. A coarse etch, in a beaker cell with a carbon rod counter electrode was used to produce an elongated tear-drop shape by immersing the wire 2 mm into the electrolyte and applying 25 V_rms_.^69^ Etching was stopped when the current fell below 40 mA AC. The partially etched PtIr workpiece was then transferred to a two-dimensional cell where a lamella of electrolyte is formed within a tungsten ring counter electrode. The PtIr wireis immersed orthogonal to the lamella to align the minimum wire diameter of the tear-drop shape. Pulsed AC etching was performed with 2.5 V_RMS_ until the bottom section separated from the PtIr workpiece, leaving a sharp tip. In some cases, the tip could be further modified or refurbished by performing a brief etch while removing the PtIr tip from the lamella.

## Supplementary Material



## Footnotes

1 Certain commercial equipment, instruments, or materials are identified in this paper to specify the experimental procedure adequately. Such identification is not intended to imply recommendation or endorsement by the National Institute of Standards and Technology, nor is it intended to imply that the materials or equipment identified are necessarily the best available for the purpose.

## Notes

Additional experiment details; coulometric analysis, XPS binding energy references, sensitivity factors, and attenuation length, six quantitative XPS models, Pt chloride complex speciation, voltammetry, XPS data including reference standards and deconvolution procedures, and PtCl (PDF)
