# Supplementary material for: Atomic Layer Electrodeposition of a Pt Monolayer on Au via Surface-Specific PtCl4 2– Adsorption
Source: Chem Mater. 2026 Jul 16;38(15):7914–28. doi: 10.1021/acs.chemmater.6c01182 (PMC13471728; doi:10.1021/acs.chemmater.6c01182)
Supplement: Supplementary file 1 [file cm6c01182_si_001.pdf]

Supplementary Information:

Atomic Layer Electrodeposition of a Pt monolayer on Au via Surface-Specific $PtCl_4^{2-}$ Adsorption

Hyunki Kim, Thomas P. Moffat*

Materials Science and Engineering Division

National Institute of Standards and Technology

Gaithersburg, Maryland 20899

Table S1

$PtCl_4^{2}$ reduction charge to Pt on Au as a function of K_2PtCl_4 in 0.5 mol/L (NaCl pH=3.5). The values were extracted from the voltammetric curves shown in **Figure 1a**.

0.5 mol/L NaCl + 0.01-10.0 mmol/L K_2PtCl_4 (pH 3.5)

Concentration of K_2PtCl_4 / mmolL^{-1}	Background : y=0		Background : straight line	
	Charge density / mC cm^{-2}	Pt monolayer / ML	Charge density / mC cm^{-2}	Pt monolayer / ML
0.01	0.1495 (0.40 V to -0.10 V)	0.31	0.042475 (0.25 V to -0.10 V)	0.09
0.05	0.276 (0.40 V to -0.10 V)	0.58	0.152 (0.25 V to -0.10 V)	0.32
0.10	0.338 (0.40 V to -0.10 V)	0.7	0.2005 (0.25 V to -0.10 V)	0.42
1.00	0.615 (0.40 V to -0.10 V)	1.28	0.411 (0.25 V to -0.10 V)	0.86
10.00	0.6655 (0.40 V to -0.05 V)	1.39	0.418 (0.23 V to 0.05 V)	0.87

Table S2

X-ray photoelectron spectroscopy (XPS) binding energy reference values

Pt 4f and Cl 2p regions for metallic Pt, Pt salt, and $PtCl_4^{2-}{}_{ads}$/Au.

Element	Neutralizer	Normalization by	$Pt4f_{7/2}$	$Pt4f_{5/2}$	$Cl2p_{3/2}$	$Cl2p_{1/2}$	C1s	Reference
K_2PtCl_4 salt	On	*Au 4f (84.0 eV, offset from ref. Au wafer) Applied offset: 3.9 eV	73.3 eV	76.6 eV	199.2 eV	200.8 eV	285.2 eV	This study
K_2PtCl_6 salt	On	*Au 4f (84.0 eV, offset from ref. Au wafer) Applied offset: 3.9 eV	75.6 eV	78.9 eV	199.1 eV	200.7 eV	285.0 eV	This study
0.4 ML Pt/Au	On	*Au 4f (84.0 eV, offset from ref. Au wafer) Applied offset: 3.9 eV	71.0 eV	74.3 eV	197.7 eV	199.4 eV	284.1 eV	This study
K_2PtCl_4 salt	On	C 1s (285.0 eV)	73.1 eV	76.4 eV	199.0 eV	200.6 eV	-	This study
K_2PtCl_6 salt	On	C 1s (285.0 eV)	75.6 eV	78.9 eV	199.1 eV	199.5 eV	-	This study
0.4 ML Pt/Au	On	C 1s (285.0 eV)	71.9 eV	75.2 eV	198.6 eV	200.3 eV	-	This study
Monolayer Pt/Au	Off	Au 4f (84.0 eV)	70.9 eV	74.2 eV	-	-	~284.0 eV	This study
$PtCl_4^{2-}{}_{ads}$/Au	Off	Au 4f (84.0 eV)	72.2 eV	75.5 eV	197.6 eV	199.2 eV	~284.0 eV	This study
Pt metal standard	Off	-	70.9 eV	74.2 eV	-	-	-	This study
$PtCl_4$	-	C 1s (285.0 eV)	76.1 eV	-	-	-	-	J. Catal. 139, 191
K_2PtCl_6	-	C 1s (285.0 eV)	75.8 eV	-	199.7 eV	-	-	J. Chem. Soc. Dalton Trans. 1293
K_2PtCl_4	-	C 1s (285.0 eV)	73.4 eV	-	199.8 eV	-	-	J. Chem. Soc. Dalton Trans. 1293
Pt foil	-	C 1s (285.0 eV)	71.0 eV	74.3 eV	-	-	-	J. Chem. Soc. Dalton Trans. 1293
K_2PtCl_6	-	C 1s (285.0 eV)	75.9 eV	-	199.4 eV	-	-	Organometallics 6, 28
K_2PtCl_4	-	C 1s (285.0 eV)	73.4 eV	-	199.4 eV	-	-	Organometallics 6, 28
K_2PtCl_4	-	C 1s (285.0 eV)	73.2 eV	-	199.0 eV	-	-	J. Catal. 48, 217
K_2PtCl_4	-	C 1s (285.0 eV)	73.3 eV	-	199.3 eV	-	-	Inorg. Chem. 31, 4704
K_2PtCl_6	-	Au 4f (83.8 eV)	75.0 eV	-	198.5 eV	-	-	J. Electron Spectrosc. Relat. Phenom. 1, 193

Table S3

Summary of binding energy, kinetic energy, relative sensitivity factor (rsf) for elements.

Species	B. E / eV	K. E / eV	Relative sensitivity factor / $I_{i,rsf}$
Au 4f	83.8	1402.8	6.25
Pt 4f	70.68	1415.92	5.575
Cl 2p	198.2	1288.4	0.891

Table S4

Attenuation length from equation S1.

Attenuation length in monolayers	$K_i^j = 1 - e^{-a_j/L_j(E_i)}$
$L_{Pt}^{Pt} = 5.360$ ML	$K_{Pt}^{Pt} = 0.1702$
$L_{Au}^{Pt} = 5.320$ ML	$K_{Au}^{Pt} = 0.1714$
$L_{Cl}^{Cl} = 8.189$ ML	$K_{Cl}^{Cl} = 0.1150$
$L_{Au}^{Cl} = 8.806$ ML	$K_{Au}^{Cl} = 0.107$

Table S5

Summary of the six quantitative XPS models used to evaluate Pt and Cl coverages on Au.

Case	Key equation	Assumption	Considered layers
1	$d = L\cos\theta \ln[1 + \frac{I_{Pt}/I_{Pt\,rsf}}{I_{Au}/I_{Au\,rsf}}]$	**Uniform continuous Pt overlayer; Cumpson–Seah homogeneous film model[3]**	**Pt only**
2	$Ratio_{Au}^{Pt} = \frac{I_{Pt} I_{Au,\infty}}{I_{Pt,\infty} I_{Au}}$ $\theta_{Pt} = Ratio_{Au}^{Pt} \frac{L_{Pt}}{a_{Pt}} \cos\theta$	**Uniform continuous Pt overlayer; Shard bulk-referenced model using metallic Pt and thick Au standard[4]**	**Pt only**
3	$Ratio_{Au}^{Pt} = \frac{\frac{I_{Pt}}{I_{Pt\,rsf}}}{\frac{I_{Au}}{I_{Au\,rsf}}}$ $\theta_{Pt} = Ratio_{Au}^{Pt} \frac{L_{Pt}}{a_{Pt}} \cos\theta$	**Uniform continuous Pt overlayer; Case 2 modified to use rsf-normalized intensities instead of bulk reference**	**Pt only**
4	$Ratio_{Au}^{Pt} = \frac{\frac{I_{Pt}}{I_{Pt\,rsf}}}{\frac{I_{Au}}{I_{Au\,rsf}}} = \frac{\theta_{Pt} K_{Pt}^{Pt}}{1-\theta_{Pt} K_{Au}^{Pt}}$ $\theta_{Pt} = \frac{Ratio_{Au}^{Pt}}{K_{Pt}^{Pt} + Ratio_{Au}^{Pt} K_{Au}^{Pt}}$	**Pt Sub-monolayer (partial coverage); thin-film attenuation model for non-uniform Pt layer**	**Pt only**
5	$Ratio_{Au}^{Pt} = \frac{\frac{I_{Pt}}{I_{Pt\,rsf}}}{\frac{I_{Au}}{I_{Au\,rsf}}} = \frac{\theta_{Pt} K_{Pt}^{Pt}}{1-\theta_{Pt} K_{Au}^{Pt} - \theta_{Cl} K_{Au}^{Cl}}$ $\theta_{Cl} = \frac{[Ratio_{Au}^{Cl}] K_{Pt}^{Pt}}{K_{Cl}^{Cl} K_{Pt}^{Pt} + [Ratio_{Au}^{Cl}] K_{Au}^{Cl} K_{Pt}^{Pt} + [Ratio_{Au}^{Pt}] K_{Au}^{Pt}}$ $\theta_{Pt} = \frac{[Ratio_{Au}^{Pt}](1-\theta_{Cl} K_{Au}^{Cl})}{K_{Pt}^{Pt} + [Ratio_{Au}^{Pt}] K_{Au}^{Pt}}$	**Sub-monolayer co-adsorption model[5] (Pt and Cl layers attenuate each other); extended from Case 4**	**Pt and Cl**
6	$Ratio_{Au}^{Pt} = \frac{\frac{I_{Pt}}{I_{Pt\,rsf}}}{\frac{I_{Au}}{I_{Au\,rsf}}} = \frac{\theta_{Pt} K_{Pt}^{Pt}}{1-\theta_{Pt} K_{Au}^{Pt} - \theta_{Cl} K_{Au}^{Cl}}$ $\theta_{Pt} = \frac{[Ratio_{Au}^{Pt}](1-\theta_{Cl} K_{Au}^{Cl})}{K_{Pt}^{Pt} + [Ratio_{Au}^{Pt}] K_{Au}^{Pt}}$, and $\theta_{Cl} = 4\theta_{Pt}$ $\theta_{Pt} = \frac{[Ratio_{Au}^{Pt}]}{K_{Pt}^{Pt} + [Ratio_{Au}^{Pt}](K_{Au}^{Pt} + 4K_{Au}^{Cl})}$	**Sub-monolayer co-adsorption model; Case 5 modified with fixed Cl/Pt ratio of 4**	**Pt and Cl**

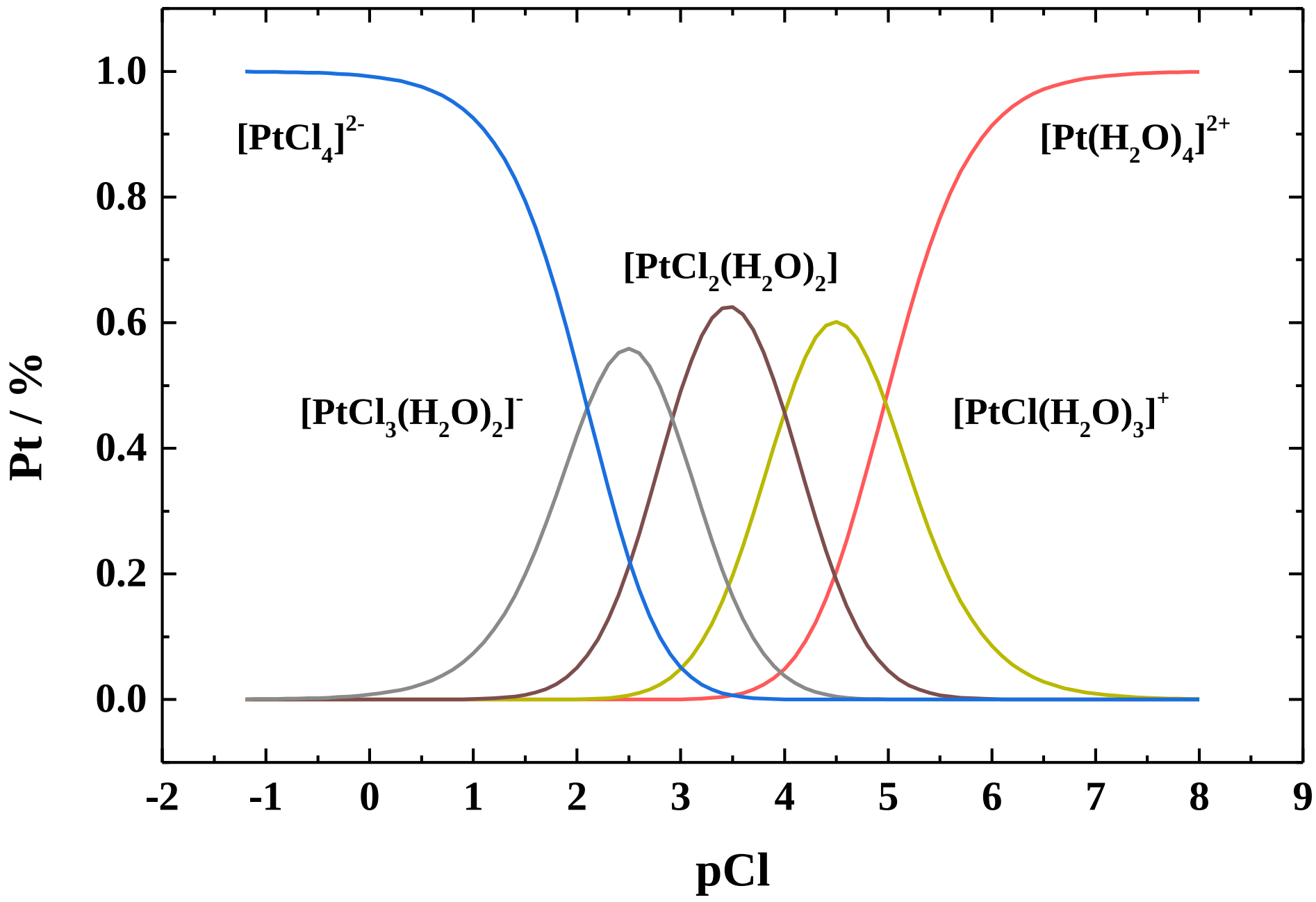

Fig. S1 Formation diagram of $(PtCl_x(H_2O)_{4-x})^{2-x}$ as a function of pCl.

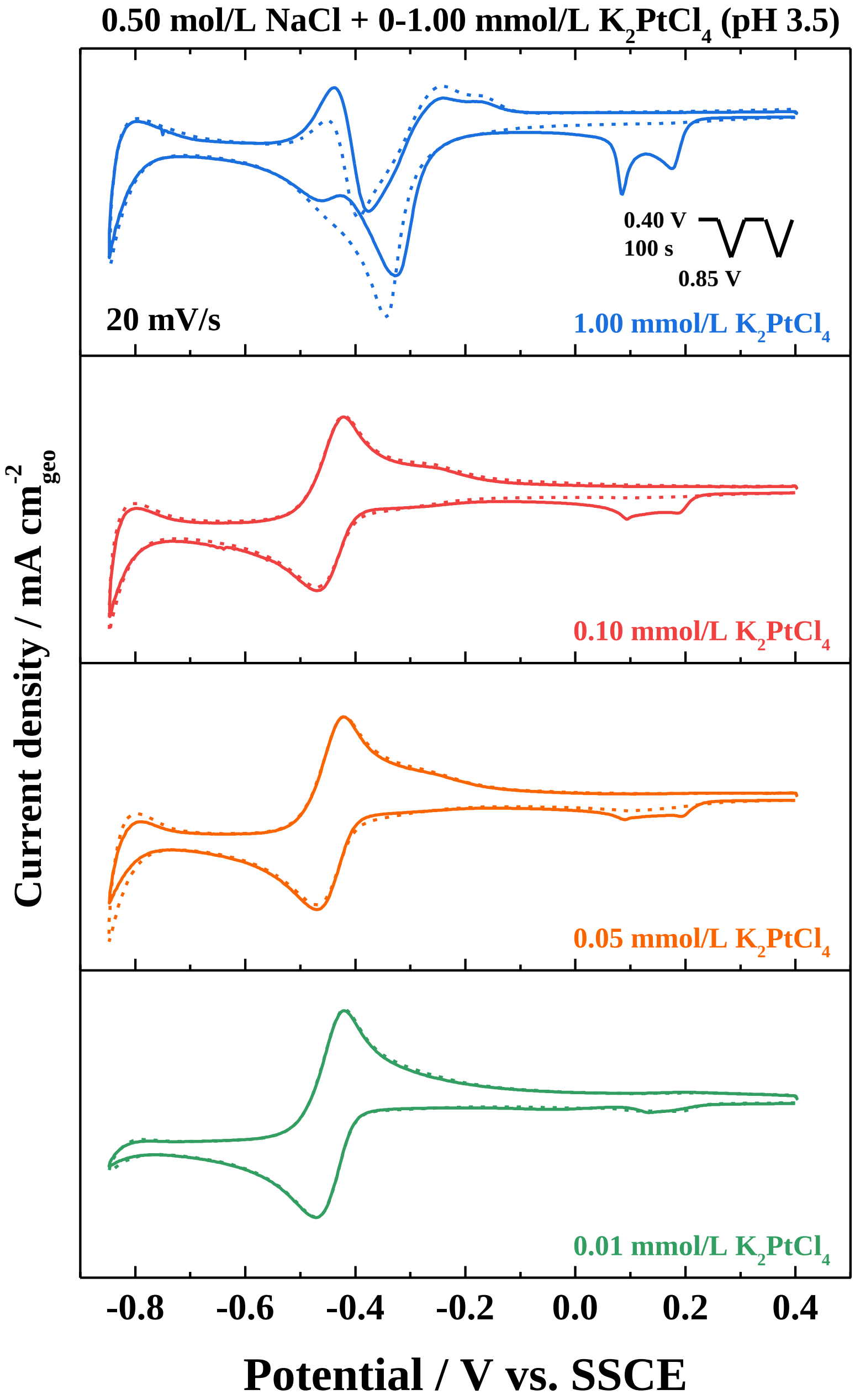

Fig. S2 Two consecutive voltammograms for different K_2PtCl_4 concentrations. in 0.5 mol/L NaCl and with (0.01 to 1.0) mmol/L K_2PtCl_4.

Voltammetry at different K_2PtCl_4 concentrations reveal similar features with some notable variations. As the $PtCl_4^{2-}{}_{aq}$ concentration decreases, the two waves for selective Pt monolayer deposition on Au decrease as does the peak for bulk Pt deposition. These changes reflect the mass transport and kinetic limitation on $PtCl_4^{2-}{}_{aq}$ adsorption both during the 100 second dwell at +0.40 V_{SSCE}, and the subsequent reduction scan. Crucially, for $K_2PtCl_4 \geq 0.1$ mmol/L the voltammetric waves for surface selective two-dimensional (2D) growth (near +0.175 V_{SSCE} and +0.08 V_{SSCE}) are completely absent on the second scan as the surface is largely covered by Pt deposition during the first scan. In contrast, for 0.01 mmol/L K_2PtCl_4 the first two cycles are indistinguishable, consistent with the availability of free Au sites for adsorption and reduction of $PtCl_4^{2-}$ with the net amount of Pt deposited being limited by transport. Beyond surface selective deposition, the similarity of the first and second voltammetric cycles for $\leq$ 1 mmol/L K_2PtCl_4 speaks to negligible roughening of the electrode, while for more concentrated solutions roughening due to 3D film growth between -0.2 V_{SSCE} and -0.450 V_{SSCE} is evident in the expanded H_{upd} and H_2 oxidation waves.

Further delineation of the contributions from surface-selective deposition versus bulk Pt deposition can be obtained by restricting the voltammetric window and exploring the scan rate or temporal response. Likewise, the impact of Cl^- concentration is evident in **Fig. S4** with the negative shift of the peak potential for both surface-selective reduction of $PtCl_4^{2-}$ and bulk Pt deposition in line with thermodynamic expectations.[1-3] At the same time, the net charge of the surface waves is largely maintained (**Table S1**).

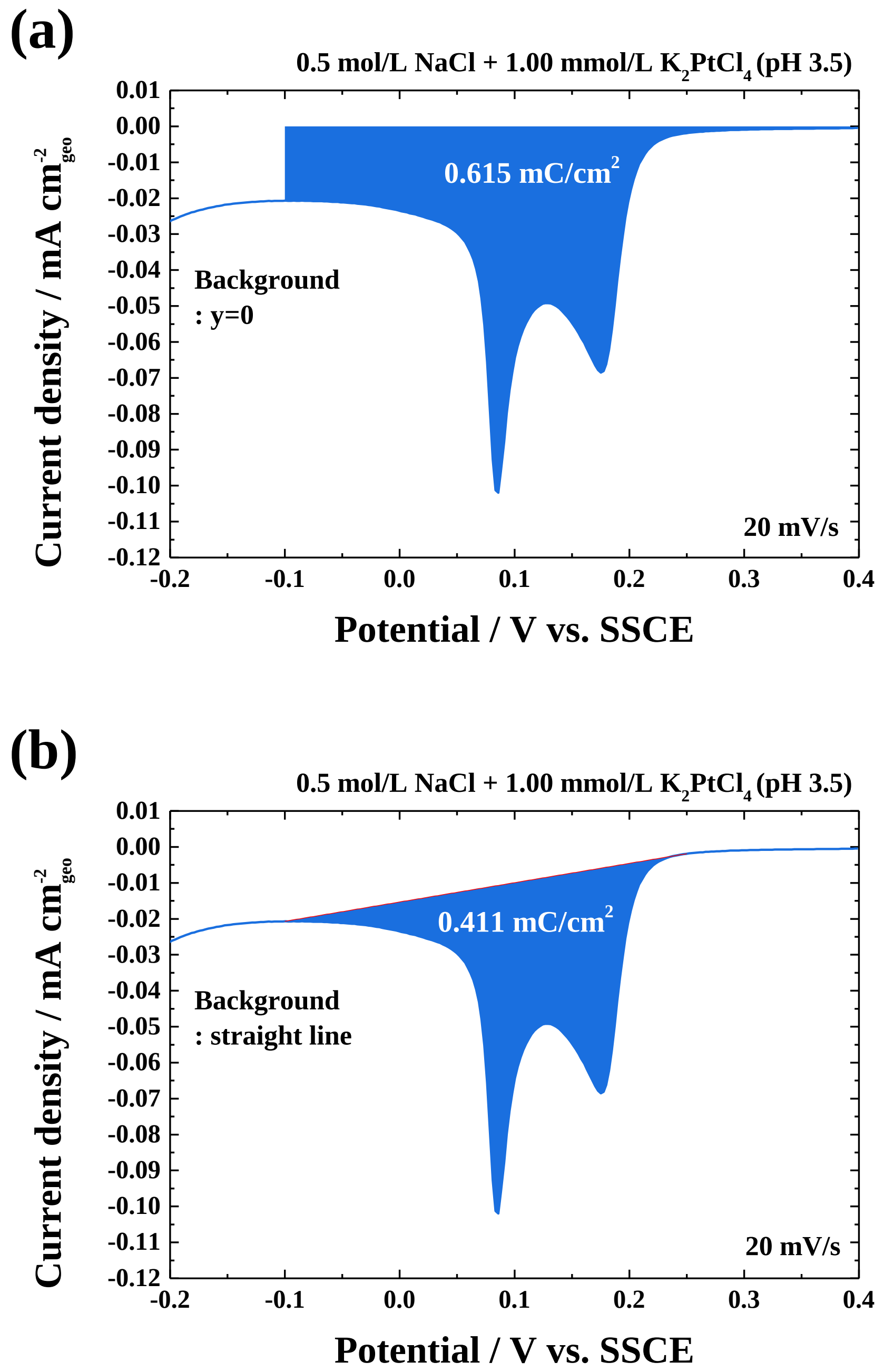

Fig. S3 Integrated charge of Au cyclic voltammetry in 1.00 mmol/L containing 0.5 mol/L NaCl K_2PtCl_4 after 100 s pre-hold at 0.40 V_{SSCE} with different background. The baseline was defined as (a) y=0, and (b) linear fit to the background with the lower vertex corresponding to local minima in current density.

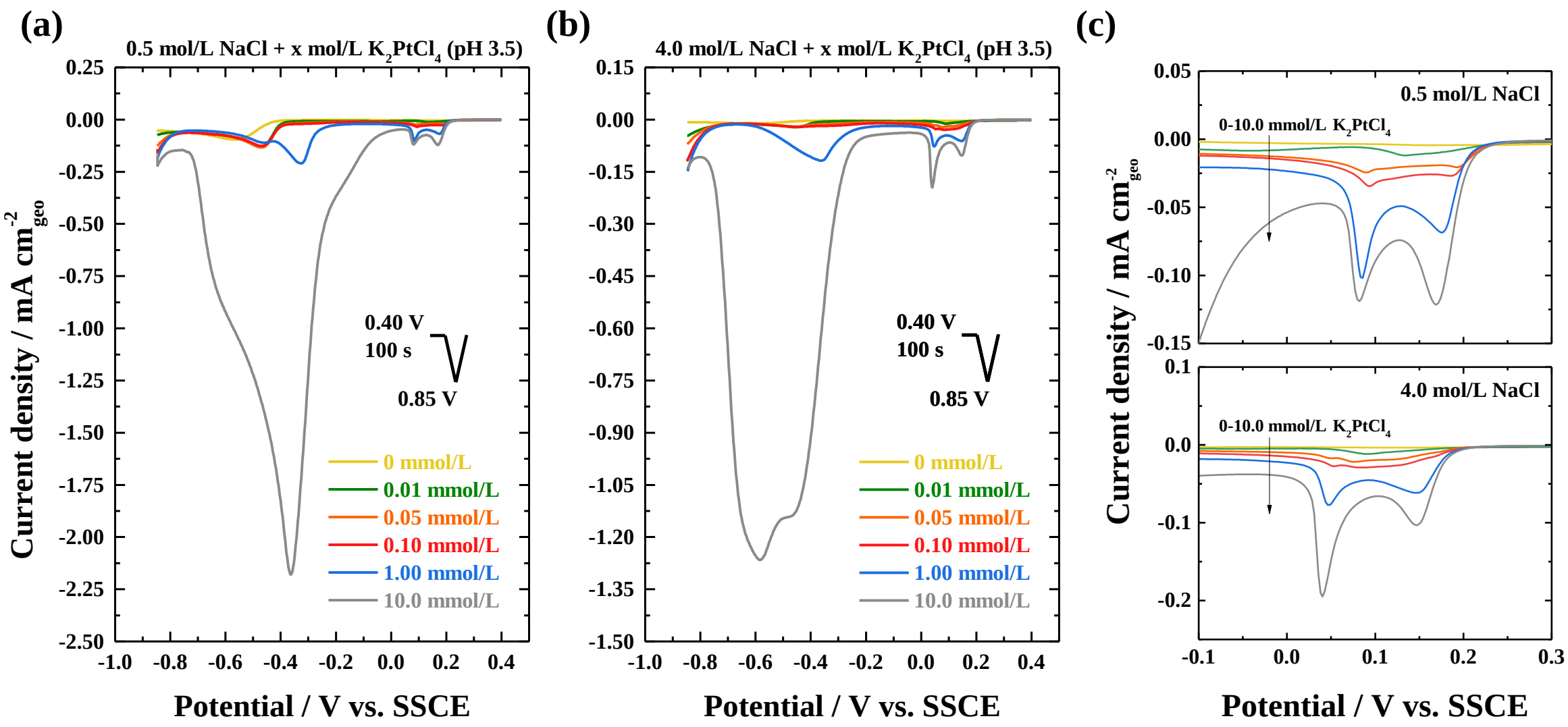

Fig. S4 First voltammetric cycle at 20 mV/s for Pt deposition on Au in (a) 0.5 mol/L NaCl and (b) 4.0 mol/L NaCl with (0.01 to 10.0) mmol/L K_2PtCl_4. (c) The surface-selective Pt deposition waves on Au are dependent on both K_2PtCl_4 and NaCl concentration.

(a)

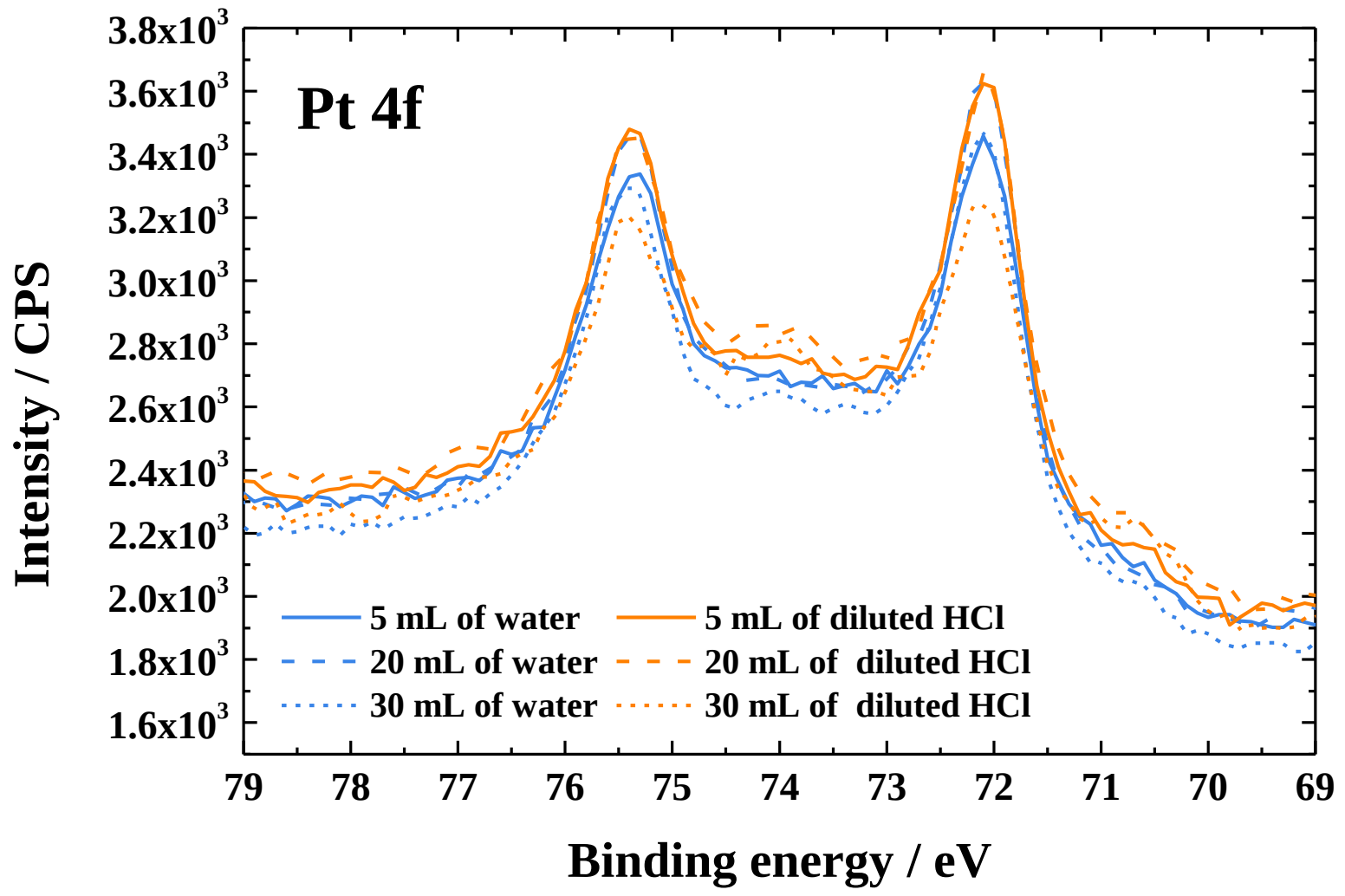

(b)

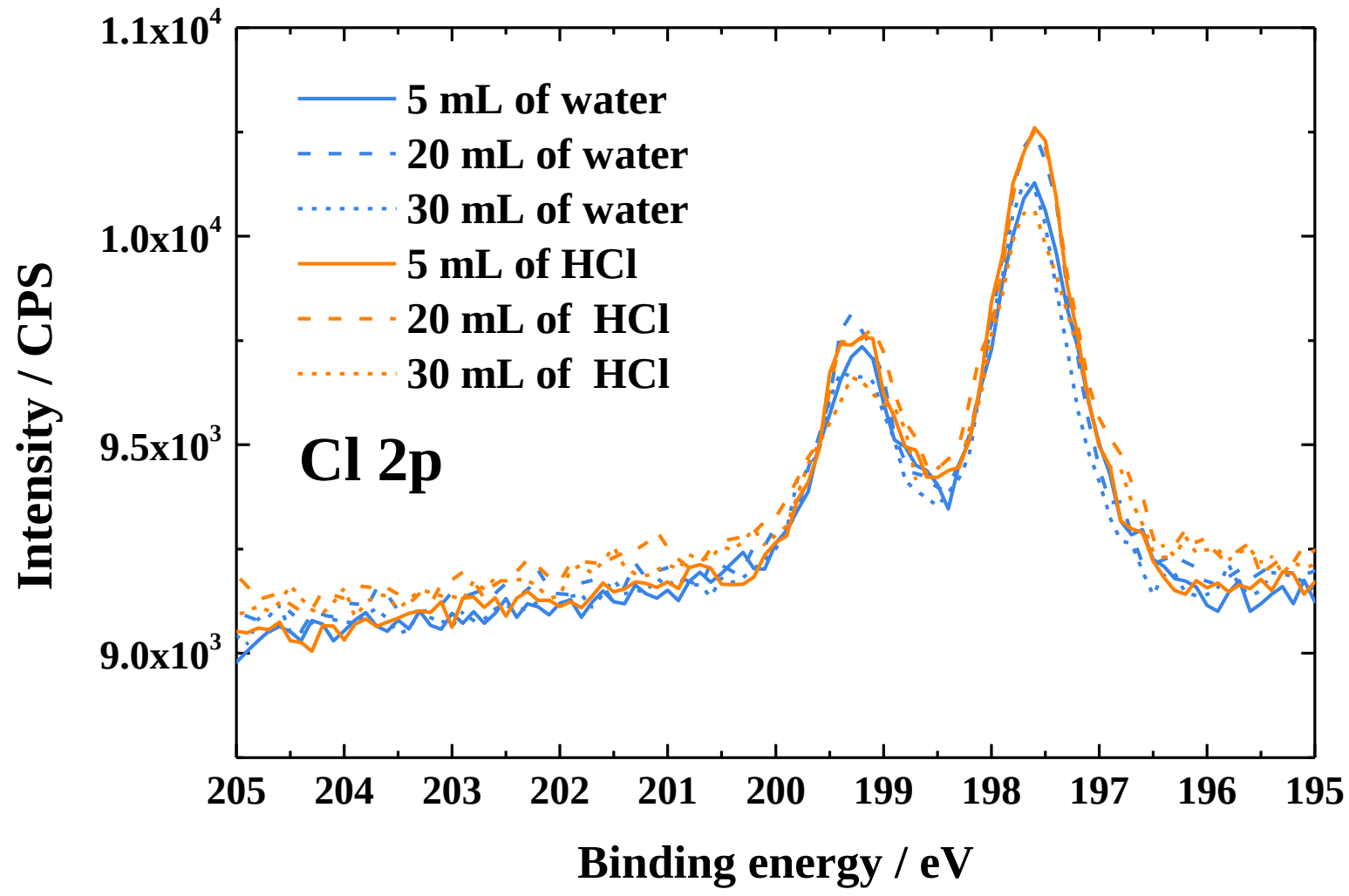

Fig. S5 XPS spectra of $PtCl_4^{2-}{}_{ads}$/Au after rinsing with (5 to 30) mL of either Ar-purged deionized water or dilute HCl (pH 3.5). (a) Pt 4f and (b) Cl 2p regions. The adsorption step was conducted at 0.40 V_{SSCE} for 100 s in 0.5 mol/L NaCl with 0.10 mmol/L K_2PtCl_4.

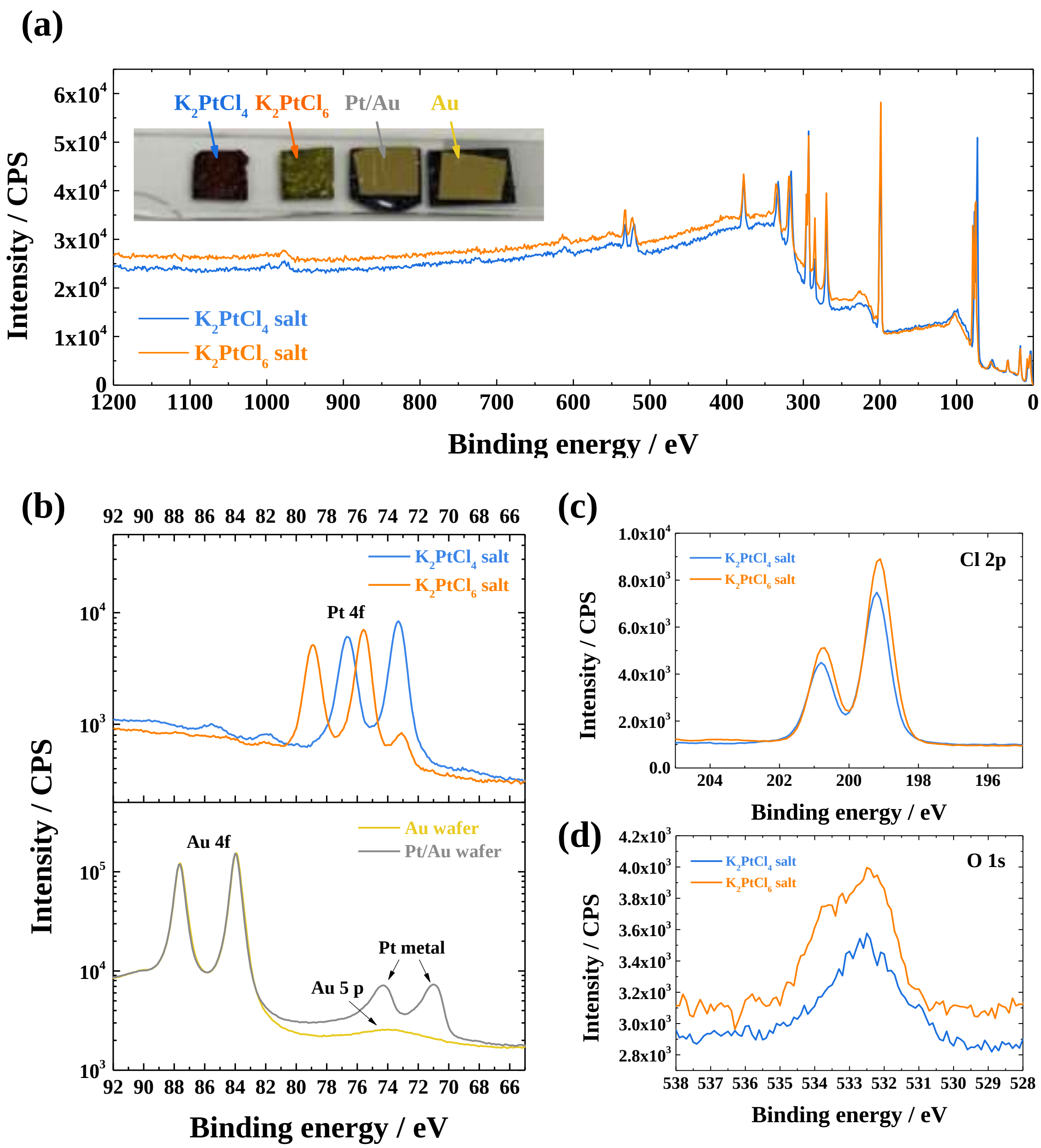

Fig. S6 XPS spectra of K_2PtCl_4 and K_2PtCl_6 salt. (a) Survey spectra of both salts. Inset: optical image of the salts prepared on carbon tape for XPS analysis. (b) Pt 4f spectra compared with those from a bare Au wafer and Pt-deposited Au. (c) Cl 2p and (d) O 1s regions.

XPS measurements of the K_2PtCl_4 and K_2PtCl_6 standards were performed by electrically isolating the XPS sample stage from the spectrometer. The salts were mounted on carbon tape that had been affixed to glass slides, and spectra were collected using an electron flood gun to compensate for surface charging. The flood gun settings were 4.2 V charge balance, 1.85 A filament current, and 1.5 V filament bias. For comparison and calibration, bare Au wafers and Pt-coated Au wafers were also mounted on carbon tape and analyzed sequentially under a near identical X-ray flux. Spectra were acquired using a monochromatic Al Kα source, with survey scans collected at a pass energy of 80 eV and high-resolution spectra for Pt 4f, Cl 2p, and O 1s obtained at 20 eV. After acquisition, all spectra were calibrated using the Au $4f_{7/2}$ peak set to 84.0 eV from the concurrently measured Au wafer. The resulting +3.9 eV offset determined from the Au reference was then applied to the salt spectra to correct for charging effects. In addition, the C 1s peak of each carbon tape (set to 285.0 eV) was used as an alternative internal reference for comparison, and the results of both calibrations are summarized in **Table S2**.

Spectra (20 eV pass energy) were acquired with 12 scans over the Pt 4f region (including Au 4f), 20 scans for Cl 2p, and 5 scans each for O 1s and C 1s. Three scans were collected for each (80 eV pass energy) wider energy survey recorded. During repeated scans of K_2PtCl_6, a peak at 73.1 eV, which coincides with the Pt $4f_{7/2}$ peak of K_2PtCl_4, was observed to increase progressively. This behavior is consistent with partial disproportionation of K_2PtCl_6 to K_2PtCl_4 under X-ray exposure.[4] A sensitivity normalized Cl 2p/Pt 4f intensity ratio for K_2PtCl_4 was measured to be 4.03, in agreement with its stoichiometric composition, whereas that of

K_2PtCl_6 was slightly lower than the expected value of 6.0, at 5.748, due to the disproportionation.

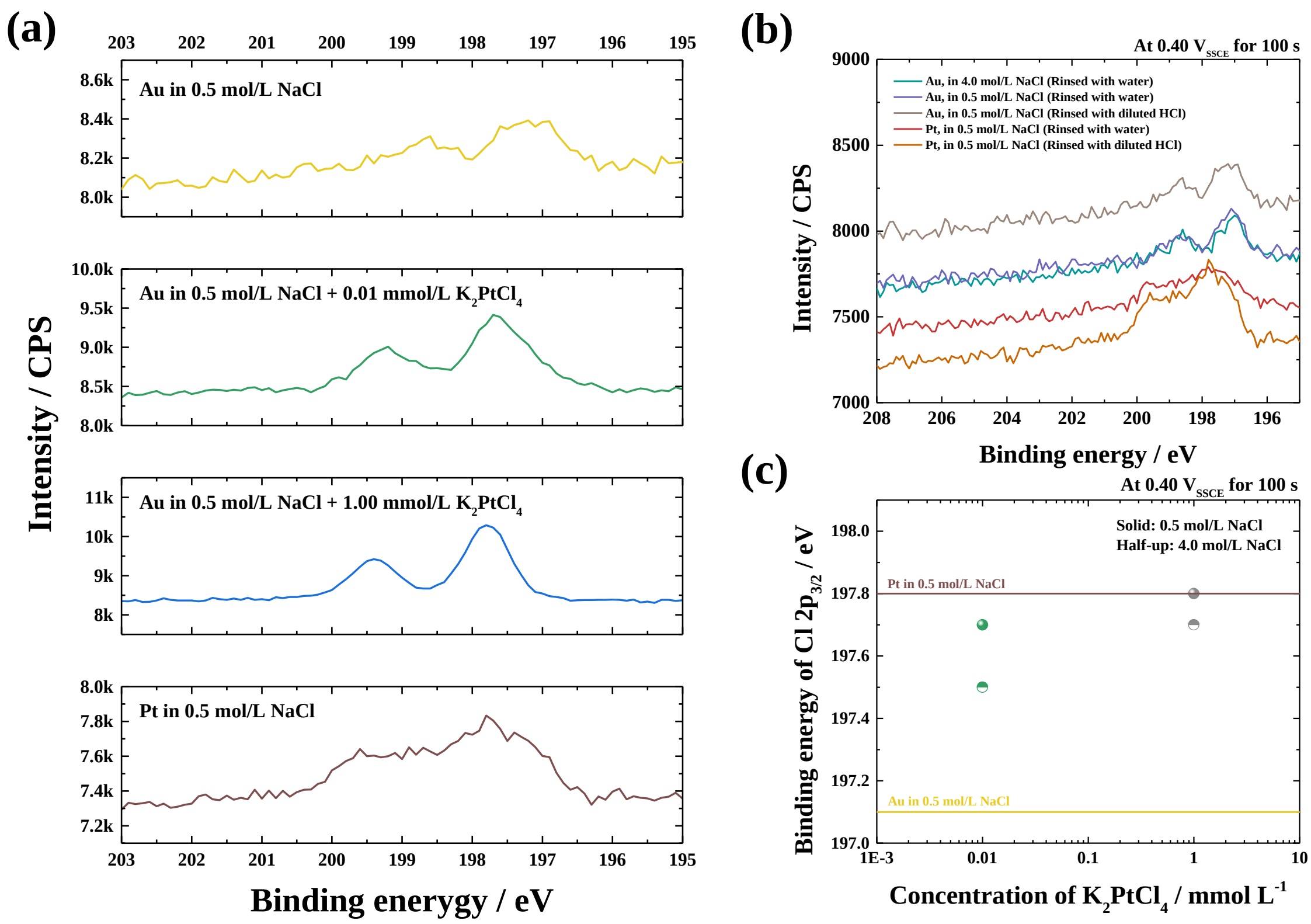

Fig. S7 XPS characterization of Cl 2p for Au and Pt after derivatization in 0.5 mol/L NaCl at +0.40 V_{SSCE} for 100 compared with $PtCl_4{}^{2-}{}_{ads}$ adlayer on Au following derivatization in (0.5 to 4.0) mol/L NaCl containing 0.01 mmol/L or 1.0 mmol/L K_2PtCl_4.

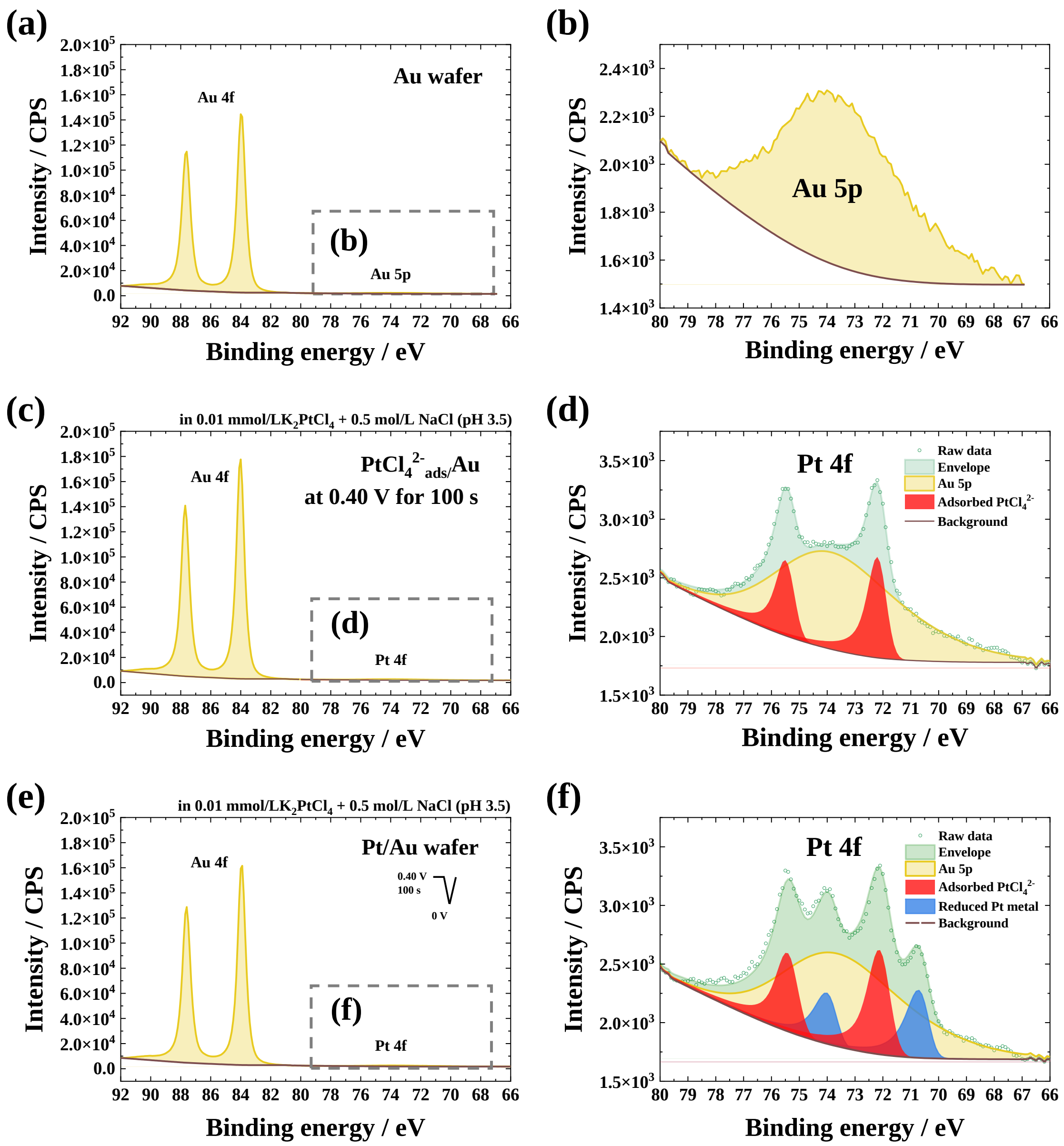

Fig. S8 Au 4f and Pt 4f spectra of (a,b) Au wafer, (c,d) $PtCl_4^{2-}{}_{ads}$/Au and (e,f) $PtCl_4^{2-}{}_{ads}$Pt/Au films.

High-resolution Pt (4f) and Au(4f) spectra were deconvoluted using CasaXPS software (version 2.3.26PR1.0, Casa Software Ltd.). A Tougaard background was employed for Au and

Pt 4f regions, while a linear background was applied to Cl 2p as detailed further in the supplement. For submonolayer Pt films, accurate deconvolution of the Pt 4f region required exclusion of the overlapping Au 5p contribution. To establish the Au 5p to Au 4f intensity ratio, a clean Au wafer was used as a reference after surface treatment with piranha solution, and spectra were analyzed using Tougaard background subtraction (a,b). The Pt 4f and Au 5p peak shape were fitted using asymmetric Lorentzian (LA) and Lorentzian–Gaussian (LF) function line shapes, respectively. LA(1.2, 85, 70) and LA(0.75, 10, 250) were used for metallic Pt on Au and $PtCl_4^{2-}{}_{ads}$ on Au. LF(1, 0.2, 30, 500) was used for Au 5p peaks. The Au (5p)/Au (4f) ratio for clean Au reference was used to subtract the Au 5p contribution from the Pt 4f spectra.

Relative sensitivity factors (rsf) based on the Kratos calibration ($S_{F\ 1s}$ = 1.00) were applied, with $S_{Au\ 4f}$ = 6.25, $S_{Pt\ 4f}$ = 5.575, and $S_{Cl\ 2p}$ = 0.891 (**Table S3**). The attenuation lengths (L) of the photoelectrons were estimated using Seah's S4 universal curve, which accounts for energy-dependent electron scattering in all materials.[5]

$$L/a = (1.861 + 0.00132Z^{1.7} + 0.028E^{0.93})/\ Z^{0.38} \quad [1]$$

where a represents the atomic size of Pt, Z is the atomic number, and E is the kinetic energy of the relevant photoelectron. The resulting attenuation lengths were summarized in **Table S4**.

To evaluate the $PtCl_4^{2-}{}_{ads}$ coverages on Au, six different models were compared. These models differ mainly in two aspects: the structural assumption of the film (uniform overlayer vs. sub-monolayer coverage) and the method used for signal normalization (bulk reference vs.

rsf-based). Each model was applied to examine how these assumptions influence the derived coverage values (**Table S5**).

Case 1. The Pt layer was first evaluated using the Cumpson–Seah thickness equation, which assumes a uniform continuous overlayer and expresses the Pt deposition in terms of an effective film thickness rather than atomic coverage.[6]

$$Ratio_{Au}^{Pt} = \frac{I_{Pt}/I_{Pt\ rsf}}{I_{Au}/I_{Au\ rsf}} \quad [2]$$

$$d = \ Lcos\theta \ln[1 + Ratio_{Au}^{Pt}] \quad [3]$$

where d for a complete monolayer is taken to be d_{111} or 0.2266 nm and Pt coverage is then given by $\theta_{Pt} = d/d_{111}$

Case 2. In contrast to Case 1, the second method quantifies Pt coverage directly rather than thickness, using the Shard overlayer expression with bulk-referenced Pt and Au intensities obtained from metallic Pt and thick Au standards.[7]

$$Ratio_{Au}^{Pt} = \frac{I_{Pt} I_{Au,\infty}}{I_{Pt,\infty} I_{Au}} \quad [4]$$

$$\theta_{Pt} = Ratio_{Au}^{Pt} \ \frac{L_{Pt}}{a_{Pt}} cos\theta \quad [5]$$

Case 3. The same coverage equation as in Case 2 was retained, but bulk reference intensities were replaced by rsf-normalized XPS signals to eliminate dependence on external reference samples.

$$Ratio_{Au}^{Pt} = \frac{I_{Pt}/I_{Pt\ rsf}}{I_{Au}/I_{Au\ rsf}} \quad [2]$$

$$\theta_{Pt} = Ratio_{Au}^{Pt} \frac{L_{Pt}}{a_{Pt}} cos\theta \quad [5]$$

Case 4. To describe partial Pt deposition, a sub-monolayer scheme was introduced in which the Pt layer is defined as the coverage of a one monolayer thick island(s) of Pt on Au.

$$I_{Pt} = I_{Pt\ rsf}\theta_{Pt}(1 - e^{-a_{Pt}/L_{Pt}(E_{Pt})}) \quad [6]$$

$$I_{Au} = I_{Au\ rsf}(1 - \theta_{Pt} + \theta_{Pt}e^{-a_{Pt}/L_{Pt}(E_{Pt})}) \quad [7]$$

$$\frac{\frac{I_{Pt}}{I_{Pt\ rsf}}}{\frac{I_{Au}}{I_{Au\ rsf}}} = \frac{\theta_{Pt}\left(1-e^{-a_{Pt}/L_{Pt}(E_{Pt})}\right)}{1-\theta_{Pt}\left(1-e^{-a_{Pt}/L_{Pt}(E_{Au})}\right)} \quad [8]$$

Taking the ratio of the Pt 4f to Au 4f rsf normalized intensities,

$$Ratio_{Au}^{Pt} = \frac{\frac{I_{Pt}}{I_{Pt\ rsf}}}{\frac{I_{Au}}{I_{Au\ rsf}}} = \frac{\theta_{Pt}K_{Pt}^{Pt}}{1-\theta_{Pt}K_{Au}^{Pt}}$$

[9]

where

$$K_i^j = 1 - e^{-a_j/L_j(E_i)} \quad . \quad [10]$$

Solving the equations gives the respective fractional surface coverages

$$\theta_{Pt} = \frac{Ratio_{Au}^{Pt}}{K_{Pt}^{Pt}+Ratio_{Au}^{Pt}K_{Au}^{Pt}} \quad . \quad [11]$$

Case 5. The sub-monolayer framework was then extended to include quantification of the adsorption of the precursor complex $PtCl_4{}^{2-}{}_{ads}$ based on the Pt and Cl coveage.[8,9]

$$I_{Pt} = I_{Pt\ rsf}\theta_{Pt}(1 - e^{-a_{Pt}/L_{Pt}(E_{Pt})}) \qquad [6]$$

$$I_{Cl} = I_{Cl\ rsf}\theta_{Cl}(1 - e^{-a_{Cl}/L_{Cl}(E_{Cl})}) \qquad [12]$$

$$I_{Au} = I_{Au\ rsf}(1 - \theta_{Pt} - \theta_{Cl} + \theta_{Pt}e^{-a_{Pt}/L_{Pt}(E_{Au})} + \theta_{Cl}e^{-a_{Cl}/L_{Cl}(E_{Au})}) \qquad [13]$$

Taking the ratio of the Pt 4f to Au 4f rsf normalized intensities,

$$\frac{\frac{I_{Pt}}{I_{Pt\ rsf}}}{\frac{I_{Au}}{I_{Au\ rsf}}} = \frac{\theta_{Pt}\left(1 - e^{-a_{Pt}/L_{Pt}(E_{Pt})}\right)}{1 - \theta_{Pt}\left(1 - e^{-a_{Pt}/L_{Pt}(E_{Au})}\right) - \theta_{Cl}(1 - e^{-a_{Cl}/L_{Cl}(E_{Au})})} \qquad [14]$$

$$Ratio_{Au}^{Pt} = \frac{\frac{I_{Pt}}{I_{Pt\ rsf}}}{\frac{I_{Au}}{I_{Au\ rsf}}} = \frac{\theta_{Pt}K_{Pt}^{Pt}}{1 - \theta_{Pt}K_{Au}^{Pt} - \theta_{Cl}K_{Au}^{Cl}}$$

[15]

With similar development the $Ratio_{Au}^{Cl}$ can be determined

and likewise for each species

$$K_i^j = 1 - e^{-a_j/L_j(E_i)} \qquad . \qquad [10]$$

Evaluating for θ_{Pt} and θ_{Cl} gives the respective equations

$$\theta_{Cl} = \frac{[Ratio_{Au}^{Cl}]K_{Pt}^{Pt}}{K_{Cl}^{Cl}K_{Pt}^{Pt} + [Ratio_{Au}^{Cl}]K_{Au}^{Cl}K_{Pt}^{Pt} + [Ratio_{Au}^{Pt}]K_{Au}^{Pt}} \qquad [16]$$

$$\theta_{Pt} = \frac{[Ratio_{Au}^{Pt}]\ (1-\theta_{Cl}K_{Au}^{Cl})}{K_{Pt}^{Pt}+[Ratio_{Au}^{Pt}]K_{Au}^{Pt}} \quad . \qquad [17]$$

Case 6. The same co-adsorption equation used in **Case 5** was retained but evaluated with the θ_{Cl} ratio constrained to the stoichiometric value of $PtCl_4$.

$$Ratio_{Au}^{Pt} = \frac{\frac{I_{Pt}}{I_{Pt\ rsf}}}{\frac{I_{Au}}{I_{Au\ rsf}}} = \frac{\theta_{Pt}K_{Pt}^{Pt}}{1-\theta_{Pt}K_{Au}^{Pt}-\theta_{Cl}K_{Au}^{Cl}}$$

[15]

$$\theta_{Pt} = \frac{[Ratio_{Au}^{Pt}]\ (1-\theta_{Cl}K_{Au}^{Cl})}{K_{Pt}^{Pt}+[Ratio_{Au}^{Pt}]K_{Au}^{Pt}} \ ,\ and \quad \theta_{Cl} = 4\theta_{Pt}$$

[18]

$$\theta_{Pt} = \frac{[Ratio_{Au}^{Pt}]}{K_{Pt}^{Pt}+[Ratio_{Au}^{Pt}](K_{Au}^{Pt}+4K_{Au}^{Cl})} \qquad [19]$$

(a)

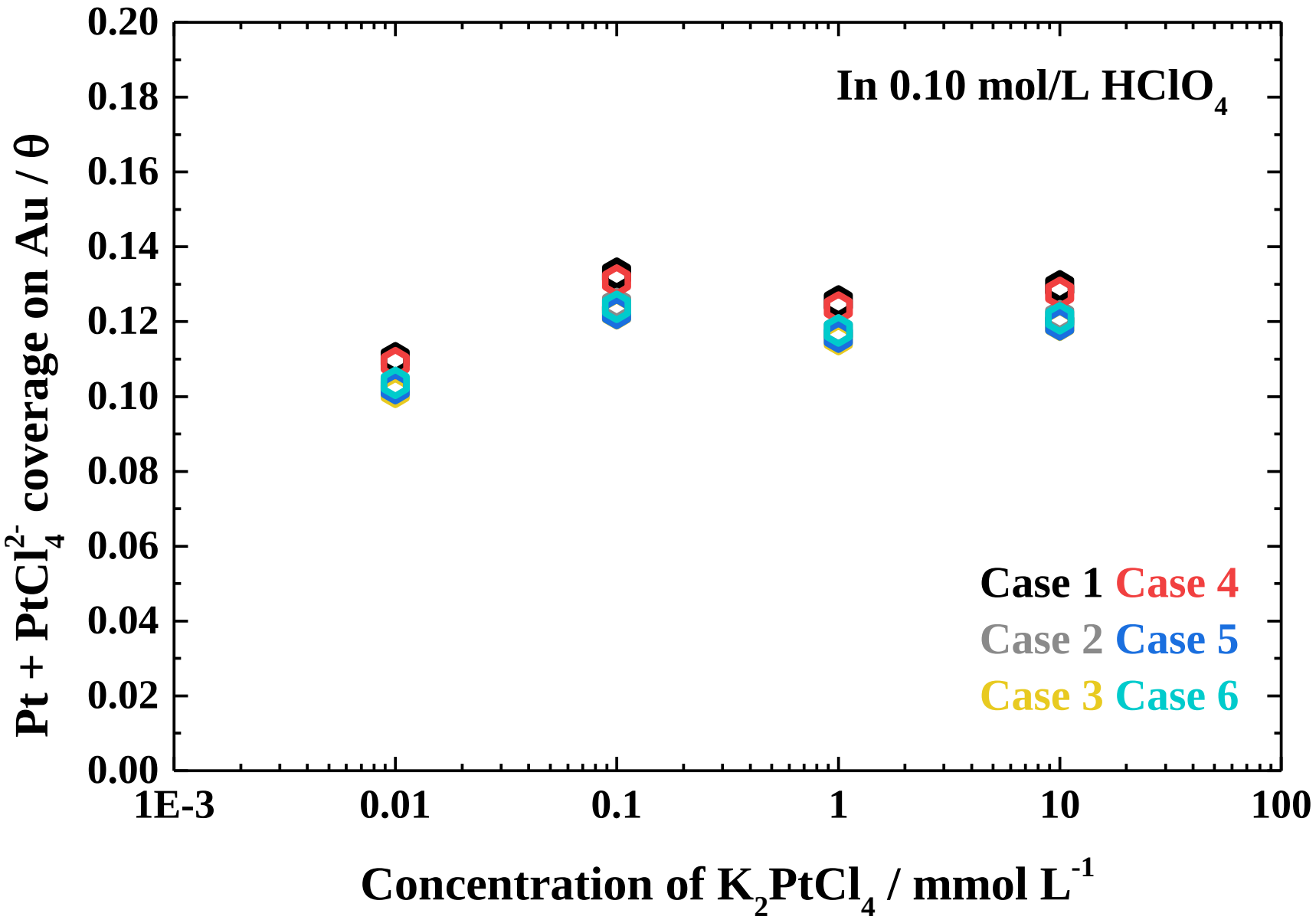

(b)

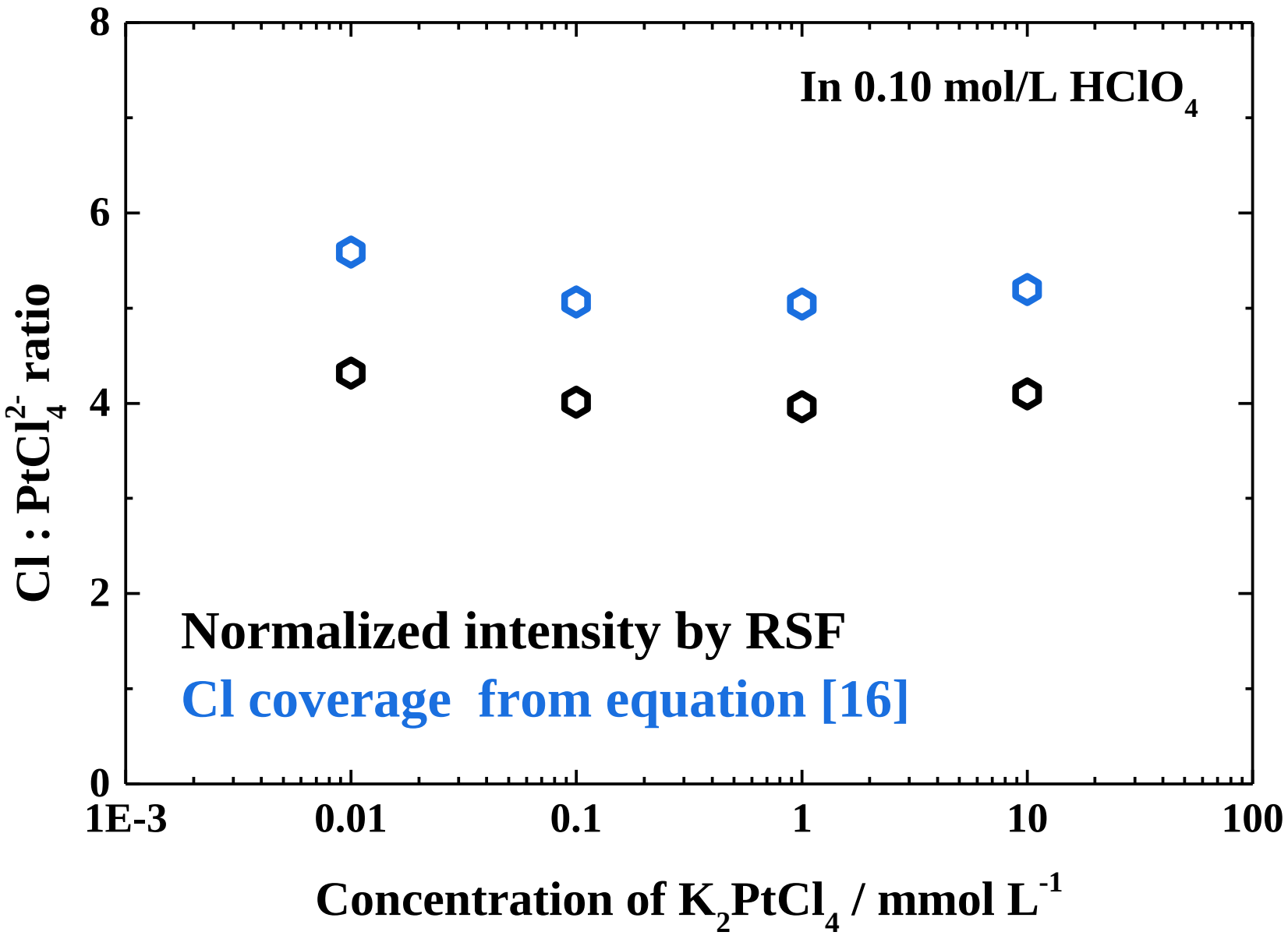

Fig. S9 (a) $PtCl_4^{2-}{}_{ads}$ coverage on Au (111) as a function of K_2PtCl_4 and coverage model. (b) Cl/Pt ratio depending on calculation method.

To quantify the surface coverage of adsorbed $PtCl_4{}^{2-}{}_{ads}$ species, a series of different models were evaluated using XPS data. For model evaluation the data from the nominally "Cl^- free" supporting electrolyte system, x mol/L K_2PtCl_4 in 0.1 mol/L $HClO_4$, was employed. As indicated in **Fig. S9a**, for the given dataset the six models show no significant difference in the calculated $PtCl_4{}^{2-}{}_{ads}$ coverage on Au.

Case 5, in particular, enables the determination of θ_{Cl} and θ_{Pt}, from which a Cl/Pt atomic ratio can be derived. However, the calculated ratios (5.04 to 5.60) substantially exceeded the stoichiometry of $PtCl_4$, indicating either a systematic overestimation or alternatively, speak to the presence of excess halide that might be consistent with adlayer formation on terraces other than (111)[10] (**Fig. S9b**). In contrast, a simplified approach based only on the rsfs ($Ratio_{Pt}^{Cl}$) the Cl/Pt ratio is between 3.96 and 4.31 congruent with $PtCl_4{}^{2-}$ stoichiometry. This difference arises because the Cl/Pt ratios in **Case 5** also include the terms for K_{Pt}^{Pt} and K_{Cl}^{Cl}, i.e., $Ratio_{Pt}^{Cl}\ \frac{K_{Pt}^{Pt}}{K_{Cl}^{Cl}}$. Importantly the different approach used to calculate the Cl/Pt ratio does not significantly affect the extracted values for $PtCl_4{}^{2-}{}_{ads}$ coverage. Given the simple alignment with the adsorbed complex stoichiometry, the simpler method based on $Ratio_{Pt}^{Cl}$ is reported in the main text (**Fig. 2c**,**2h, and 3f**).

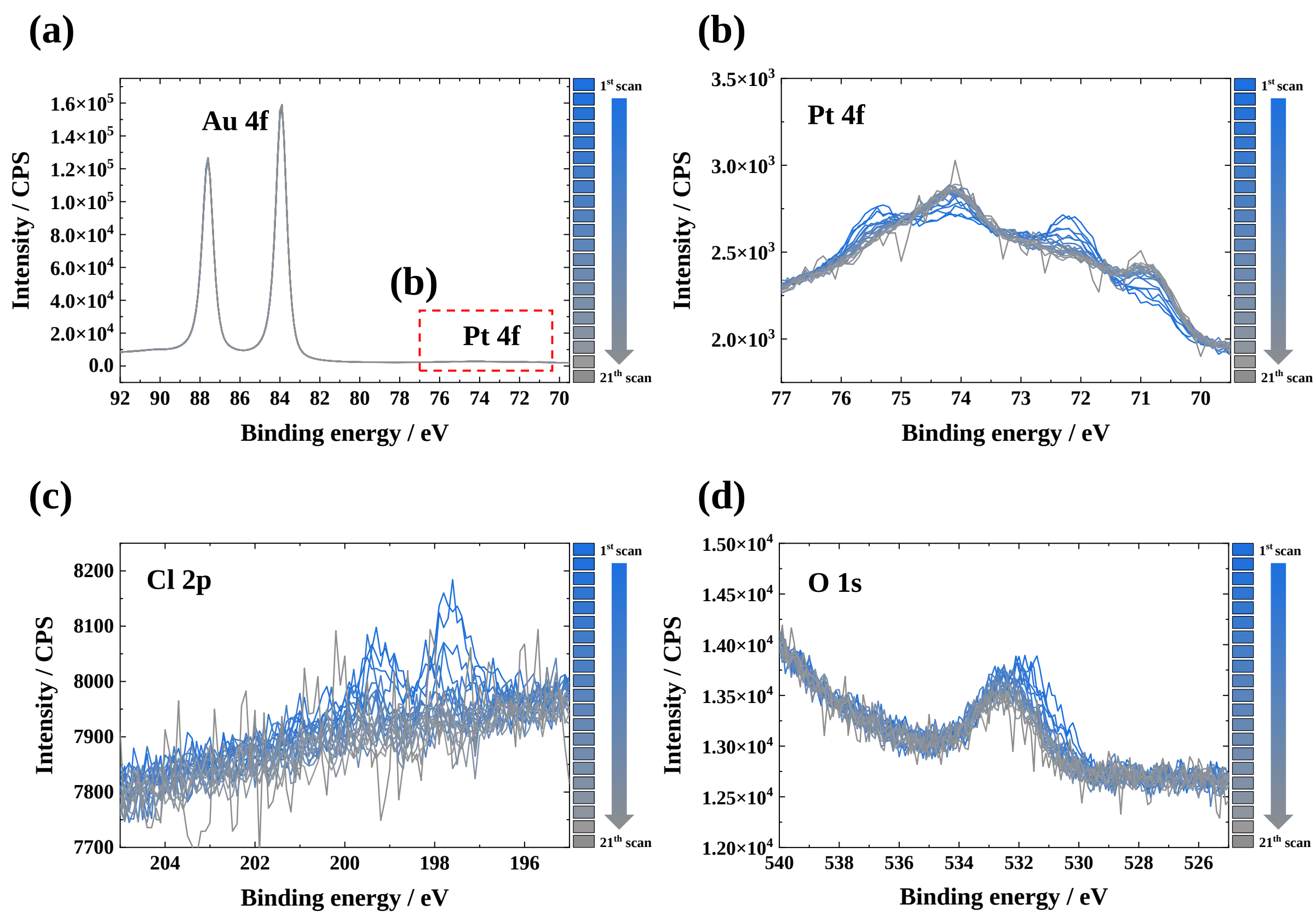

Fig. S10 Twenty-one serial scans of (a) Au 4f, (b) Pt 4f, (c) Cl 2p, and (d) O 1s XPS spectra reveal photoreduction of $PtCl_4^{2-}{}_{ads}$ on Au. Specimens were prepared by 100 s exposure at 0.40 V_{SSCE} in 0.5 mol/L NaCl with 0.01 mmol/L K_2PtCl_4.

To investigate XPS photoreduction of $PtCl_4^{2-}{}_{ads}$, 21 sequential scans were acquired on $PtCl_4^{2-}{}_{ads}$/Au under conventional grounding conditions. In the initial scan, the Pt $4f_{7/2}$ peak was at 72.2 eV corresponding to $PtCl_4^{2-}{}_{ads}$. With an increasing number of scans, this peak decreased monotonically while the peak at 70.9 eV for metallic Pt grew in intensity. Complete conversion to Pt metal was observed by the 10th scan, accompanied by the disappearance of the Cl 2p signal, consistent with full photoreduction. For all other XPS measurements presented in the main text, spectra were acquired by averaging 12 scans over the Pt 4f region (including Au 4f),

20 scans for Cl 2p, 5 scans each for O 1s and C 1s, and 3 survey scans with the possibility of partial photoreduction of $PtCl_4{}^{2-}{}_{ads}$ not being entirely excluded.

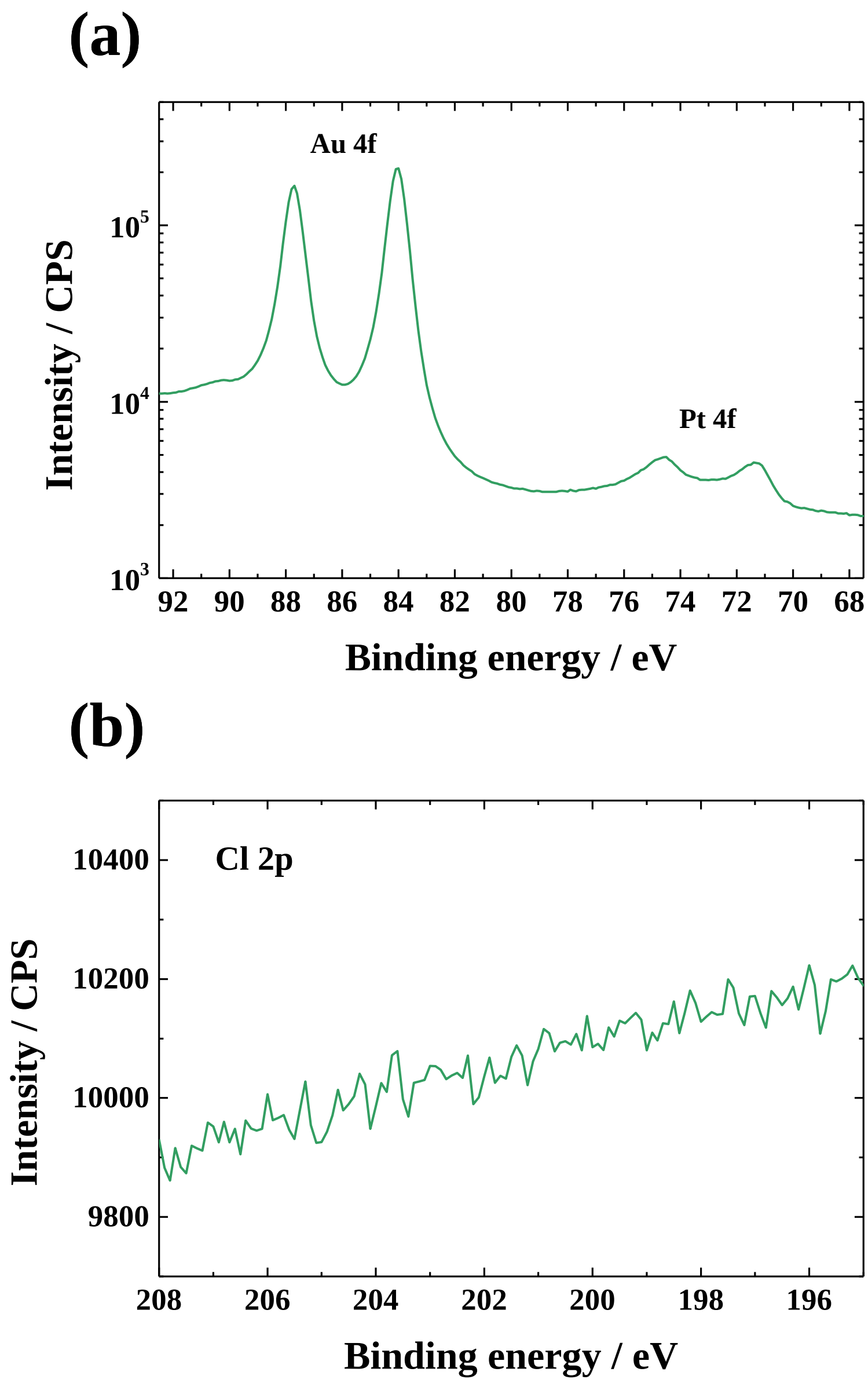

Fig. S11 XPS spectra of the Pt/Au mica surface following reduction of pre-adsorbed $PtCl_4^{2-}$ adlayer, corresponding to the STM measurements in **Fig. 3b.** (a) Au 4f and Pt 4f regions and (b) Cl 2p region.

STM Imaging of Aged $PtCl_4^{2-}{}_{ads}$ Derivatized Au

Imaging the $PtCl_4^{2-}{}_{ads}$ derivatized specimen (**Fig. 3)** after aging in an Ar atmosphere for 21 hours revealed a stripped structure at the step edges or perhaps "magic fingers **Fig. S12**,."[11-13] Several examples detailing the development magic fingers with imaging have been reported in both vacuum and ambient environments although mechanistic understanding remains unresolved. In the present system the lower surface energy and high relative mobility of Au atoms, relative to Pt, facilitate surface segregation with steps being the most obvious source of Au adatom and site for reorganization. First principles studies of Pt-Au surface alloy formation indicate laterally ordered striped phases can be more stable than Au or Pt capped Pt surface. That said, stabilization energy is modest on the order of 10 meV/surface atom indicating a delicate process is required to realize such structures. Further work will be needed to better understand the nature of the spontaneous Pt reduction process and the dynamics at Pt decorated steps.

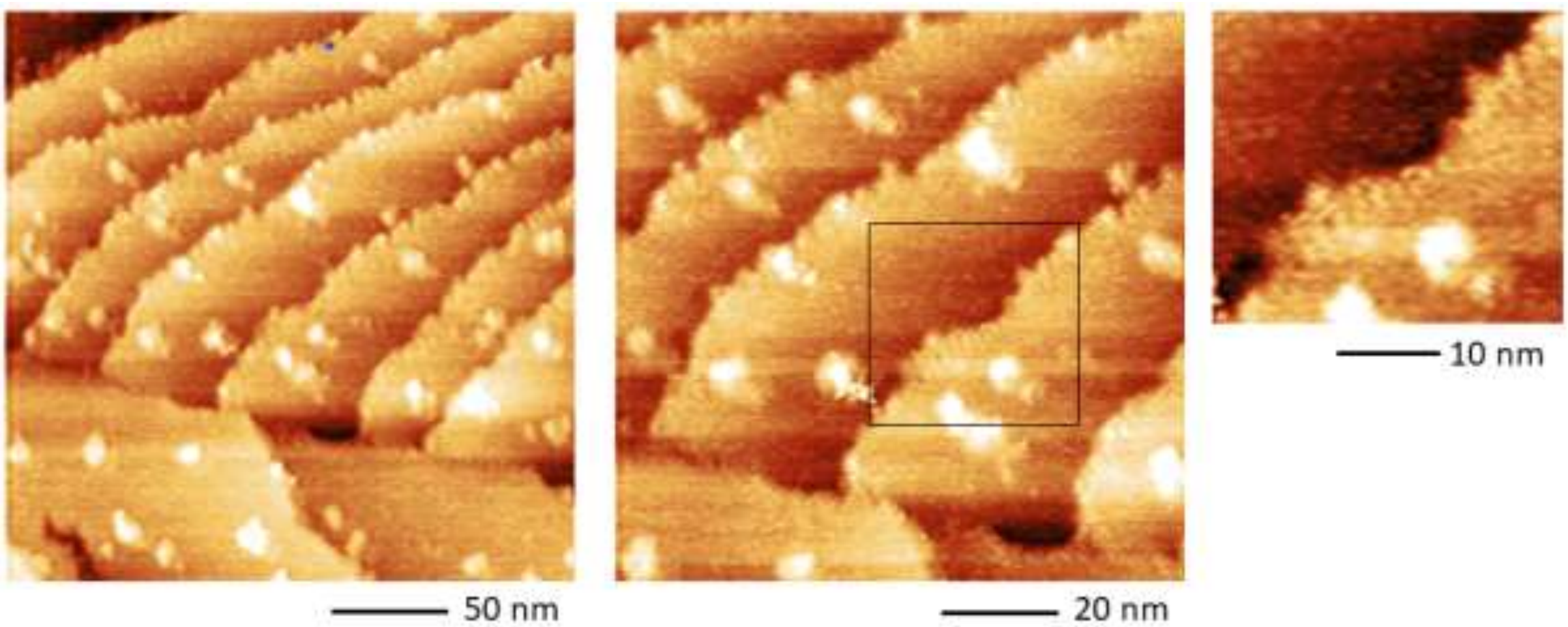

Fig. S12 Conversion of Pt decorated Au steps into fingers or a striped phase after aging 21 hours in Ar. The ex-situ images in an Ar environment with tunneling current of 1.73 nA and specimen bias of +0.1 V.

Pt(IV) versus Pt(II) Precursors

As in many prior studies of Pt electrodeposition employed Pt(IV) precursors, a limited set of experiments was also conducted using K_2PtCl_6 under the same conditions as were used above for K_2PtCl_4. Following 100 s dwell at +0.40 V_{SSCE}, a muti-component reduction wave is observed between +0.20 V_{SSCE} and 0 V_{SSCE} for K_2PtCl_6 in 0.5 mol/L NaCl (**Fig. S13a**). The peak current is at least twice the size of that for $PtCl_4{}^{2-}{}_{aq}$ consistent with a 4e$^-$ reduction of $PtCl_6{}^{2-}{}_{aq}$ to Pt. Further still, the well-defined, sharp reduction peak is absent during the second voltammetry sweep congruent with a loss of surface selective reduction like that observed for the divalent precursor (**Fig. S13b**). The residual current on the second cycle along with the sustained current down to -0.20 V_{SSCE} (**Fig. S13c**) is ascribed to 2e$^-$ reduction of $PtCl_6{}^{2-}$ to $PtCl_4{}^{2-}$ with the liberation of two chloride ions.

The difference between the two cycles reflects a change in the reactions occurring on mixed Au-Pt surface versus a Pt covered electrode. The onset of bulk Pt deposition occurs below -0.20 V_{SSCE} reaching a peak current near -0.305 V_{SSCE} that is twice that observed for reduction of the divalent precursor consistent with sequential 2e$^-$ reduction reactions $PtCl_6{}^{2-}$ to $PtCl_4{}^{2-}$ followed by $PtCl_4{}^{2-}$ to Pt. At still more negative potentials, metal deposition is quenched by formation of a saturated layer of H_{ads} that blocks $PtCl_4{}^{2-}$ adsorption while still supporting the reduction of $PtCl_6{}^{2-}$ to $PtCl_4{}^{2-}$ protons to H_2 and eventually water to H_2.

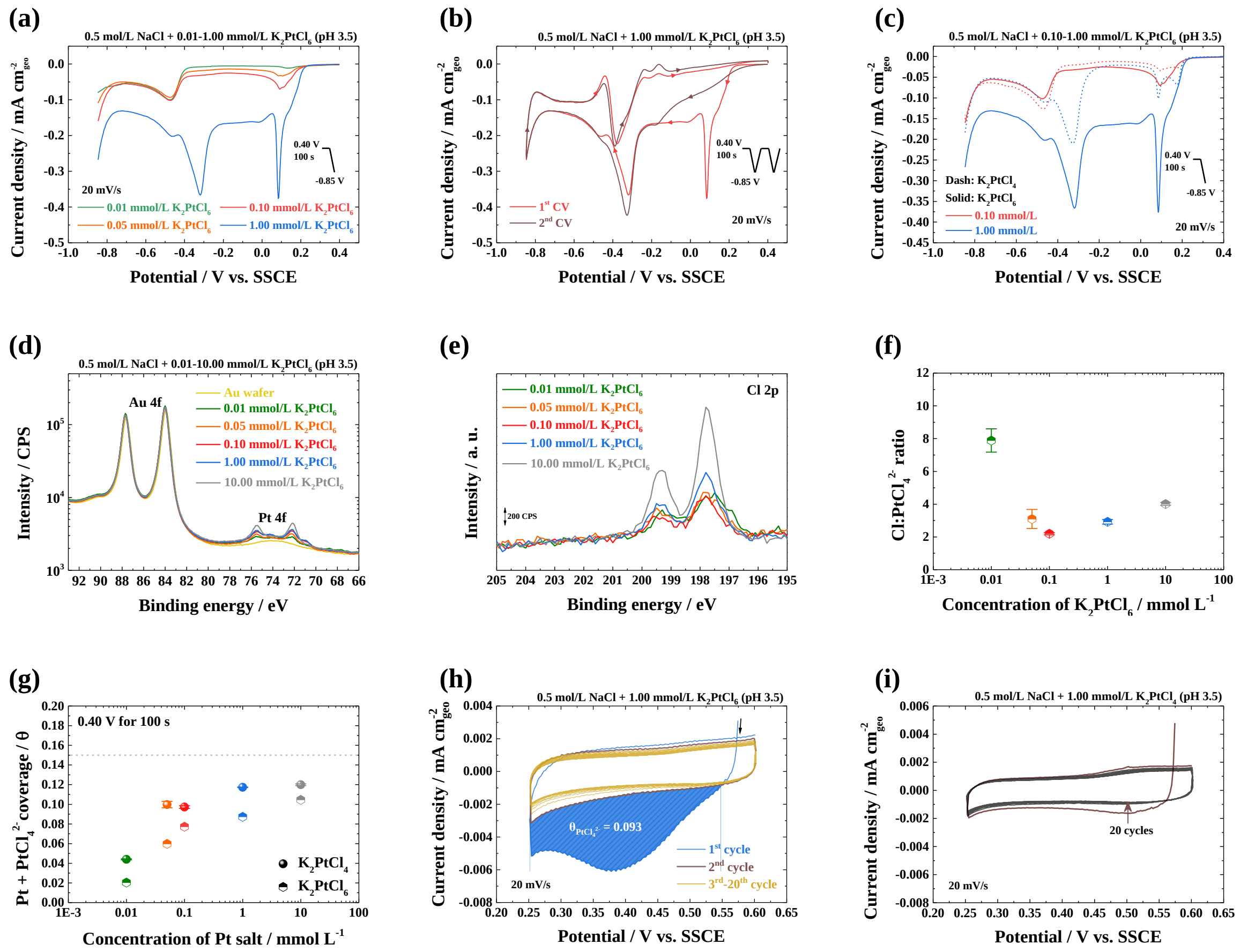

Fig. S13 Electroanalytical and XPS analysis of adsorption and reduction of $PtCl_6^{2-}{}_{aq}$. (a) Linear scan voltammetry in 0.5 mol/L NaCl containing (0.01 to 10.0) mmol/L K_2PtCl_6 initiated after 100 s dwell at 0.40 V_{SSCE}. (b) Two consecutive voltammetry cycles in 0.5 mol/L NaCl containing 1.00 mmol/L K_2PtCl_6. (c) Comparison of linear scan recorded in 0.10 mmol/L or 1.00 mmol/L K_2PtCl_4 and K_2PtCl_6, see **Fig. S14** for 10.0 mmol/L K_2PtCl_4 and K_2PtCl_6. (d-e) Pt 4f and Cl 2p spectra of adsorbed Pt–Cl species derivatized in 0.5 mol/L NaCl containing (0.01 to 10.0) mmol/L K_2PtCl_6. (f) Cl/$PtCl_4^{2-}$ ratio depends on the K_2PtCl_6 concentration. (g) XPS-derived adsorbed coverage of $PtCl_4^{2-}$ depends on the K_2PtCl_4 and K_2PtCl_6 concentration. (h,I) Twenty consecutive voltammetry cycles initiated immediately following immersion of Au in 0.5 mol/L NaCl containing (h) 1.00 mmol/L K_2PtCl_6 and (i) 1.00 mmol/L K_2PtCl_4. Error bars in (f) indicate

the standard deviation from three positions on a single specimen (uniformity); in (g), from repeated experiments (reproducibility).

XPS was used to examine the electrode surface following treatment for 100 s at +0.40 V_{SSCE}. The dominant Pt $4f_{7/2}$ peak at 72.2 eV is consistent with divalent $PtCl_4^{2-}{}_{ads}$ and distinct from that for the tetravalent salt (**Fig. S13d** and **Fig. S6**). The smaller adjacent low binding energy peak aligns with the metallic state and is attributed to X-ray photoreduction during XPS consistent with literature observations detailed earlier (**Fig. S13**)**.** For K_2PtCl_6 concentrations greater than 0.05 mmol/L, the sensitivity factor normalized Cl 2p/Pt 4f ratio for the square planar divalent adsorbate $(PtCl_x(H_2O)_{4-x})^{(x-2)-}{}_{ads}$ varies between 2 and 4 depending on the precursor concentration (**Fig. S13e** and **Fig. S13f**). Compared to starting with the divalent precursor, XPS analysis indicates a slightly lower $PtCl_4^{2-}{}_{ads}$ saturation coverage of 0.105 for the K_2PtCl_6 electrolyte indicative of hindered assembly during reductive adsorption (**Fig. S13g**).

Voltammetry between +0.60 V_{SSCE} and +0.25 V_{SSCE} initiated immediately upon immersion at +0.575 V_{SSCE} reveals a wave for $PtCl_6^{2-}{}_{aq}$ reduction to $PtCl_3(H_2O)^-{}_{ads}$, or $PtCl_4^{2-}{}_{ads}$ near +0.380 V_{SSCE} and no corresponding oxidation process on the return sweep (**Fig. S13h**). Importantly, the reduction peak is absent on subsequent voltammetric cycles, consistent with irreversible adsorption of $PtCl_3(H_2O)_1^-{}_{ads}$ or $PtCl_4^{2-}{}_{ads}$ blocking further ion transfer adsorption reactions. The net background corrected charge, the shaded region in **Fig. S13h**, is equivalent

t

o

a

c

experiment is performed with the K_2PtCl_4 electrolyte (**Fig. S13i**). The modest excess voltammetric charge in this case is attributed to reduction of transient Pt(IV) species generated by slow oxidation of $PtCl_4{}^{2-}{}_{aq}$ during the immersion step at +0.575 V_{SSCE}.

Summarizing the principal findings for the Cl^--rich electrolytes in **Fig. S13,** the initial electroreduction of $PtCl_6{}^{2-}$ to Pt proceeds through a divalent $PtCl_3(H_2O)^-{}_{ads}$ or $PtCl_4{}^{2-}{}_{ads}$ adsorbed intermediate.

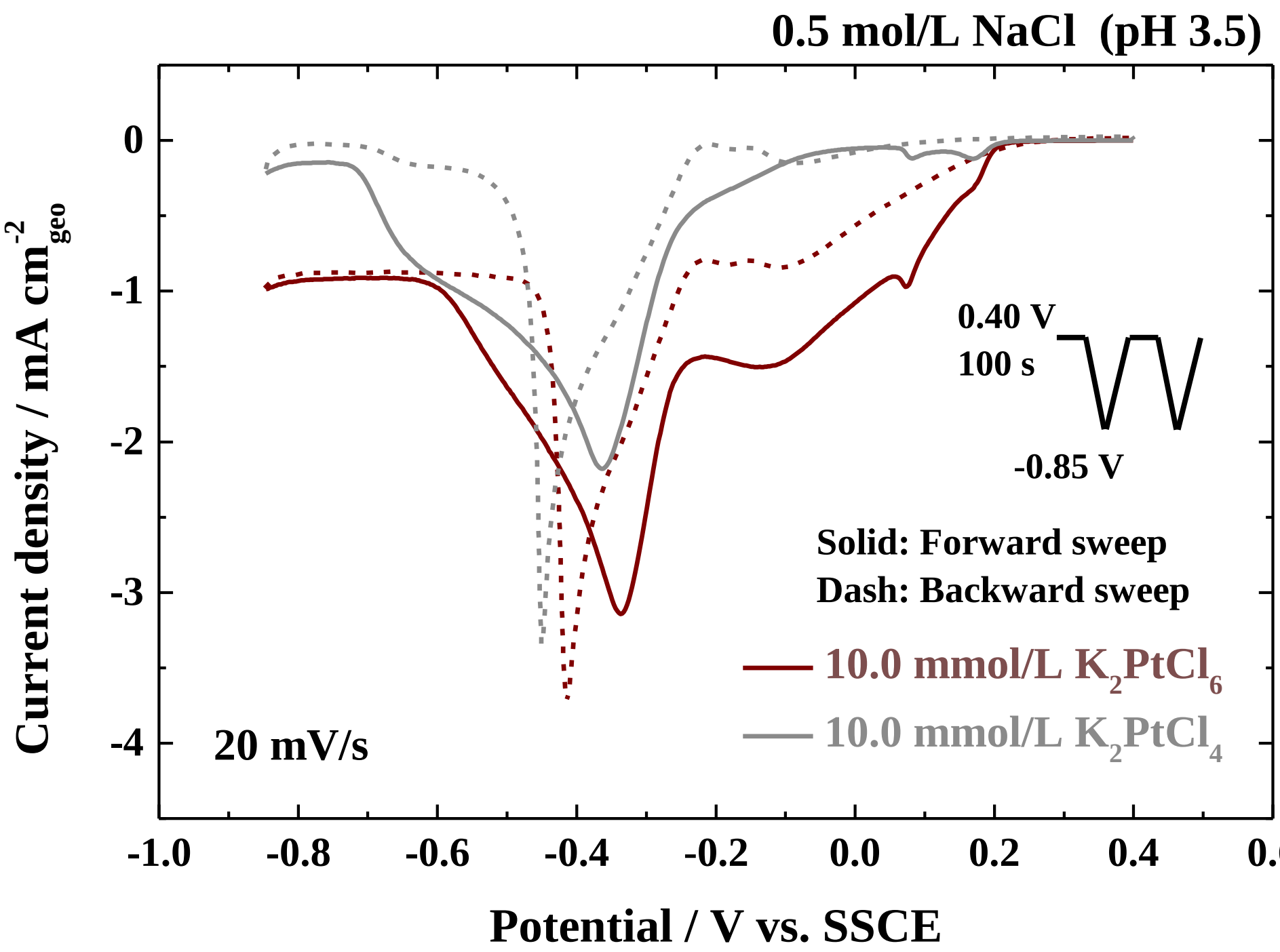

Fig. S14 Comparison of linear scan recorded in 0.5 mol/L NaCl with 10.0 mmol/L K_2PtCl_4 and K_2PtCl_6. Linear scan initiated after 100 s dwell at 0.40 V_{SSCE} with a scan rate of 20 mV/s.

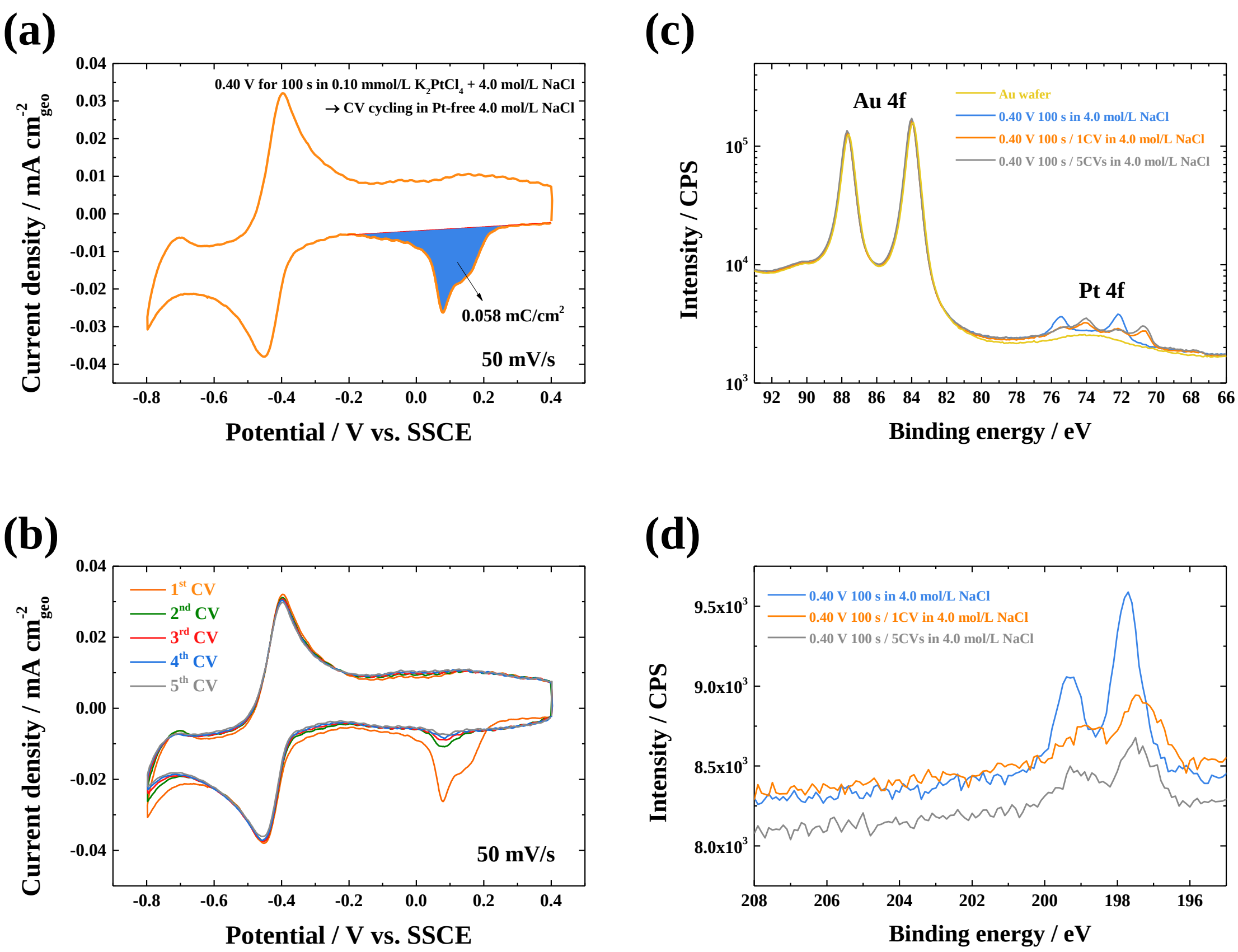

Fig. S15 Two-step Pt deposition process with $PtCl_4{}^{2-}{}_{ads}$ derivatization in 0.10 mmol/L K_2PtCl_4 and 4.0 mol/L NaCl is followed by reduction in a Pt-free 4.0 mol/L NaCl electrolyte. (a) First voltammetric reduction scan for reduction of $PtCl_4{}^{2-}{}_{ads}$ on Au in 4.0 mol/L NaCl. (b) Five sequential voltammogram of $PtCl_4{}^{2-}{}_{ads}$ on Au in 4.0 mol/L NaCl. (c,d) Pt 4f and Cl 2p spectra after $PtCl_4{}^{2-}{}_{ads}$ derivatization in 4.0 mol/L NaCl and following reduction in Pt-free 4.0 mol/L NaCl.

A two-step process was adopted where the Au electrode was first derivatized with $PtCl_4{}^{2-}{}_{ads}$ at +0.40 V_{SSCE} for 100 s in 0.10 mol/L K_2PtCl_4 and 4.0 mol/L NaCl and then transferred to 4.0 mol/L NaCl where the adsorbed complex was potentiodynamically reduced. The voltammetric reduction wave between 0.20 V_{SSCE} and -0.10 V_{SSCE} in **Fig. S15** has two components and integration using a linear background yields a charge of 0.058 mC/cm^2 that if assigned entirely to $PtCl_4{}^{2-}{}_{ads}$ reduction would correspond to a coverage of 0.12. In contrast, prior XPS measurements (**Fig. 2e)** indicate the initial $PtCl_4{}^{2-}{}_{ads}$ coverage is 0.079. Following one reduction cycle in fresh 4.0 mol/L NaCl and emersion at 0.40 V_{SSCE} the values are 0.036 monolayer of Pt metal and a 0.029 monolayer of $PtCl_4{}^{2-}{}_{ads}$ on Au. After five successive cycles in fresh 4.0 mol/L NaCl, the coverage of Pt metal and residual $PtCl_4{}^{2-}{}_{ads}$ remained nearly unchanged at 0.038 and 0.022 monolayers, respectively. Consistent with this finding, voltammetry shows a sharp fall-off in the peak reduction current after the first cycle with only a minor residual wave evident on the fifth cycle. Congruent with this, the reduction step is only accompanied by a partial decrease in the Cl 2p signal (**Fig. S15).**

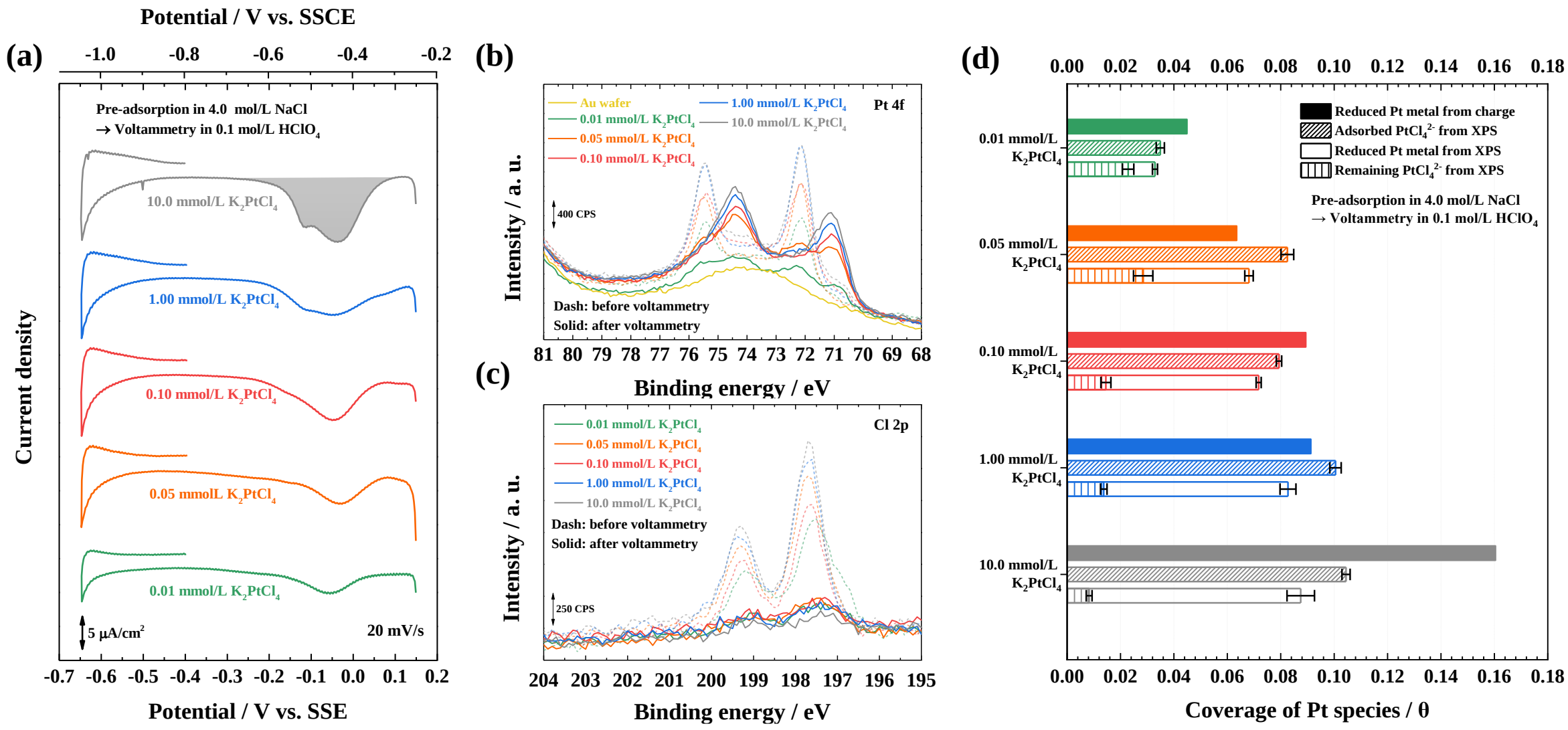

Fig. S16 Two-step Pt deposition process with $PtCl_4^{2-}{}_{ads}$ derivatization in 0.10 mmol/L K_2PtCl_4 and 4.0 mol/L NaCl is followed by reduction in a Pt-free 4.0 mol/L NaCl electrolyte. (a) First voltammetric reduction scan for reduction of $PtCl_4^{2-}{}_{ads}$ on Au in 4.0 mol/L NaCl. (b) Five sequential voltammogram of $PtCl_4^{2-}{}_{ads}$ on Au in 4.0 mol/L NaCl. (c,d) Pt 4f and Cl 2p spectra after $PtCl_4^{2-}{}_{ads}$ derivatization in 4.0 mol/L NaCl and following reduction in Pt-free 4.0 mol/L NaCl.

To examine the influence of the supporting electrolyte in the derivatization and reduction step, experiments are performed where 0.1 mol/L $HClO_4$ alternately replaced by 4.0 mol/L NaCl. In the first series, $PtCl_4^{2-}{}_{aq}$ is adsorbed at 0.40 V_{SSCE} for 100 s from an electrolyte comprised of (0.01 to 10.00) mmol/L K_2PtCl_4 dissolved in 4.0 mol/L NaCl. Following emersion and extensive rinsing with Ar-purged dilute HCl (pH 3.5), the electrode is immersed in 0.1 mol/L $HClO_4$ where the adsorbed complex is reduced voltammetrically **(Fig. S16)**. The scan begins with a spike of uncertain origins followed by a reduction peak near -0.060 V_{SSE} (-0.458

V_{SSCE}) whose charge reflects the resulting $PtCl_4^{2-}{}_{ads}$ coverage. For derivatization in solutions with less than 0.10 mmol/L K_2PtCl_4, reduction is characterized by a single peak with an asymmetric tail extending to more negative potentials. For higher K_2PtCl_4 concentrations, a second reduction peak is evident near -0.120 V_{SSE} (-0.518 V_{SSCE}). The peak charge, integrated between 0.10 V_{SSE} and -0.40 V_{SSE} (**Fig. S16d**), is in good agreement with the $PtCl_4^{2-}$ coverage reported in **Fig. 2e** with the exception of results for 10 mmol/L K_2PtCl_4.

The binding energy and intensity ratio of the Pt 4f and Cl 2p spectra for the derivatized electrode are congruent with $PtCl_4^{2-}{}_{ads}$ and the coverage scales with K_2PtCl_4 concentration as captured in **Fig. 2**. Following potentiodynamic reduction of the $PtCl_4^{2-}{}_{ads}$ the derivatized electrodes were emersed at -0.40 V_{SSE} (**Fig. S16a**) and transferred to the XPS spectrometer (**Fig. S16b, S16c)**. The shift of the Pt 4f to lower binding energy and the loss of Cl 2p intensity align with the reduction of $PtCl_4^{2-}{}_{ads}$ to Pt. Comparing the Pt 4f/Au 4f envelope before and after reduction, albeit for distinct specimens, indicates that the total amount of surface bound Pt is largely conserved during the two-step process; the Pt 4f envelope is asymmetric with enhanced intensity at higher binding energies consistent with some combination of residual $PtCl_4^{2-}$ and oxidization of freshly formed Pt during transfer to the spectrometer.

Interestingly, comparison of Pt 4f and O 1s signals, normalized by relative sensitivity factors, reveals minimal change from the reduction. However, the Cl 2p/$PtCl_4^{2-}$ intensity ratio scatters between 4 and 3 following the reduction step (**Fig. S17**), suggesting only partial reduction of $PtCl_4^{2-}$ but also, perhaps oxidation and/or finite size effects for small Pt islands or adatoms during the emersion process. The Pt surface coverage during these experiments is

summarized in **Fig. S16** and demonstrates that the two-step process is suitable for isolating irreversible surface-selective $PtCl_4{}^{2-}{}_{ads}$ adsorption from the subsequent reduction step.

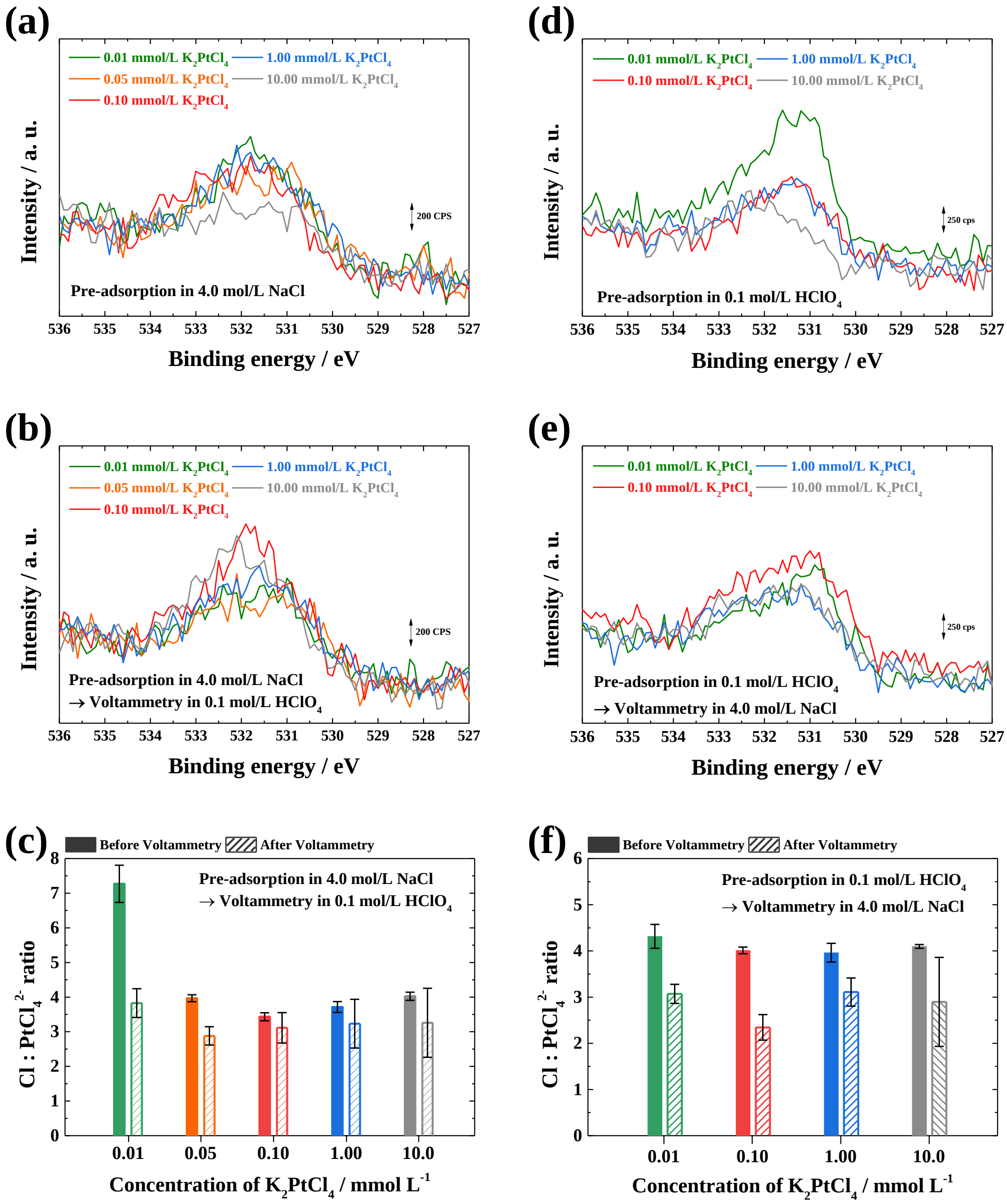

Fig. S17 XPS analysis of the O 1s spectra for $PtCl_4^{2-}{}_{ads}$ derivatized Au electrodes (a, d) before and after (b,e) voltammetric reduction in Pt-free electrolytes as detailed in **Figure S16 and Figure 4**. (c) Comparison of the Cl 2p to $PtCl_4^{2-}{}_{ads}$ intensity ratio before and after voltammetric reduction, as a function of K_2PtCl_4 concentration used in the derivatization in 4.0 mol/L NaCl followed by reduction in 0.1 mol/L $HClO_4$. (f) Comparison of the Cl 2p to $PtCl_4^{2-}{}_{ads}$ intensity

ratio before and after reduction, as a function of K_2PtCl_4 concentration used for derivatization in 0.1 mol/L $HClO_4$ followed by reduction in 4.0 mol/L NaCl.

As described in the main text, all derivatization experiments in Cl^--free $HClO_4$ electrolyte were completed within 16 min of salt dissolution, ensuring that $PtCl_4^{2-}$ remained the dominant Pt species. More specifically, for XPS specimens acquired prior to reduction, 100 s derivatization occurred 6 min after adding 0.01 mmol/L and 0.10 mmol/L K_2PtCl_4, and 2 min after adding 1.00 mmol/L and 10.0 mmol/L K_2PtCl_4 to the electrolyte. For specimens that were analyzed after reduction, derivatization for 100 s was conducted after 16 min (0.01 mmol/L and 0.10 mmol/L) or 12 min (1.00 mmol/L and 10.0 mmol/L) after adding the K_2PtCl_4.

(a)

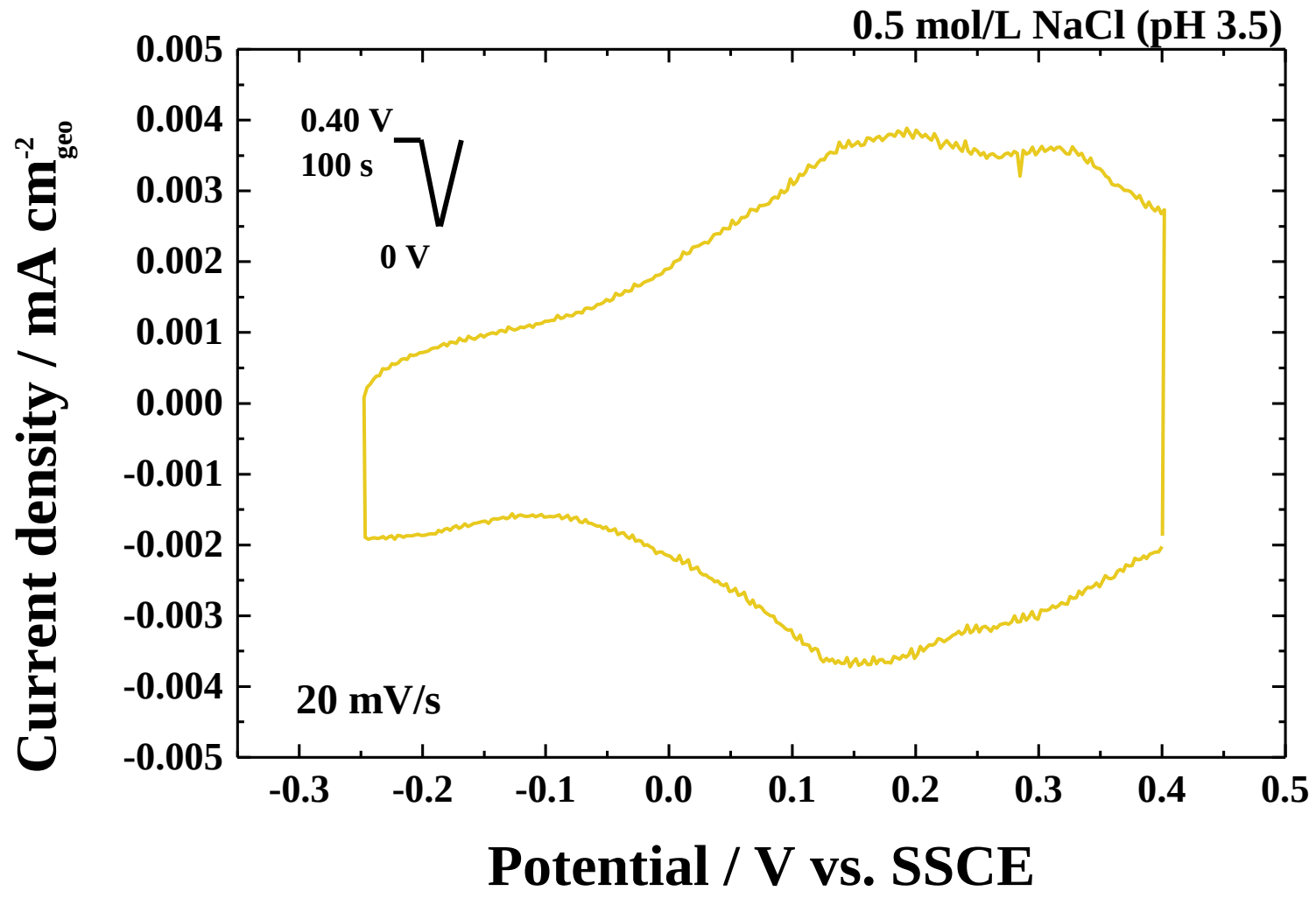

(b)

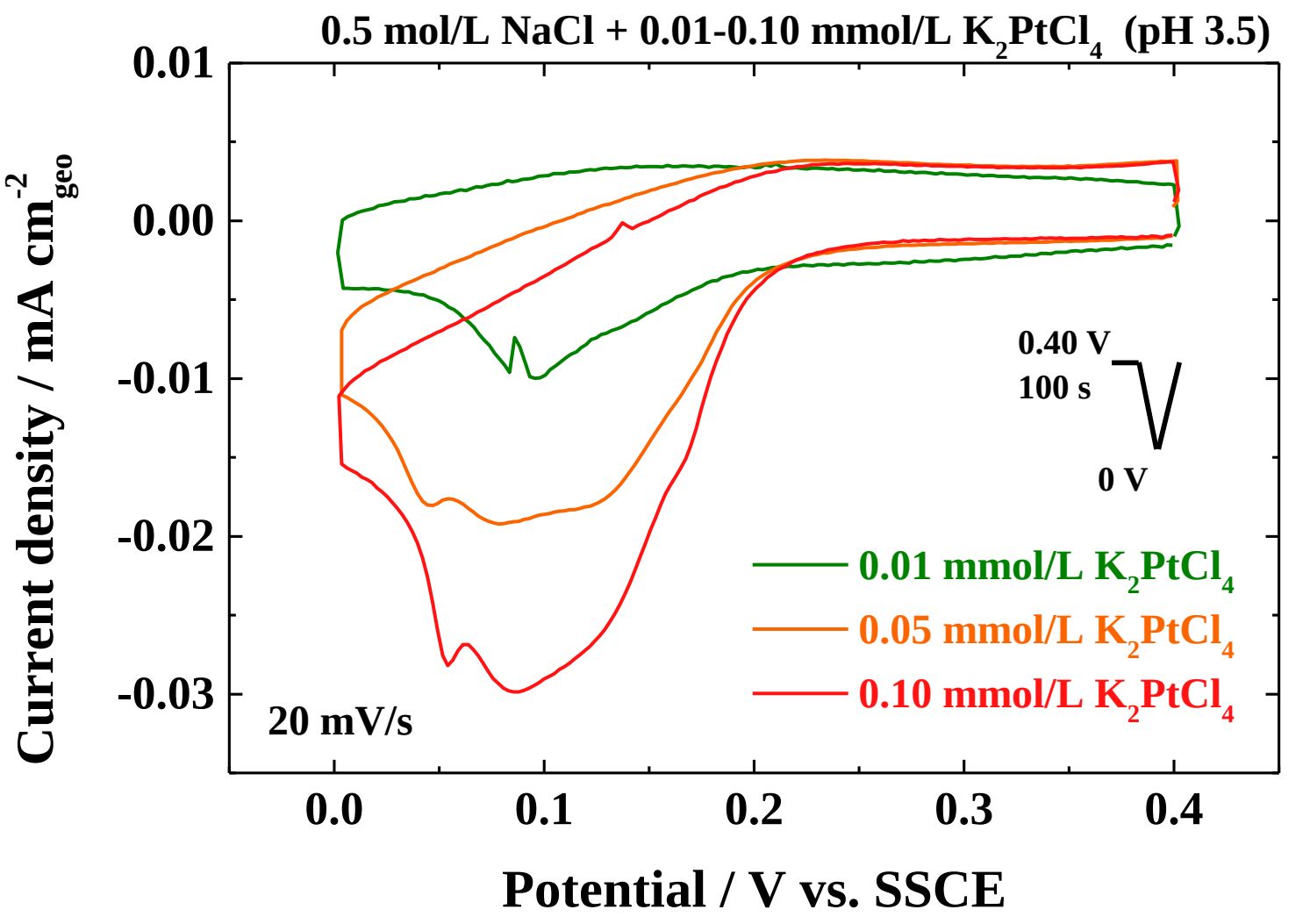

Fig. S18 (a) Cyclic voltammetry of Au wafer after a 100 s dwell at 0.40 V_{SSCE} in Pt free 0.5 mol/L NaCl. (b) Voltammetry after 100 s dwell at 0.40 V_{SSCE} as a function of K_2PtCl_4 concentration in 4.0 mol/L NaCl electrolyte. This figure presents an expanded view of the data presented in **Figure 5a** to highlight the voltammetric features.

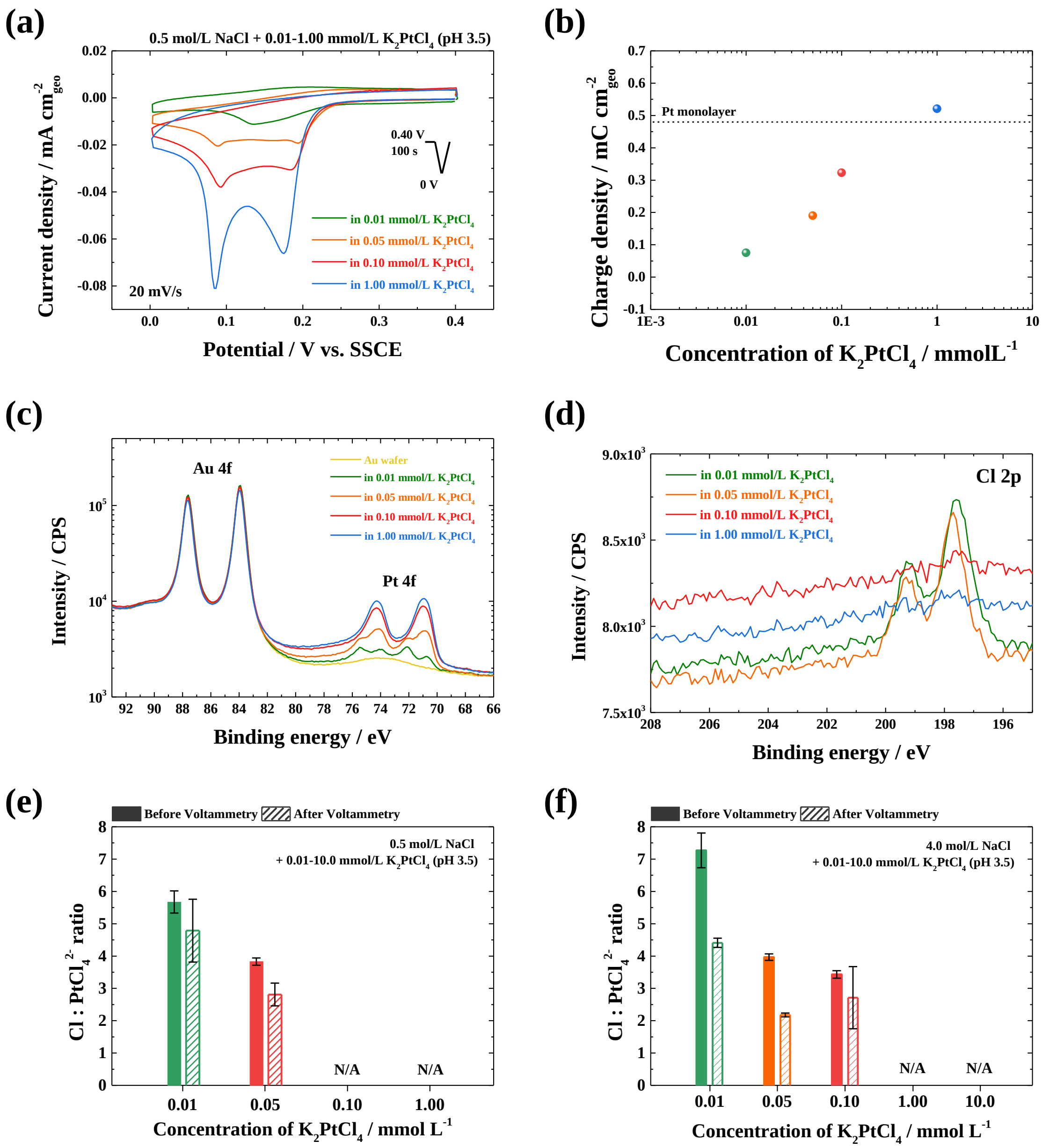

Fig. S19 (a) Cyclic voltammetry at 20 mV/s of Au wafer initiated after a 100 s dwell at 0.40 V_{SSCE} as a function of K_2PtCl_4 concentration in 0.5 mol/L NaCl electrolyte. (b) Net voltammetric reduction charge in (a). (c,d) Pt 4f and Cl 2p spectra following one voltammetric cycle in 0.5 mol/L NaCl electrolyte. The parameters shown in **Figure 5b,e** were derived from these spectra. Comparison of the Cl 2p to $PtCl_4^{2-}{}_{ads}$ intensity ratio before and after voltammetric reduction performed (e) in 0.5 mol/L NaCl and (f) 4.0 mol/L NaCl as a function of K_2PtCl_4 concentration.

Given the rsf of Cl 2p ($S_{Cl\ 2p}$ = 0.891) quantitative analysis of the Cl 2p signal at high Pt coverage would be unreliable. For this reason, the Cl 2p to $PtCl_4^{2-}{}_{ads}$ intensity ratio was not determined for the samples grown in 0.10 mmol/L and 1.00 mmol/L K_2PtCl_4 and summarized in Figure (e) and (f).

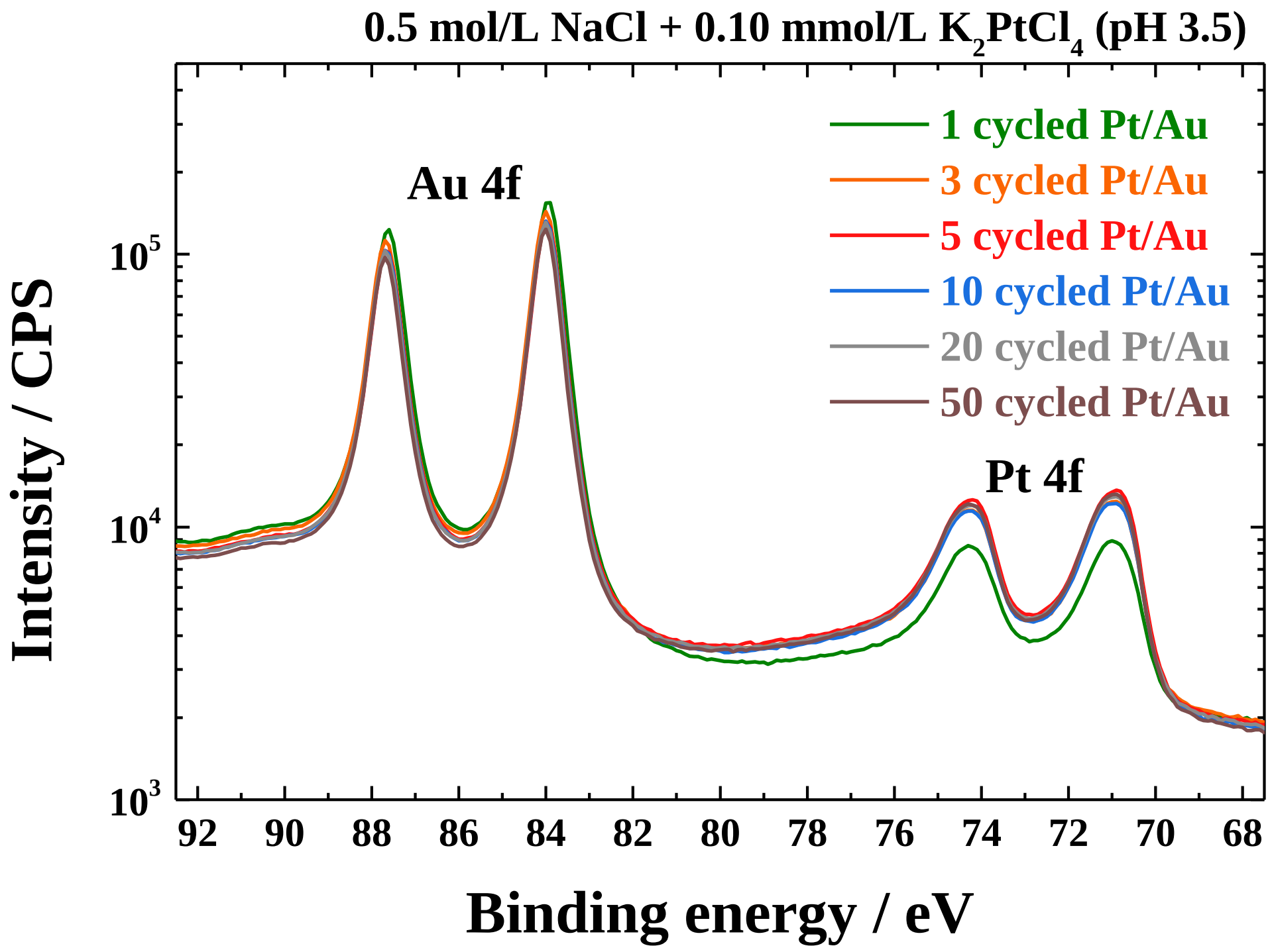

Fig. S20 Au 4f and Pt 4f spectra following consecutive 20 mV/s voltammetric cycles in 0.5 mol/L NaCl containing 0.10 mmol/L K_2PtCl_4 electrolyte. The quantitative parameters shown in **Figure 5b and 5c** were derived from these spectra.

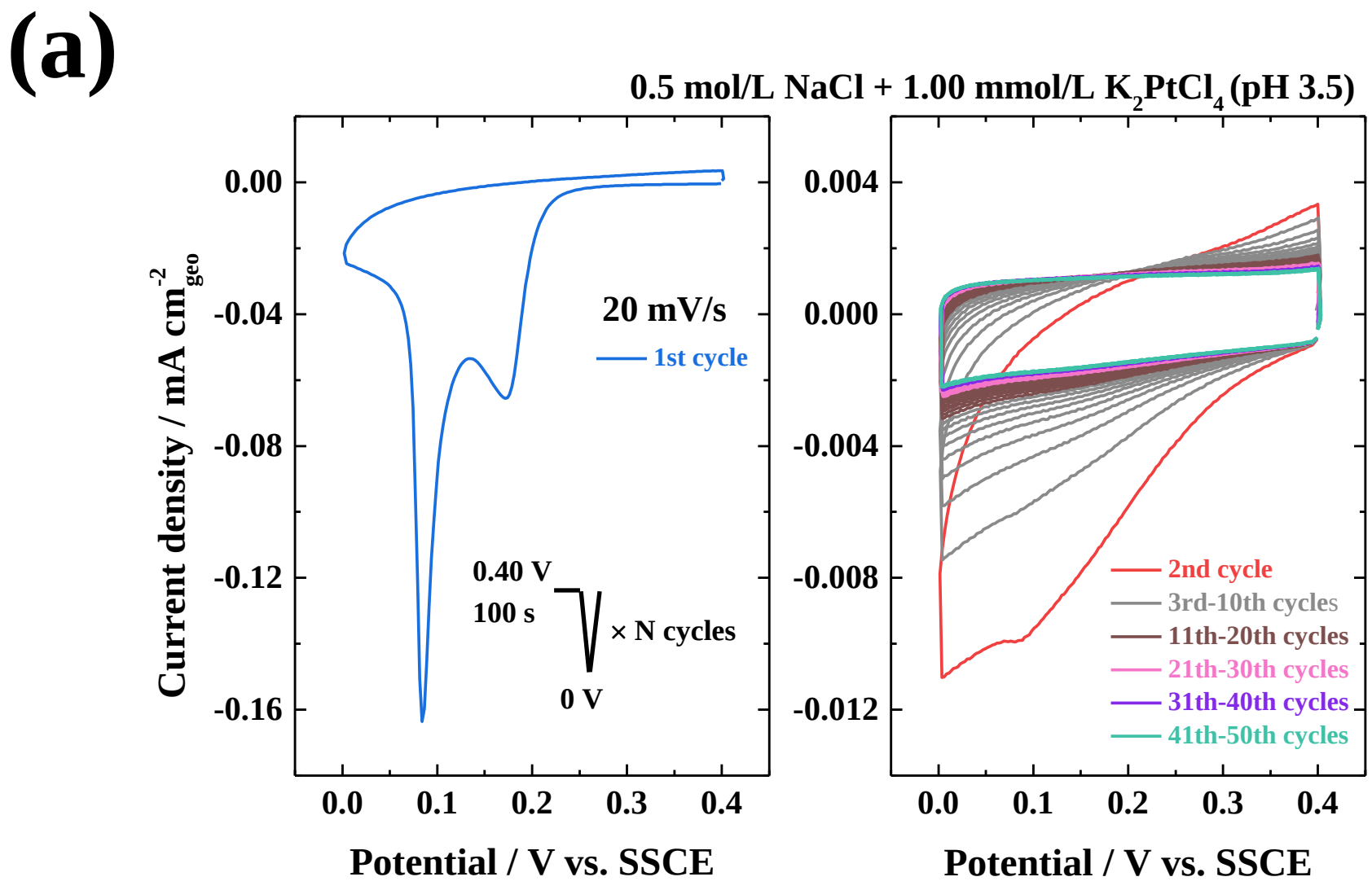

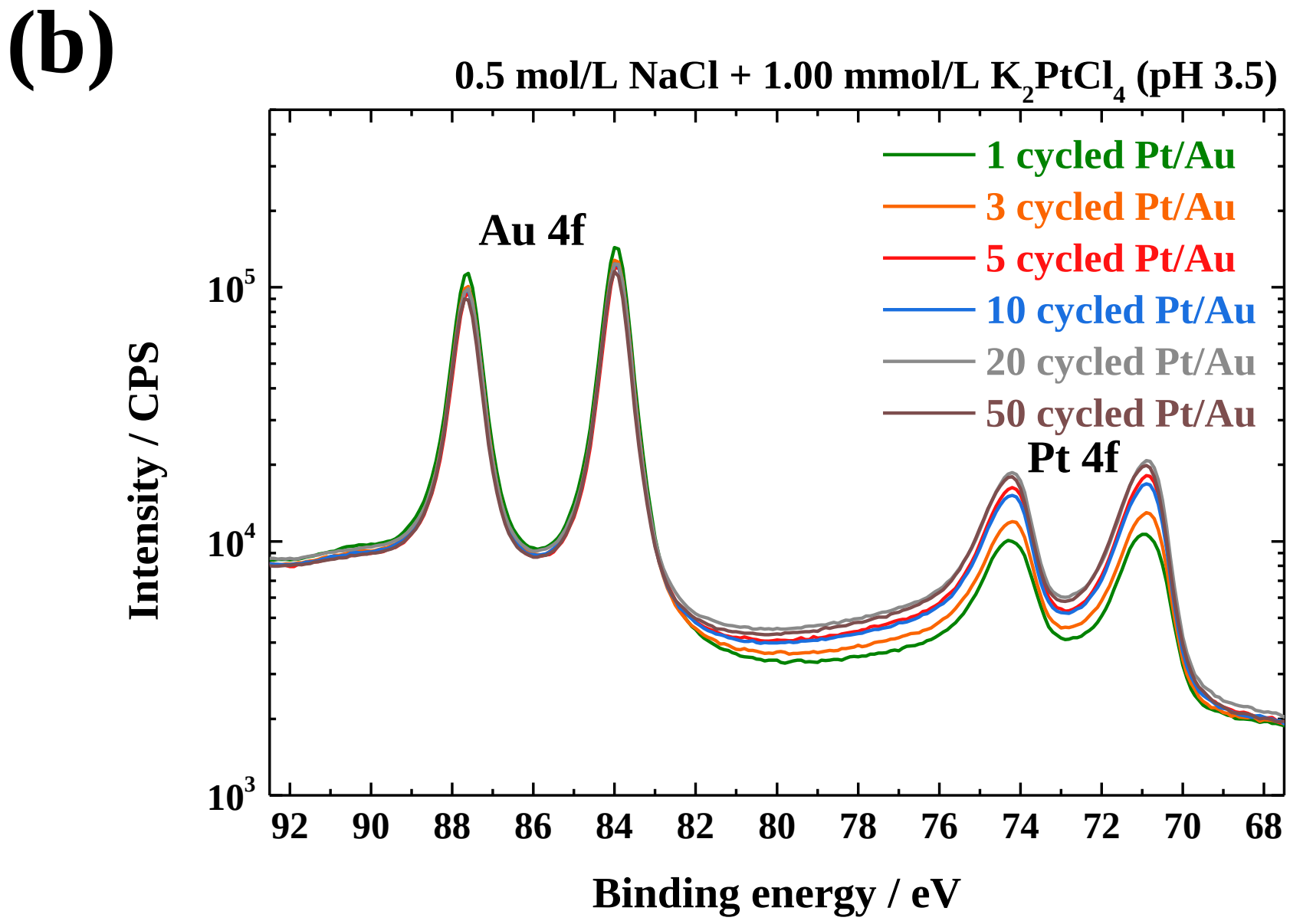

Fig. S21 (a) Fifty consecutive voltammogram initiated after 100 s dwell at +0.40 V_{SSCE} in 0.5 mol/L NaCl containing 1.00 mol/L K_2PtCl_4. (b) Au 4f and Pt 4f spectra following consecutive 20 mV/s voltammetric cycles in 0.5 mol/L NaCl containing 1.00 mmol/L K_2PtCl_4 electrolyte. The quantitative values shown in **Figure 7c** were derived from these spectra.

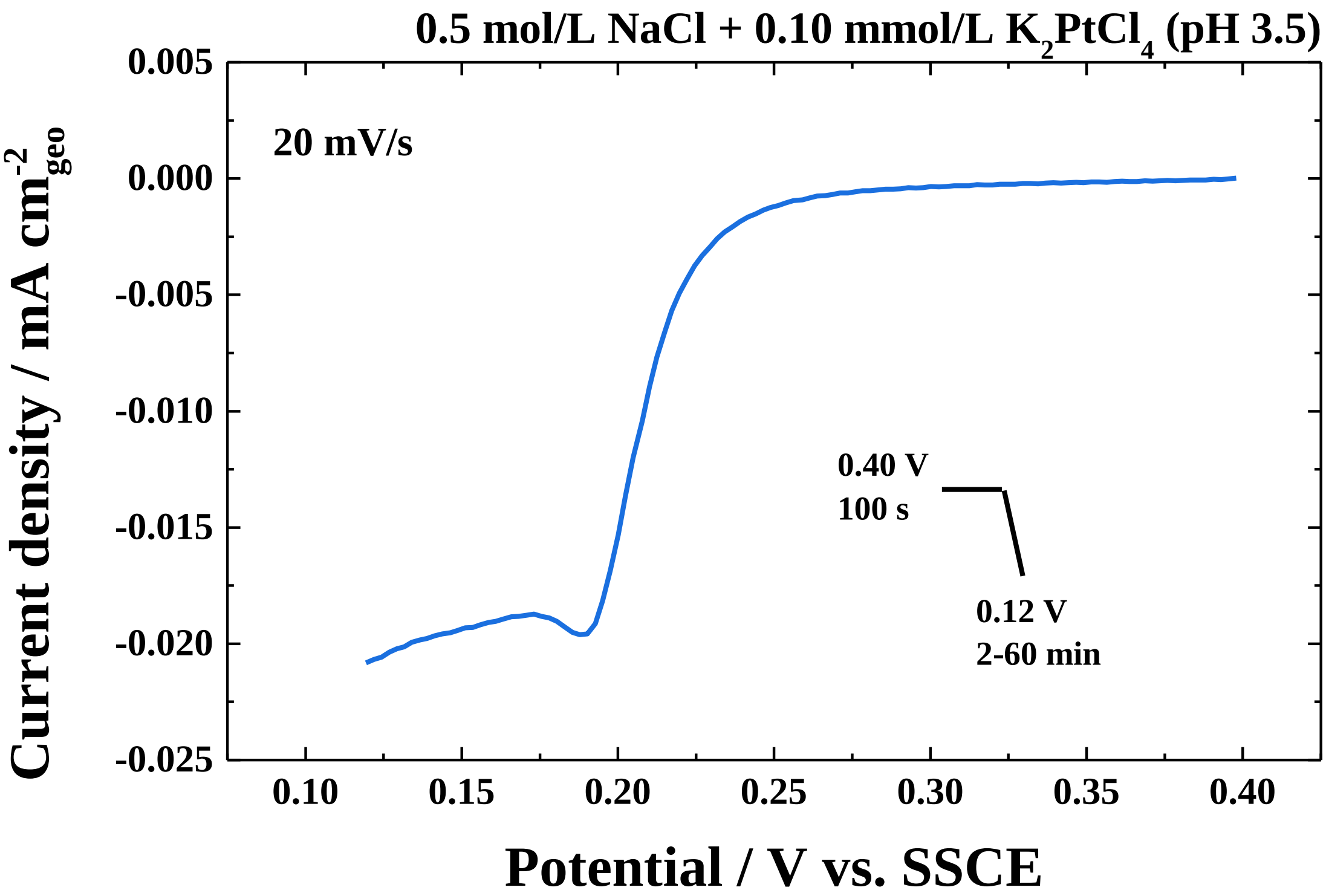

Fig. S22 Linear voltammetric scan initiated after 100 s dwell at 0.40 V_{SSCE} in 0.5 mol/L NaCl with 0.10 mmol/L K_2PtCl_4 and then held at +0.12 V_{SSCE} for variable amounts of time.

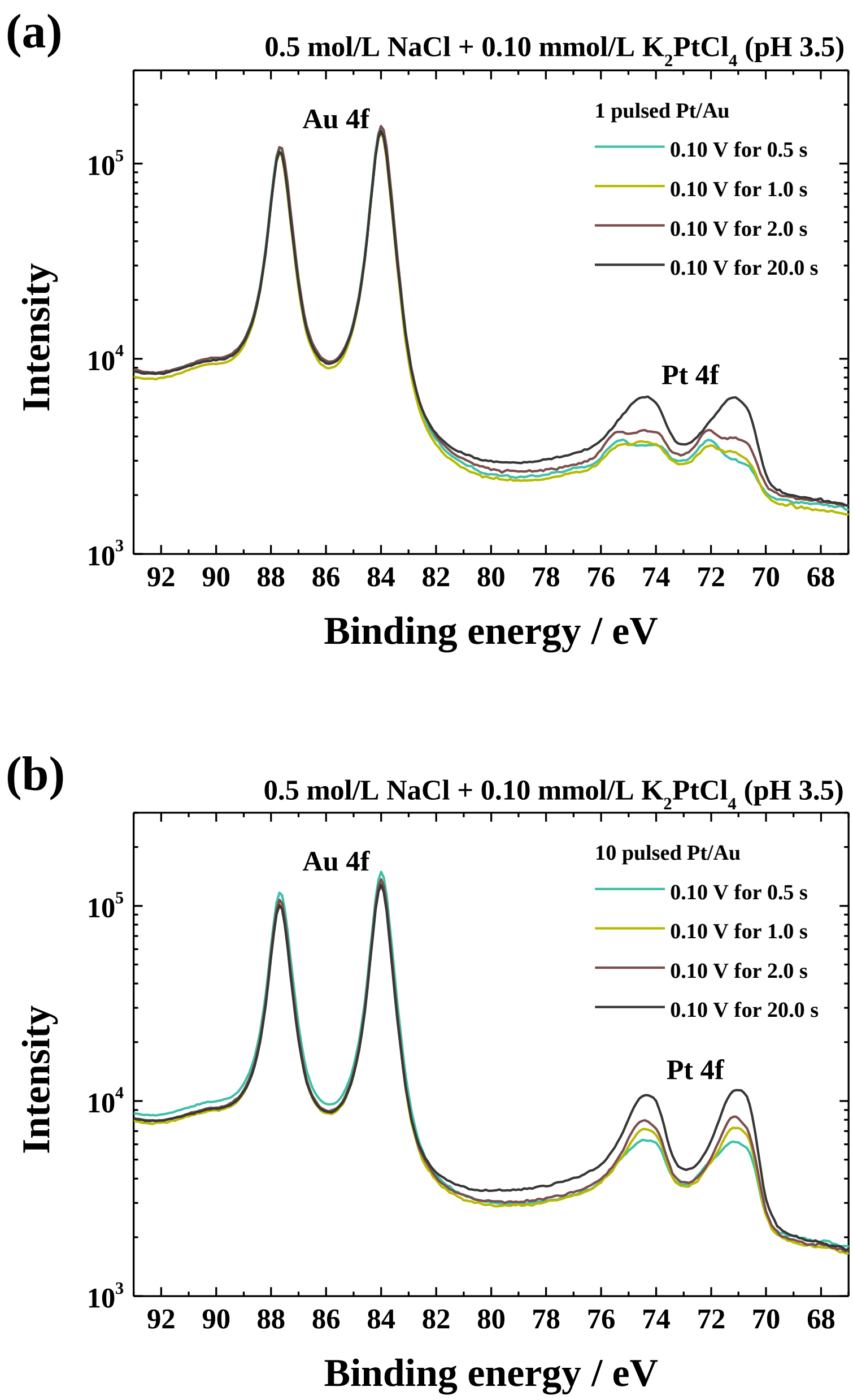

Fig. S23 Au 4f and Pt 4f spectra for a pulsed Pt/Au film as a function of dwell time at 0.10 V_{SSCE}. The electrodes were first derivatized at 0.40 V_{SSCE} for 100 s in 0.10 mmol/L K_2PtCl_4 containing 0.5 mol/L NaCl. The spectra of (a) single pulsed and (b) 10 pulsed Pt/Au film. The quantitative values shown in **Figure 8c** were derived from these spectra.

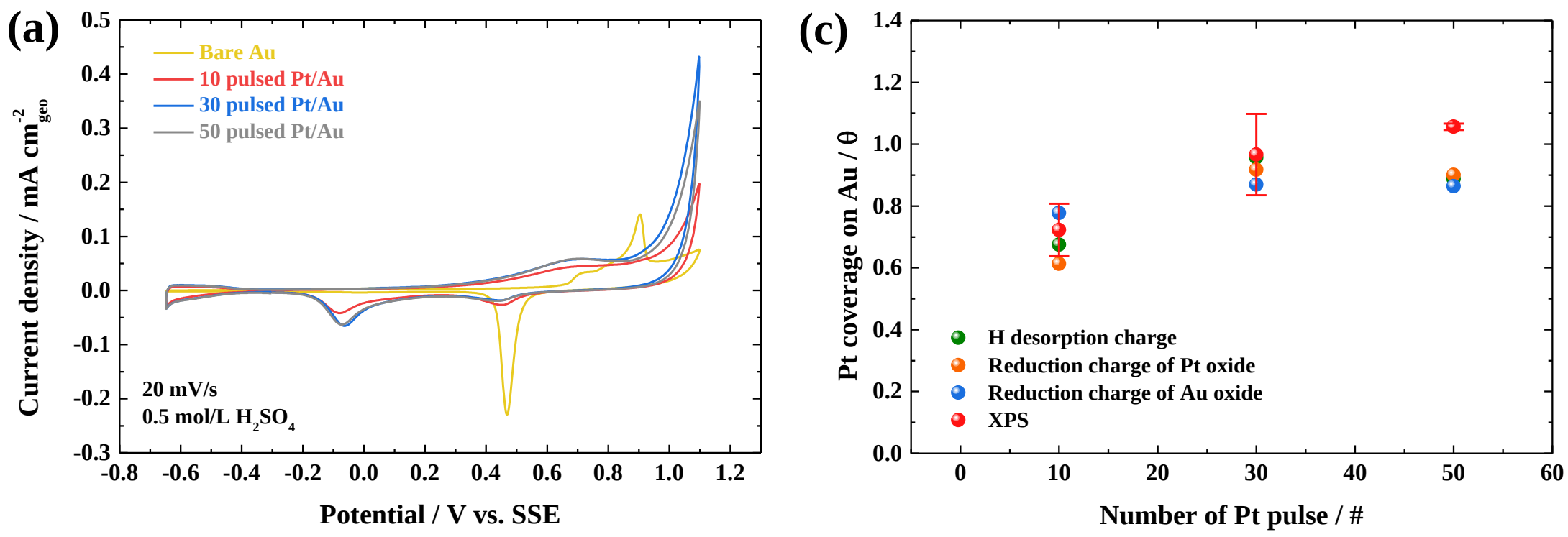

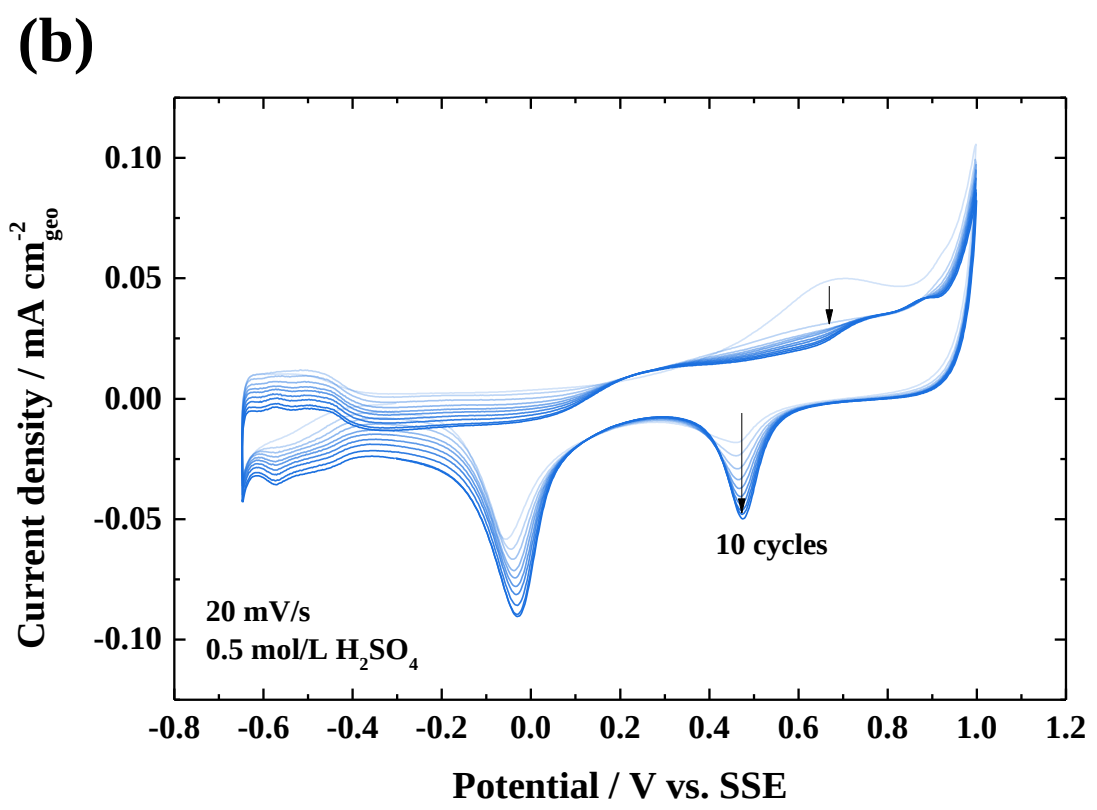

Sample	H desorption charge	Reduction charge of PtO_x to Pt	Reduction charge of AuO_x to Au
Au wafer	-	-	0.638 mC/cm^2 [θ_{Au}: 1]
10 pulsed Pt/Au	0.057 mC/cm^2 [θ_{Pt}: 0.713]	0.293 mC/cm^2 [θ_{Pt}: 0.610]	0.132 mC/cm^2 [θ_{Pt}: 0.793]
30 pulsed Pt/Au	0.078 mC/cm^2 [θ_{Pt}: 0.975]	0.435 mC/cm^2 [θ_{Pt}: 0.906]	0.079 mC/cm^2 [θ_{Pt}: 0.876]
50 pulsed Pt/Au	0.071 mC/cm^2 [θ_{Pt}: 0.888]	0.428 mC/cm^2 [θ_{Pt}: 0.892]	0.073 mC/cm^2 [θ_{Pt}: 0.886]

Fig. S24 Effective Pt coverage as revealed by H_{upd} charge and reduction of PtO_x and AuO_x charge as measured in 0.50 mol/L H_2SO_4. (a) First voltammetric cycle of the pulsed Pt/Au films. The scan was initiated at -0.30 V_{SSE}. (b) Ten consecutive cycles of 30 pulsed Pt/Au film. (c) Pt coverage of the multi pulsed Pt/Au films obtained by different calculation methods.

Due to the irreversible adsorption of $PtCl_4^{2-}$ on Au, residual complexes may persist on exposed Au in submonolayer Pt films and undergo further reduction within the H_{upd} potential range in H_2SO_4 electrolyte. To prevent overestimation of Pt coverage, only the H desorption charge was used for quantification. The integrated H desorption charge increased from 0.057

mC/cm^2 to 0.078 mC/cm^2 between films prepared by 10 and 30 deposition pulses, with no further increase observed for the 50 pulsed sample (**Fig. S24a**). Based on prior reports, the H_{upd} charge on Pt (111) reaches only 2/3 of the full monolayer value above 0.05 V_{RHE}, in contrast to Pt (100), which saturates at one monolayer.[14] Accordingly, a normalization value of 0.160 mC/cm^2 was used to estimate Pt coverage.

Sweeping to more positive potentials, the Pt film undergoes oxidation and place exchange between Pt and O followed by the oxidation and place exchange of any exposed Au sites. The subsequent reduction waves centered near (0.45 to 0.47) V_{SSE} and (-0.08 to -0.06) V_{SSE}, are attributed to sequential reduction of Au oxide and Pt oxide, respectively. The oxidation and reduction cycle lead to continuous roughening and intermixing of Pt and Au during cycling.[15,16] With continued cycling the AuO_x reduction peak continues to increase in line with the dynamic restructuring of the surface (**Fig. S24b**).

This perturbation builds upon the natural tendency of the lower surface energy Au to surface segregate on top of Pt.[17,18] The ratio of the two reduction waves yields an estimate of the Pt coverage following the oxidation cycle. The 30 and 50 pulsed samples display nearly identical voltammetric features, consistent with the saturation coverage behavior observed in XPS (**Fig. S24c**).

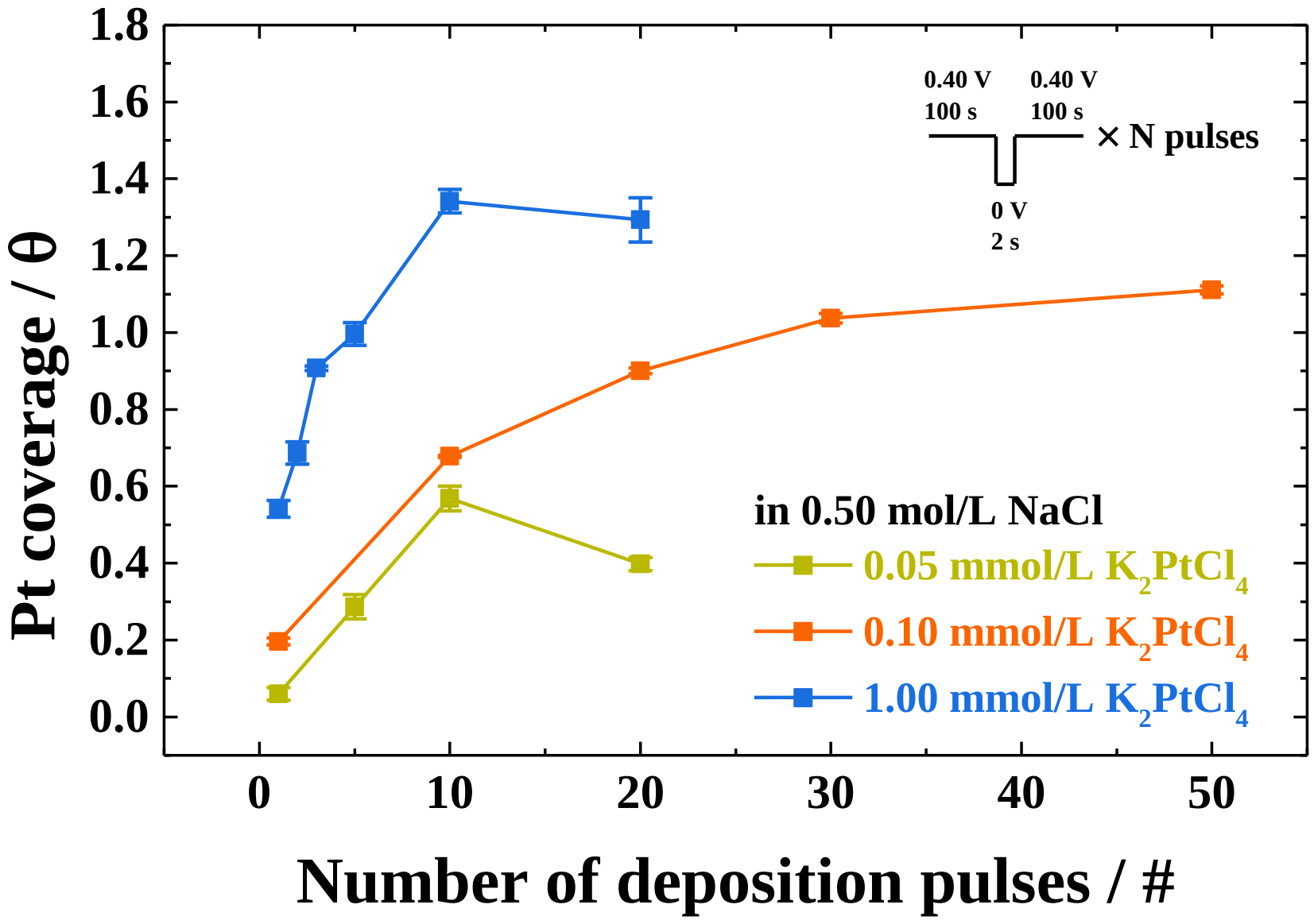

Fig. S25 Asymmetric pulse potential electrodeposition of Pt in 0.5 mol/L NaCl containing (0.05 to 1.0) mmol/L K_2PtCl_4 with the Pt coverage determined by XPS as a function of the number of pulses.

For experiments in 0.5 mol/L NaCl containing (0.05 to 1.0) mmol/L K_2PtCl_4, the potential is stepped between +0.40 V_{SSCE} for 100 s and 0 V_{SSCE} for 2 s for a variable number of cycles. For a dilute electrolyte with 0.05 mmol/L K_2PtCl_4, the Pt coverage increases with further pulsing but remains a sub-monolayer coverage even after 10 pulses. In contrast, pulse deposition in 1.0 mmol/L K_2PtCl_4 results in more rapid growth that exceeds a monolayer to saturating near 1.3 monolayer after 10 pulses as determined by XPS. A more gradual rate of deposition is observed in 0.10 mmol/L K_2PtCl_4 where a monolayer coverage is reached in less than 30 pulses, and asymptotically saturating near a 1.1 monolayer.

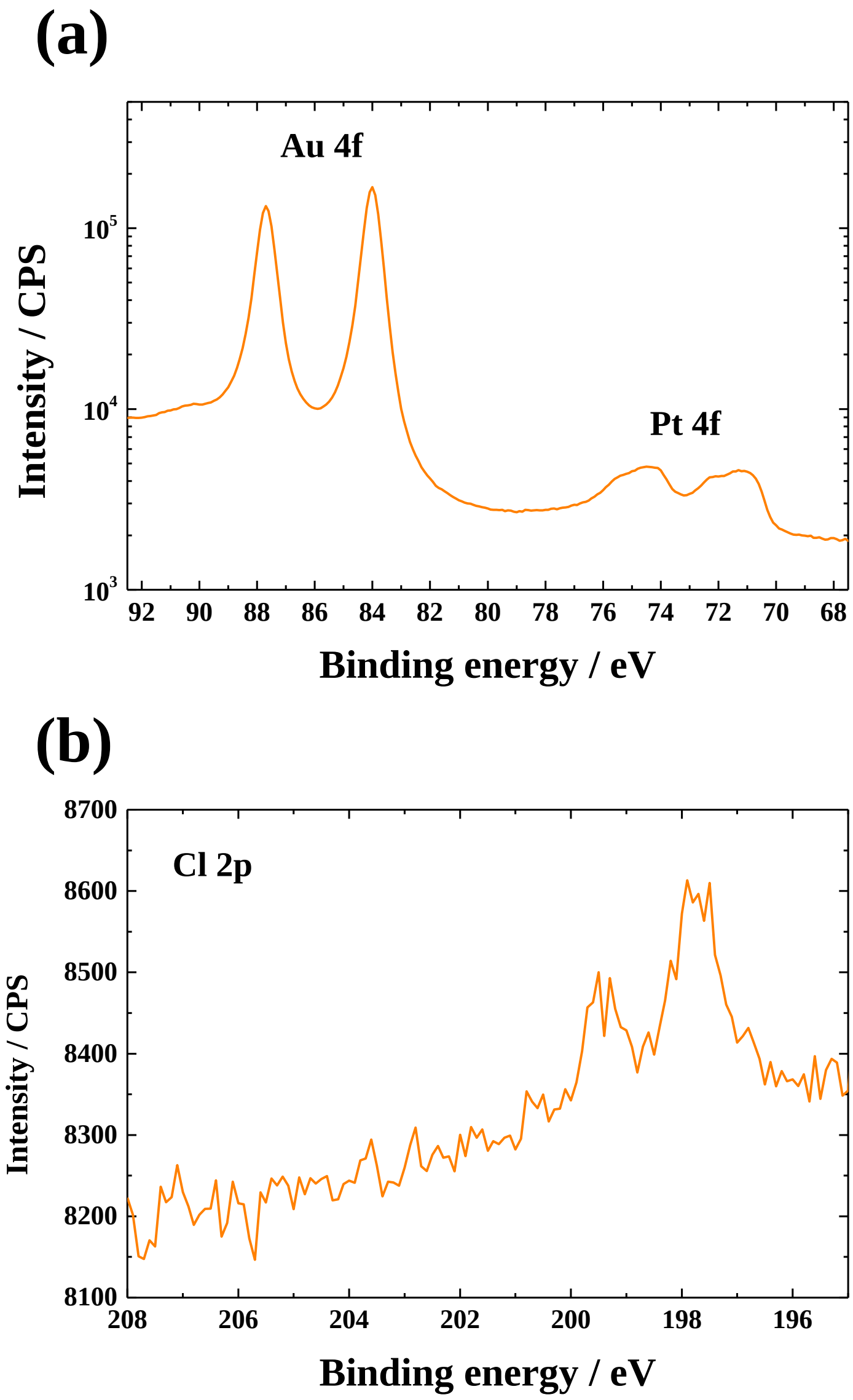

Fig. S26 (a) Pt 4f and (b) Cl 2p spectra for a single pulsed Pt/Au mica corresponding to the samples shown in **Fig. 9b,c**. The electrodes were first derivatized at 0.40 V_{SSCE} for 100 s in 0.10 mmol/L K_2PtCl_4 containing 0.5 mol/L NaCl, followed by a reduction step at 0.10 V_{SSCE} for 2 s and a subsequent hold at 0.40 V_{SSCE} for 100 s.

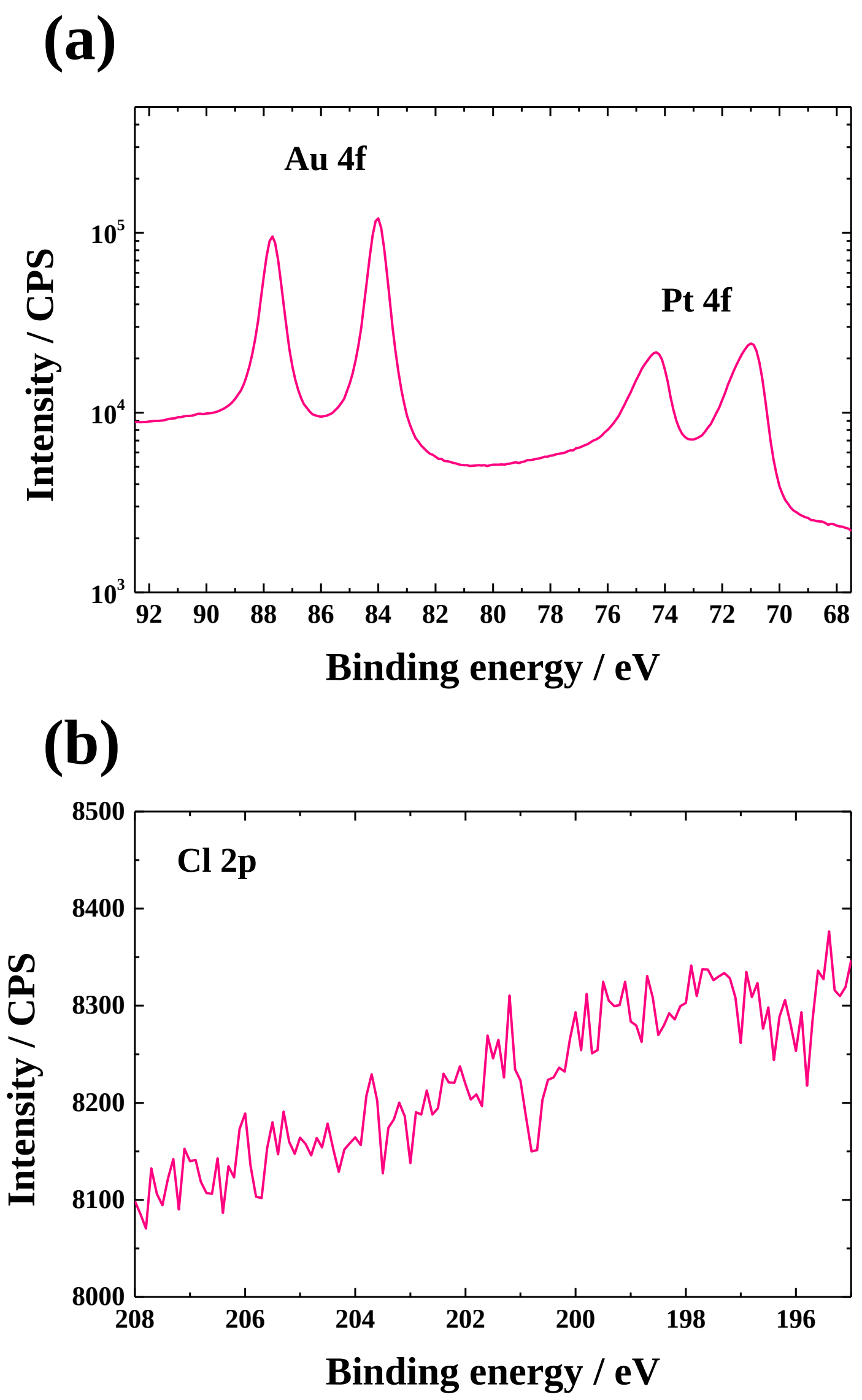

Fig. S27 (a) Pt 4f and (b) Cl 2p spectra for a single pulse Pt deposition on Au mica corresponding to the samples shown in **Fig. 10b,c**. The electrodes were first derivatized at 0.40 V_{SSCE} for 100 s in 0.10 mmol/L K_2PtCl_4 containing 0.5 mol/L NaCl, followed by a reduction step at 0.10 V_{SSCE} for 20 minutes and a subsequent hold at 0.40 V_{SSCE} for 100 s.

Reference

[1] Colombo, C.; Oates, C. J.; Monhemius, A. J.; Plant, J. A. Complexation of Platinum, Palladium and Rhodium with Inorganic Ligands in the Environment. *Geochem. Explor. Environ. Anal.* **2008**, *8*, 1–11. https://doi.org/10.1144/1467-7873/07-151

[2] Hubbard, A. T.; Anson, F. C. Study of the Electrochemistry of Chloride and Bromide Complexes of Platinum(II) and (IV) by Thin-Layer Electrochemistry. *Anal. Chem.* **1966**, *38*, 1887–1893. https://doi.org/10.1021/ac50155a052

[3] Lau, A. L. Y.; Hubbard, A. T. Study of the Kinetics of Electrochemical Reactions by Thin-Layer Voltammetry. III. *J. Electroanal. Chem.* **1970**, *24*, 237–249. https://doi.org/10.1016/S0022-0728(70)80148-6

[4] Smith, J. G.; Sawant, K. J.; Zeng, Z.; Eldred, T. B.; Wu, J.; Greeley, J. P.; Gao, W. Disproportionation Chemistry in K_2PtCl_4 Visualized at Atomic Resolution Using Scanning Transmission Electron Microscopy. *Sci. Adv.* **2024**, *10*, eadi0175. https://doi.org/10.1126/sciadv.adi017

[5] Seah, M. P. Simple Universal Curve for the Energy-Dependent Electron Attenuation Length for All Materials. *Surf. Interface Anal.* **2012**, *44*, 497–503. https://doi.org/10.1002/sia.5033

[6] Cumpson, P. J.; Seah, M. P. Elastic Scattering Corrections in AES and XPS. II. Estimating Attenuation Lengths and Conditions Required for Their Valid Use in Overlayer/Substrate Experiments. *Surf. Interface Anal.* **1997**, *25*, 430–446. https://doi.org/10.1002/(SICI)1096-9918(199706)25:6<430::AID-SIA254>3.0.CO;2-7

[7] Shard, A. G. Practical Guides for X-ray Photoelectron Spectroscopy: Quantitative XPS. *J. Vac. Sci. Technol. A* **2020**, *38*, 041201. https://doi.org/10.1116/1.5141395

[8] Raciti, D.; Braun, T.; Walker, A. R. H.; Moffat, T. P. Mapping Surface Chemistry During Superfilling with Shell-Isolated Nanoparticle Enhanced Raman Spectroscopy and X-ray Photoelectron Spectroscopy. *J. Electrochem. Soc.* **2022**, *169*, 082506. https://doi.org/10.1149/1945-7111/ac8023

[9] Seah, M. P. Quantification of AES and XPS. In *Practical Surface Analysis by Auger and X-ray Photoelectron Spectroscopy*; Briggs, D., Seah, M. P., Eds.; Wiley: New York, 1983.

[10] Hara, M.; Suto, K.; Inukai, J.; Yoshimoto, S.; Itaya, K. Adstructures of Platinum-Complex Precursors and Platinum Nanoparticles Formed on Low-Index Single Crystal Au Surfaces for Oxygen Reduction Reaction. *J. Electroanal. Chem.* **2020**, *875*, 114019. https://doi.org/10.1016/j.jelechem.2020.114019

[11] Chen, W.; Schmidt, D.; Schneider, W. F.; Wolverton, C. Ordering and Oxygen Adsorption in Au–Pt/Pt(111) Surface Alloys. *J. Phys. Chem. C* **2011**, *115*, 17915–17924. https://doi.org/10.1021/jp205995j

[12] Trembulowicz, A.; Sabik, A.; Jurczyszyn, L. Structural and Electronic Properties of Pt-Modified Au(100) Surface. *Sci. Rep.* **2022**, *12*, 3859. https://doi.org/10.1038/s41598-022-07617-2

[13] Dodge, D.; Dirks, R.; Hornbrook, L. F.; Hamidi, N.; Iski, E. V. In-Air STM Observation of Au(111) Surface Disturbance Including Au "Magic Fingers" as Modified by Solvent Choice. *Surf. Sci.* **2025**, *751*, 122629. https://doi.org/10.1016/j.susc.2024.122629

[14] Strmcnik, D.; Tripkovic, D.; van der Vliet, D.; Stamenkovic, V.; Markovic, N. M. Adsorption of Hydrogen on Pt(111) and Pt(100) Surfaces and Its Role in the HOR. *Electrochem. Commun.* **2008**, *10*, 1602–1605. https://doi.org/10.1016/j.elecom.2008.08.019

[15] Jacobse, L.; Rost, M. J.; Koper, M. T. M. Atomic Scale Identification of the Electrochemical Roughening of Platinum. *ACS Cent. Sci.* **2019**, *5*, 1920–1928. https://doi.org/10.1021/acscentsci.9b00782

[16] Behjati, S.; Koper, M. T. M. In Situ STM Study of Roughening of Au(111) Single-Crystal Electrode in Sulfuric Acid Solution During Oxidation–Reduction Cycle. *J. Phys. Chem. C* **2024**, *128*, 19024–19034. https://doi.org/10.1021/acs.jpcc.4c06362

[17] Pedersen, M. O.; Helveg, S.; Ruban, A.; Stensgaard, I.; Laegsgaard, E.; Nørskov, J. K.; Besenbacher, F. How a Gold Substrate Can Increase the Reactivity of a Pt Overlayer. *Surf. Sci.* **1999**, *426*, 395–409. https://doi.org/10.1016/S0039-6028(99)00385-4

[18] Abrams, B. L.; Vesborg, P. C. K.; Bonde, J. L.; Jaramillo, T. F.; Chorkendorff, I. Dynamics of Surface Exchange Reactions Between Au and Pt for HER and HOR. *J. Electrochem. Soc.* **2009**, *156*, B273–B282. https://doi.org/10.1149/1.3040509
